# Engineered Low‐Endotoxin Bacterial Biomimetic Vesicles for Enhanced Oral Dual‐Antigen Subunit Vaccine Delivery

**DOI:** 10.1002/jev2.70207

**Published:** 2025-11-29

**Authors:** Xuegang Shen, Shujie Wang, Kunying Qiu, Zeqing Liu, Xiaoxiao Tian, Fandan Meng, Yan‐Dong Tang, Haiwei Wang, Mingxia Sun, Xue‐Hui Cai, Tong‐Qing An, Yong‐Bo Yang

**Affiliations:** ^1^ State Key Laboratory for Animal Disease Control and Prevention, Harbin Veterinary Research Institute Chinese Academy of Agricultural Sciences Harbin China; ^2^ Heilongjiang Veterinary Biopharmaceutical Engineering Technology Research Center, Harbin Veterinary Research Institute Chinese Academy of Agricultural Sciences Harbin China; ^3^ Heilongjiang Provincial Key Laboratory of Veterinary Immunology, Harbin Veterinary Research Institute Chinese Academy of Agricultural Sciences Harbin China

**Keywords:** bacterial biomimetic vesicle, combination subunit vaccine, low‐endotoxin engineering, mucosal immunity, oral antigen delivery

## Abstract

Subunit vaccines are promising for disease prevention because of their safety and cost‐effectiveness. However, their efficacy is limited by low immunogenicity and gastrointestinal degradation after oral administration. To address this issue, low‐endotoxin *Salmonella choleraesuis* strain SC‐L3 was engineered via lipid A modification to generate bacterial biomimetic vesicles (BBVs) with reduced endotoxin activity. BBVs were functionalized using ClyA‐embedded SpyCatcher and Streptococcus protein G for dual antigen coupling, and further coated with chitosan oligosaccharides (COS) to enhance mucosal penetration and gastrointestinal stability. Using mCherry as a model antigen, we obtained optimized mCherry‐CSS‐BBV@COS that showed high antigen protection rates (83% and 63% in simulated gastric and intestinal fluids, respectively), capacity for lysosomal escape and effective stimulation of M1 macrophage polarization in vitro. Oral administration of mCherry‐CSS‐BBV@COS elicited robust systemic IgG and mucosal sIgA responses in mice. Furthermore, dual‐antigen BBV conjugates (GDH‐gD‐Fc‐CSS‐BBV@COS) co‐delivering *Streptococcus suis* glutamate dehydrogenase and pseudorabies virus gD‐Fc induced antigen‐specific humoral, mucosal and cellular immunity, conferring complete protection against lethal challenges with the respective pathogens. In summary, we generated a versatile, low‐endotoxin BBV platform for oral combination subunit vaccines, offering a novel strategy for protection against viral and bacterial infections.

## Graphical abstract

A genetically engineered low‐endotoxin *Salmonella choleraesuis* biomimetic vesicle (CSS‐BBV), coated with COS, is utilized to enhance the oral delivery efficacy of subunit vaccines. The modular SpyCatcher/SpG design enables rapid dual‐antigen conjugation, providing a versatile platform for the development of infectious disease vaccines.

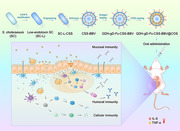

## 1 Introduction

Most emerging infectious diseases are zoonotic in nature (Jones et al. 2008; Taylor et al. 2001). Development of effective vaccines to control zoonotic diseases is crucial for global public health. The increasing variability of pathogens requires immunization. Compared with the effects of single vaccines, combination vaccines that integrate multiple antigens can simplify immunization procedures and reduce the number of doses, thereby lowering immunization costs. This is an inevitable trend in the future development of novel vaccines. Subunit/protein vaccines are becoming increasingly popular owing to their high efficacy, safety and low costs (Cao et al. 2018; Hervé et al. 2019). However, protein‐based antigens typically require adjuvants, injections and multiple immunizations to maintain long‐lasting protective immunity. In addition, immune‐related stress responses induced by injections have also garnered widespread attention (Taylor and Asmundson 2021). Oral vaccines demonstrate immune synergy and improve immunization compliance (Shukla et al. 2019; Farsaci et al. 2014; Coria et al. 2019; Love and Love 2021; Zhu and Berzofsky 2013). They effectively prevent pathogen infection by inducing mucosal secretory immunoglobulin A (sIgA) production (van Splunter et al. 2018; Qi et al. 2019; Chamcha et al. 2015). Despite being ideal for eliciting intestinal immunity, oral vaccination remains challenging owing to the properties of the gastrointestinal environment (Vela Ramirez et al. 2017), poor uptake of vaccine antigens by the intestinal epithelium (Devriendt et al. 2012) and tolerogenic environment pervading the gut (Spahn et al. 2001). To date, no oral subunit vaccine has been marketed, highlighting the urgent need for a combined subunit vaccine suitable for oral immunization.

Nanomaterials have extensive applications in subunit vaccines development (Wang et al. 2019; Chen et al. 2025; Baljon et al. 2024). Various conjugation systems have been used to load antigens onto nanocarriers. Among these, the SpyTag fusion protein is widely used because of its efficient covalent conjugate with SpyCatcher (Zhu et al. 2021; Nguyen et al. 2025; Hills et al. 2024; Jiang et al. 2022; Jia et al. 2025). In contrast, Fc‐fusion proteins extend protein half‐lives and enhance immune responses through Fc receptor‐mediated uptake and presentation mechanisms (Pridgen et al. 2013; Shubin et al. 2017). Intranasal administration of the respiratory syncytial virus F protein fused with IgG1 Fc induced high sIgA levels in the respiratory mucosa and activated a Th1‐type cellular immune response in mice (Zhang et al. 2019). By coupling Fc‐fusion proteins with high‐affinity *Streptococcus* protein G (SpG), applications of this system can be expanded further (Cho et al. 2017; Shen et al. 2017). The rational integration of dual‐conjugation systems into a single vector is a promising strategy to overcome the co‐delivery efficiency and immune response coordination challenges in combination subunit vaccine development.

The success of commercial meningococcal outer membrane vesicle (OMV) vaccines has garnered substantial interest in the use of bacteria‐derived OMVs as novel subunit vaccine delivery vehicles (Abara et al. 2024; Stejskal et al. 2024; Wang et al. 2024). OMVs are suitable for displaying multiple conjugation systems via genetic engineering, thereby embedding homologous or heterologous antigens (Cheng et al. 2021; Jin et al. 2024; van den Berg van Saparoea et al. 2018). They are non‐replicative nanoparticles that facilitate efficient uptake by dendritic cells (DCs) (Bai et al. 2025; Luo et al. 2024). Additionally, OMVs contain multiple bacteria‐derived components and various pathogen‐associated molecular patterns that facilitate antigen presentation and T cell activation (Feng et al. 2025; Chen et al. 2024). However, the limited yield of OMVs and loading efficiency of heterologous target molecules hinder their potential application as vaccine delivery vehicles.

High‐pressure homogenization can efficiently drive bacterial membranes to produce bacterial biomimetic vesicles (BBVs) with approximately 100 times higher yield than that of OMVs (Hua et al. 2021; Yang et al. 2021). Most importantly, the cargo load of BBVs is 30‐fold higher than that of OMVs (Hua et al. 2021; Yang et al. 2021). Like OMVs, BBVs have negatively charged surface (Li et al. 2023), which enables adsorption of cationic substances through electrostatic interactions. Chitosan oligosaccharide (COS), a cationic oligosaccharide, effectively protected against the adverse effects of astaxanthin under simulated gastrointestinal conditions (Liu et al. 2019). Moreover, COS significantly enhanced the gastrointestinal stability and intestinal retention time of oleanolic acid, leading to a 10.6‐fold increase in its oral bioavailability (Yuan et al. 2025). Therefore, COS‐coated BBVs are promising as an efficient oral combined‐vaccine delivery platform.

However, the use of bacteria‐derived biologics as subunit vaccine carriers is often challenging owing to high endotoxin activity (Thapa et al. 2023; Imamiya et al. 2023; Long et al. 2022). Lipid A is one of the key components that determines endotoxin activity of bacteria‐derived biological products (Bai et al. 2025; Long et al. 2022). Targeted modification of lipid A synthesis genes enables precise regulation of the numbers of fatty acid chains and phosphate groups (Bai et al. 2025; Irene et al. 2019; Liu et al. 2015), thereby reducing endotoxin activity. Therefore, low‐endotoxin‐modified BBVs are expected to be next‐generation bacterial membrane‐based nanoplatforms for subunit vaccine delivery.

*Salmonella choleraesuis* shows significant host adaptability in pigs and causes systemic human infections following consumption of contaminated pork products or exposure to contaminated water (Soliani et al. 2023). It is an intracellular pathogen that actively invades intestinal epithelial cells and Peyer's patches (Hou et al. 2025). This strain directly interacts with the gut‐associated lymphoid tissue and effectively activates mucosal immune responses (Huang et al. 2024; Li et al. 2023), making it an ideal candidate for preparing oral BBVs carriers. *Streptococcus suis* serotype 2 (*S. suis* 2) and pseudorabies virus (PRV) are common pathogens found on pig farms, and their combined infections may exacerbate the disease (Yu et al. 2021). *S. suis* 2 is a gram‐positive bacterium that causes severe infections in humans and pigs (Goyette‐Desjardins et al. 2014; Wang et al. 2020). PRV mainly infects swine, and potentially threatens the biosafety of other animals and humans (Wong et al. 2019; He et al. 2019). Extracellular and intracellular pathogens exhibit significant differences in host immune responses (Wilson et al. 2009; Glimcher and Murphy 2000), providing a foundation for selecting *S. suis* 2 and PRV as models to study how combination vaccines can simultaneously activate immune responses against both bacterial and viral infections.

**FIGURE 1:**
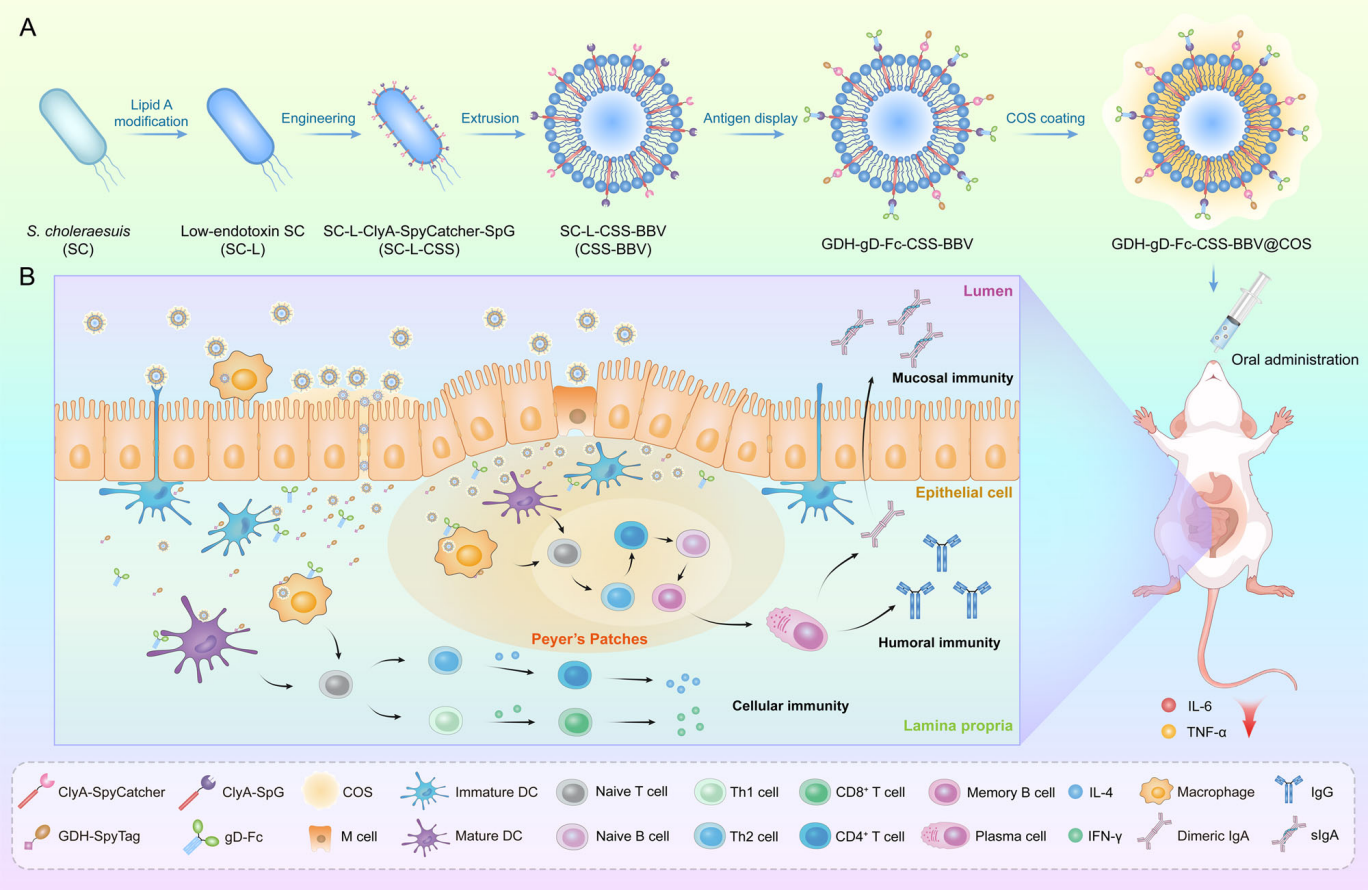
Engineering and immunological action mechanism of COS‐coated BBVs for oral dual‐antigen delivery. (A) Schematic illustration of GDH‐gD‐Fc‐CSS‐BBV@COS preparation. (B) COS‐coated GDH‐gD‐Fc‐CSS‐BBV@COS traverses the epithelial barrier and significantly reduce the inflammatory response, stimulate macrophages and epithelial cells within lamina propria and initiate adaptive immune responses. GDH‐gD‐Fc‐CSS‐BBV@COS is preferentially captured by microfold (M) cells in the Peyer's patches. Processed antigens are subsequently presented by mature dendritic cells (DCs) to naive T cells, driving differentiation into T helper 1 (Th1), T helper 2 (Th2) and cytotoxic CD8⁺ T cells. Simultaneously, naive B cells are activated in the B cell follicles, triggering the germinal centre reaction, plasma cell differentiation and antibody production (including IgG and dimeric IgA). Key cytokines involved (IFN‐γ, IL‐4) and secretory IgA (sIgA) transport are indicated.

In this study, we generated genetically engineered BBVs that featured a penta‐acylated monophosphoryl lipid A structure that significantly reduced endotoxin activity and mitigated systemic pro‐inflammatory cytokine responses. Additionally, a plug‐and‐display approach was used to co‐display GDH‐SpyTag and gD‐Fc fusion proteins on the BBV surface, followed by COS coating (Figure 1A). As a universal nanocarrier, CSS‐BBV efficiently bound dual antigens, enhancing their uptake by immune cells and promoting DCs maturation. Oral administration of GDH‐gD‐Fc‐CSS‐BBV@COS elicited robust antigen‐specific humoral, mucosal and cellular immune responses in vivo (Figure 1B), providing complete protection against lethal doses of *S. suis* 2 and PRV. Overall, our findings demonstrated that genetic engineering modifications to lipid A are an effective strategy for reducing the endotoxin activity of BBVs. The obtained CSS‐BBV‐based oral combination vaccine delivery system has significant application potential for flexible antigen presentation.

## 2 Materials and Methods

### 2.1 Materials

African green monkey kidney cells (Vero E6 cells), RAW264.7 cells, *Escherichia coli* strain BL21‐DeE, *S. choleraesuis* strain SC014, PRV strain HLJ8, *S. suis* serotype 2 strain 700794 and prokaryotic expression plasmids pCold I and pET28a^+^‐ClyA were maintained in our laboratory (Harbin, China). PrimeSTAR Max DNA Polymerase (R045Q), PrimeScript RT reagent (RR037A) and RT‐qPCR kits were obtained from Takara (Tokyo, Japan). Seaming Cloning Kit (BL1046B) and Cell Counting Kit‐8 (CCK‐8) were acquired from Biosharp (Hefei, China). DH5α competent cells were sourced from Sangon Biotech (Shanghai, China). The E.Z.N.A. Total RNA (R6834‐02), E.Z.N.A. Gel Extraction (D2500‐02) and Plasmid Mini Kits (D6943‐03) were purchased from Omega Bio‐Tek (Norcross, GA, USA). The pre‐stained protein ladder (26617), interleukin (IL)‐6 ELISA Kit (KMC0061), tumour necrosis factor (TNF‐α) ELISA Kit (BMS607‐3), Ni‐NTA Agarose (R901‐10) and Pierce Bicinchoninic Acid Protein Assay Kit (23225) were obtained from Thermo Fisher Scientific (Waltham, MA, USA). The anti‐His‐tag mouse monoclonal antibody (B1004) was purchased from Biodragon (Suzhou, China). Dulbecco's modified Eagle's medium (DMEM; D6429) and foetal bovine serum (12103C) were purchased from Sigma‐Aldrich (St. Louis, MO, USA). COS (S31060) was purchased from Yuanye Biotechnology (Shanghai, China). Simulated gastric fluid (SGF, SL6600) and simulated intestinal fluid (SIF, SL66102) were obtained from Coolaber (Beijing, China). A FITC‐conjugated anti‐mouse CD206 antibody (FMF206‐01‐025) was purchased from 4A Biotech (Beijing, China). PE‐conjugated anti‐mouse CD86 (50‐0862‐U025), PerCP‐Cyanine5.5‐conjugated anti‐mouse CD3, FITC‐conjugated anti‐mouse CD4 and PE‐conjugated anti‐mouse CD8a antibodies were purchased from Tombo Biosciences (San Diego, CA, USA). Goat HRP‐conjugated anti‐mouse IgG (C31430100), goat HRP‐conjugated anti‐mouse IgA (1040‐05), FITC‐conjugated anti‐mouse CD80 (F2108001) and APC‐conjugated anti‐mouse CD11c (F21011C03) antibodies were purchased from Multisciences (Hangzhou, China). Goat HRP‐conjugated anti‐mouse IgG1 (1071‐05) and goat HRP‐conjugated anti‐mouse IgG2a (1080‐05) antibodies were purchased from Southern Biotech (Birmingham, AL, USA). The mouse spleen lymphocyte separation medium kit (LTS1092PK) was purchased from TBD (Tianjin, China). The uncoated mouse IL‐4 (E‐UNEL‐M0068), uncoated mouse interferon‐gamma (IFN‐γ, E‐UNEL‐M0043) and Mouse iNOS (E‐EL‐M0696) ELISA kits were purchased from Elabscience Biotechnology (Wuhan, China). Granulocyte‐macrophage colony‐stimulating factor (GM‐CSF, RP01206) and recombinant mouse IL‐4 protein (RP01161) were purchased from Abcam (Cambridge, UK).

### 2.2 Construction of Plasmids and Deletion Mutants

Genes encoding ClyA‐mCherry‐6×His, ClyA‐SpyCatcher‐6×His and ClyA‐SpG‐6×His were individually cloned into the pCold I vector. Additionally, three repeat flexible linkers (Gly_4_Ser)_3_ were inserted downstream of ClyA to construct plasmids pCold I‐ClyA‐mCherry, pCold I‐ClyA‐SpyCatcher and pCold I‐ClyA‐SpG. These constructs facilitated the display of mCherry, SpyCatcher and SpG, respectively, on the surface of the bacteria and corresponding BBVs. mCherry‐SpyTag and GDH‐SpyTag were cloned into the pET28a vector to generate the plasmids pET28a‐mCherry‐SpyTag and pET28a‐GDH‐SpyTag, which were used for the expression and purification of mCherry‐SpyTag and GDH‐SpyTag proteins, respectively.

The gene knockout procedure was conducted according to a previously established method with minor modifications (Bian et al. 2024; Liu et al. 2020). The primers used in this study are listed in Table S1. The upstream and downstream fragments were amplified using the primer pairs DtolB‐1F/DtolB‐1R and DtolB‐2F/DtolB‐2R, respectively. Then, the mutant *tolB* gene fragment, which contained upstream and downstream homologous arms, was ligated to pRE112 to construct recombinant suicide plasmid P1 (Δ*tolB*). Similar procedures were used to prepare plasmids P2 (Δ*pagP*) and P3 (Δ*msbB*), which were designed to delete the *pagP* and *msbB* genes, respectively. The insertions of the *lpxE* and *pagL* genes were amplified with primers DpagP‐P2‐F/R and DlpxE‐F/R using P2 and pQK005 plasmid DNA as templates, respectively. The amplified fragments were ligated into pRE112 to obtain P4 (::lpxE) and P5 (::pagL). For the knockout strain construction, recombinant suicide plasmids P1–P5 were transformed into the S17‐1λpir strain and subsequently mobilized into *S. choleraesuis* strain SC014 (SC) by conjugation. Through allele exchange and sucrose selection, mutants SC‐L1 (Δ*tolB*), SC‐L2 (Δ*tolB*Δ*pagP*::*lpxE*) and SC‐L3 (Δ*tolB*Δ*pagP*Δ*msbB*::*lpxE*::*pagL*) were successfully obtained.

### 2.3 Characterization of Mutant Strains

Surface hydrophobicity was calculated using bacterial adhesion to hydrocarbons method (Farid et al. 2021). Xylene was separately added to the resuspended SC, SC‐L1, SC‐L2 and SC‐L3 cultures, and a blank control was used. After mixing, the emulsion was allowed to stand for 15 min to separate the aqueous phase, and the optical density at 600 nm (OD_600_) was measured. Surface hydrophobicity was calculated using the following formula:

Surface hydrophobicity (%) = (Control OD_600_ − Experimental OD_600_)/Control OD_600_) × 100%. The experiment was independently repeated thrice. The bacterial suspensions were collected, centrifuged and washed with phosphate‐buffered saline (PBS). The initial optical density (A0) and optical density after 5 h of incubation (Ai) were recorded. The self‐aggregation rate was calculated using the formula:

$$\mathrm{Self}-\mathrm{aggregation}\;\mathrm{rate}\;\left(\%\right)=\left(A0-\mathrm{Ai}\right)/A0\times 100\%.$$

The growth curve was determined by collecting exponentially growing bacteria at different time points, using three biological replicates.

### 2.4 Lipid A Isolation and Identification by Mass Spectrometry

After culturing the SC and SC‐L3 strains at an OD_600_ of 1.0, the cultures were centrifuged and washed with PBS. The precipitate was resuspended in a mixture of chloroform, methanol and water (1:2:0.8 v/v), stirred for 1 h, and centrifuged to remove the soluble substances. Sodium acetate (12.5 mM, pH 4.5) was added for ultrasonication and heating to facilitate the release of lipid A from LPS. After cooling, the solution was mixed for 1 h with the same mixture used for extraction. Following centrifugation, the lower layer containing lipid A was transferred to a culture flask, dried with through a rotary evaporator, and dissolved in a mixture of chloroform and methanol, then stored at −80°C. For negative‐ion matrix‐assisted laser desorption/ionization with a time‐of‐flight mass spectrometry experiments, dried lipids were dissolved in chloroform/methanol (4:1, v/v), and the norharmane matrix was added to each sample. The analysis was conducted using a Bruker Autoflex Speed mass spectrometer with the laser power set to 50% and an average of 500 shots per spectrum. Calibration was performed using an electrospray ionization tuning mixture and norharmane matrix.

### 2.5 Purification of Lipopolysaccharide and Stimulation of RAW264.7 Mouse Macrophages

Lipopolysaccharide (LPS) purification was optimized as previously described (Shen et al. 2024). The SC and SC‐L3 strains were inoculated into LB medium at a ratio of 1:100 and cultured until the OD_600_ reached ∼0.9. Bacterial cells were collected using centrifugation, washed with PBS, resuspended and subjected to ultrasonication. After adding proteinase K (PK) and incubating the mixture at 65°C for 1 h, 20% MgSO_4_/chloroform (1:4, v/v), RNase (50 µg/mL) and DNase (200 µg/mL) were added, and the mixture was incubated at 37°C overnight. On the following day, preheated phenol was added, followed by shaking, cooling on ice and centrifugation. Finally, LPS was precipitated using anhydrous ethanol, dialyzed to remove phenol and lyophilized for storage. The extracted LPS were identified by silver staining and designated as SC‐LPS and SC‐L3‐LPS. LPS was quantified using an optimized phenol‐sulfuric acid colorimetric method. In this process, polysaccharides are hydrolysed into monosaccharides at elevated temperatures and subsequently react with phenol to produce orange derivatives. Various concentrations of glucose were combined with 5% phenol and concentrated sulfuric acid, followed by incubation for 30 min, after which the absorbance was measured at OD_490_. A standard curve was constructed by plotting the glucose content on the x‐axis and OD_490_ values on the y‐axis. The same method was used to determine the LPS concentration.

Mouse RAW264.7 macrophages were cultured in DMEM supplemented with 10% foetal bovine serum and antibiotics at 37°C in the atmosphere of 95% air and 5% CO_2_. After an initial 24 h of culture, cells were seeded in a 96‐well plate at a density of 1 × 10^5^ per well and stimulated with 1 µg/mL LPS for an additional 24 h. TNF‐α and IL‐6 levels were quantified using qPCR and ELISA kits.

### 2.6 Construction and Expression of Recombinant Expression Bacteria

The pCold I‐ClyA‐mCherry, pCold I‐ClyA‐SpyCatcher, pCold I‐ClyA‐SpG and pCold I‐mCherry plasmids were introduced into the SC‐L3 mutant strain, successfully generating recombinant strains designated as SC‐ClyA‐mCherry, SC‐ClyA‐SpyCatcher‐SpG (SC‐L‐CSS) and SC‐mCherry, respectively. The recombinant strains were cultured at 37°C and 220 r/min until the OD_600_ reached approximately 0.6. Then, 0.1 mM isopropyl β‐D‐thiogalactopyranoside (IPTG) was added, and the culture was incubated at 16°C and 200 r/min for 14 h to induce protein expression. Subsequently, the samples were analysed using Coomassie Brilliant Blue staining or Western blotting.

### 2.7 Validation of mCherry Localization on the Surface of Bacteria

After protein expression induction with IPTG, SC‐ClyA‐mCherry and SC‐mCherry suspensions were collected, treated sequentially with an anti‐mCherry antibody, followed by a FITC‐labelled secondary antibody, and washed three times with PBS containing 1% bovine serum albumin. A control without the fluorescent antibodies was used. Finally, the fluorescence signals of the samples were analysed using flow cytometry, and the diluted suspension was dropped onto slides coated with 0.3% agarose. Images were captured using a confocal laser scanning microscope. Additionally, IPTG‐induced SC‐ClyA‐mCherry and SC‐mCherry bacterial suspensions were subjected to ultrasonication and incubated with pre‐chilled 1% Triton X‐114 at 4°C for 1 h. The solution was heated to 37°C for 15 min and centrifuged to separate the hydrophilic and hydrophobic phases. Proteins in both phases were precipitated using chloroform/methanol (1:4, v/v), with proteins from the hydrophobic phase representing the extracted outer membrane proteins. Finally, proteins were detected using sodium dodecyl sulphate–polyacrylamide gel electrophoresis (SDS‐PAGE).

### 2.8 Purification and Characterization of BBVs

SC, SC‐L3, SC‐mCherry, SC‐ClyA‐mCherry and SC‐L3‐CSS cells were cultured to an OD_600_ of approximately 0.6. Then, except for SC and SC‐L3, 0.1 mM IPTG was added to induce recombinant protein expression at 16°C. The following day, 2 mM Na_2_EDTA was introduced, and the cells were cultured was continued for an additional 2 h, centrifuged at 10,000 × g for 30 min at 4°C, washed with PBS and subjected to high‐pressure homogenization at 1200 bar at 4°C thrice. Finally, the supernatant was collected following centrifugation at 9000 × g, filtered through a 0.45 µm membrane, and the filtrate was subjected to size‐exclusion chromatography to obtain SC‐BBV, SC‐L3‐BBV, BBV‐mCherry, BBV‐ClyA‐mCherry and CSS‐BBVs, respectively. Transmission electron microscopy, NanoZS90 nanoparticle size analyser and chromogenic limulus amebocyte lysate endotoxin detection kit was used to assess the morphology, particle size, zeta potential and endotoxin activity of BBVs obtained. SC‐BBV and SC‐L3‐BBV were used to stimulate RAW264.7 cells to evaluate the expression levels of TNF‐α and IL‐6. BBV‐mCherry and BBV‐ClyA‐mCherry were incubated with 0.1 mg/mL PK for 30 min at 37°C to hydrolyse proteins exposed on the membrane surface. The effect of PK treatment on the mCherry protein band was analysed using Western blotting.

### 2.9 Protein Expression and Purification

mCherry‐SpyTag and GDH‐SpyTag were expressed in BL21‐DeE cells characterized by low endotoxicity. Initially, the pET28a‐mCherry‐SpyTag and pET28a‐GDH‐SpyTag plasmids were individually transformed into BL21‐DeE cells and cultured in LB supplemented with 50 µg/mL kanamycin at 37°C for 12 h. Subsequently, the culture was inoculated at a ratio of 1:100 and grown until the OD_600_ reached ∼0.6. Then, 0.5 mM IPTG was added, and the culture was incubated at 16°C with shaking at 200 r/min for 16 h to induce protein expression. Additionally, the recombinant gD‐Fc protein was produced using the BHK‐21 cell line, which was constructed and preserved at the laboratory. The samples were then centrifuged at 12,000 × g for 20 min at 4°C. The supernatant was then filtered through a 0.22‐µm filter and subsequently loaded into a gravity‐flow column containing Ni‐NTA chelated agarose. Target proteins were eluted using an elution buffer (50 mM Tris‐HCl, 100 mM NaCl, 5% glycerol and 200 mM imidazole, pH 8.0). The collected target proteins were subsequently analysed by Coomassie Brilliant Blue staining or Western blotting. Finally, the target protein concentrations were determined using a bicinchoninic acid protein assay kit, and the proteins were stored at −80°C for future use.

### 2.10 Binding and Characterization of CSS‐BBV With SpyTag and Fc Fusion Proteins

Prior to mixing, the buffer for each component was replaced with a binding buffer (20 mM Tris‐HCl, 50 mM NaCl, pH 8.0) using dialysis bags. The input amount of the target protein was kept constant and CSS‐BBV was subsequently added at various concentrations. The mixture was stirred gently overnight at 4°C. To eliminate non‐adsorbed target proteins, a centrifugal ultrafiltration device with a molecular weight cutoff of 100 kDa was used. The dual antigen conjugation process was divided into two steps. First, CSS‐BBV was combined with GDH‐SpyTag to obtain GDH‐SpyTag‐CSS‐BBV, named GDH‐CSS‐BBV. To obtain GDH‐gD‐Fc‐CSS‐BBV, GDH‐CSS‐BBV was combined with gD‐Fc using the same binding method. The conjugation between CSS‐BBVs and the target protein was assessed using Western blotting, immunoelectron microscopy and particle size analysis. Grayscale simulation analysis was conducted using ImageJ software to quantify the electrophoretic band intensity of mCherry‐SpyTag.

The efficiency of conjugation of GDH‐CSS‐BBV with gD‐Fc was calculated by quantifying the protein input and free protein after ultrafiltration. The specific steps were as follows: after GDH‐CSS‐BBV binding to gD‐Fc, centrifugation (2000 × g) was performed using an ultrafiltration centrifuge tube, and the OD value of gD‐Fc at the bottom of the ultrafiltration tube at 280 nm was detected using a NanoDrop. The remaining amount of gD‐Fc was determined by a standard curve established within the linear range of OD values at 280 nm using protein standards.

The conjugation efficiency of CSS‐BBV with the target protein was calculated using the following formula:

$$\begin{array}{ccc} & & \mathrm{Conjugation}\;\mathrm{efficiency}\;\left(\%\right)\\ & & =\left(\mathrm{input}\;\mathrm{amount}-\mathrm{remaining}\;\mathrm{amount}\right)/\mathrm{input}\;\mathrm{amount}\times 100\%. \end{array}$$

Additionally, a nano‐flow cytometer (Micro Plus, Apogee) was used to analyse the conjugation effect. Mixed samples of mCherry conjugated with CSS‐BBV (1 and 3.5 mg/mL) were subjected to flow cytometry, with CSS‐BBV without the conjugated protein used as the negative control.

### 2.11 Preparation of mCherry‐CSS‐BBV@COS and GDH‐gD‐Fc‐CSS‐BBV@COS

COS were dissolved in deionized water to prepare solutions of different concentrations (2–14 mg/mL), and the pH was adjusted to 5.0–6.0. Subsequently, different COS solutions were added dropwise to the mCherry‐CSS‐BBV or GDH‐gD‐Fc‐CSS‐BBV solutions with gentle stirring to facilitate complex formation. The mixture was incubated at 4°C for 30 min to stabilize the complexes. Non‐adsorbed COS were removed using centrifugation with a 100 kDa filter, yielding mCherry‐CSS‐BBV@COS and GDH‐gD‐Fc‐CSS‐BBV@COS. The zeta potential and surface morphology of BBVs were measured before and after COS encapsulation.

The gD‐Fc exposure on the surface of GDH‐gD‐Fc‐CSS‐BBV@COS was detected by ELISA. A gD monoclonal antibody (capture antibody, stored in our laboratory) was coated onto the ELISA plate. Subsequently, PBS, CSS‐BBV, COS, GDH‐gD‐Fc‐CSS‐BBV and GDH‐gD‐Fc‐CSS‐BBV@COS samples were added separately, with gD‐Fc as the control (all gD‑Fc–containing groups normalized to the same quantity). Next, the HRP‐labelled gD monoclonal antibody (detection antibody, stored in our laboratory) was introduced, followed by the addition of the 3,3′,5,5′‐tetramethylbenzidine (TMB) substrate to initiate the colour development reaction. Quantitative analysis was performed by measuring OD_450_.

The commercial SGF and SIF were preheated to 37°C. Subsequently, mCherry‐CSS‐BBV@COS samples were added to SGF and SIF at ratios of 1:10 and 1:1, respectively. Both mixtures were incubated at a constant temperature of 37°C, and samples were collected at various time points within the first hour of incubation. The collected samples were immediately subjected to SDS‐PAGE, and the amounts of simulated protein degradation in SGF and SIF over time were calculated using grayscale value analysis. The intensity of the mCherry‐CSS‐BBV@COS bands was determined using ImageJ software. The band density at 0 min was taken as the initial value (A0), and the protection rate (%) was calculated as (Ai/A0) × 100%, where Ai is band intensity at different time points (where i = 0.25, 2, 30 and 60 min).

### 2.12 Cellular Uptake, Polarization and In Vitro Bone Marrow Dendritic Cell Maturation Assay

GDH‐gD‐Fc‐CSS‐BBV was labelled with lipophilic fluorescent dye DiD before being coated with COS. After a 20 min incubation with 10 µM DiD at 4°C, the labelled GDH‐gD‐Fc‐CSS‐BBV was washed twice with PBS and ultracentrifuged at 150,000 × g for 2 h at 4°C. Then, mCherry‐CSS‐BBV@COS or DiD‐labelled GDH‐gD‐Fc‐CSS‐BBV@COS were added to RAW 264.7 cells cultured in 6‐well plates, and incubated for 24 h. Simultaneously, a free protein stimulation group was set up as the control. Subsequently, the cells were treated with CellMask (1:1000, Invitrogen, USA) and NucBlue Live Cell Stain ReadyProbes (Invitrogen) for 10 min at 37°C. After washing with PBS, the fresh medium was added, and the cells were observed using a laser scanning confocal microscope. To monitor the lysosomal escape of BBVs, LysoTracker Green (1:15,000, Beyotime, China) was used as a lysosomal fluorescence probe at 6 and 12 h post‐treatment with mCherry‐CSS‐BBV or mCherry‐CSS‐BBV@COS.

Flow cytometry was used to evaluate the polarization of RAW 264.7 cells stimulated by BBVs. RAW 264.7 cells in a six‐well plate were treated with PBS, mCherry, mCherry‐CSS‐BBV@COS, gD‐Fc, GDH, GDH‐gD‐Fc‐CSS‐BBV and GDH‐gD‐Fc‐CSS‐BBV@COS, and collected after 24 h. Subsequently, the cells were washed twice with PBS, incubated with FITC‐labelled anti‐mouse CD206 (1:20, 4A Biotech, China) and PE‐labelled anti‐mouse CD86 antibodies (1:100, Tonbo Biosciences, USA) for 30 min at 4°C, and resuspended for flow cytometry analysis following two more PBS washes. Furthermore, qPCR and ELISA were used to detect the relative mRNA transcription levels of cytokines IL‐12, TNF‐α and protein expression level of iNOS in RAW 264.7 cells stimulated by PBS, mCherry, mCherry‐CSS‐BBV@COS, gD‐Fc, GDH, GDH‐gD‐Fc‐CSS‐BBV and GDH‐gD‐Fc‐CSS‐BBV@COS, respectively.

Bone marrow dendritic cells (BMDCs) were aseptically extracted from the tibiae and femora of mice, treated with red blood cell lysis buffer, and cultured in six‐well plates at a density of ∼4 × 10^5^ cells per well. The culture medium consisted of RPMI‐1640 supplemented with 10% foetal bovine serum, 100 U/mL penicillin G sodium, 100 µg/mL streptomycin, 20 ng/mL IL‐4, and 20 ng/mL GM‐CSF. The medium was replaced every 2 days, and the cells were cultured until Day 6. To assess the maturation state of the cells, immature BMDCs were treated with PBS, gD‐Fc, GDH, GDH‐gD‐Fc‐CSS‐BBV and GDH‐gD‐Fc‐CSS‐BBV@COS, cultured for 24 h, and then stained with APC‐conjugated anti‐mouse CD11c (1:20, Multisciences), FITC‐conjugated anti‐mouse CD80 (1:20, Multisciences), and PE‐conjugated anti‐mouse CD86 (1:1000, Tonbo Biosciences) to evaluate the maturity of BMDCs.

### 2.13 Mouse Vaccination and Challenge

All animal experiments were conducted in compliance with the Guide for the Care and Use of Laboratory Animals, established by the Ministry of Science and Technology of the People's Republic of China. The experiments were carried out under the supervision of the Committee on the Ethics of Animal Experiments (approval No. 240903‐04‐GR) at the Harbin Veterinary Research Institute, part of the Chinese Academy of Agricultural Sciences.

Six‐week‐old female BALB/c mice were randomly grouped and acclimatized for 1 week prior to immunization. The intramuscular immunization groups received PBS, mCherry, mCherry‐CSS‐BBV, mCherry‐CSS‐BBV@COS, gD‐Fc, GDH, GDH+gD‐Fc+BBV+COS and GDH‐gD‐Fc‐CSS‐BBV@COS, with an immunization dose of 30 µg of protein. Naked proteins (mCherry, gD‐Fc, and GDH) were emulsified with the ISA201 adjuvant at a 1:1 volume ratio. Additionally, commercial inactivated vaccines against PRV (Keqian Bio Co. Ltd., Wuhan, China; PRV‐inactivated) and *S. suis* (Keqian Bio Co., Ltd.; SS‐inactivated) were used for intramuscular immunization. All groups with the exception of the PBS, PRV‐inactivated‐, and SS‐inactivated groups, were immunized orally at the same doses. Each group of mice received two immunizations with a 2‐week interval, with the first immunization day designated as Day 0.

Within 24 h post immunization (hpi), the dynamic distribution of mCherry fluorescent protein in mice was monitored using a NightOWL LB 983 in vivo Imaging System (Berthold Technologies GmbH & Co. KG, Germany) configured with an excitation wavelength of 500 nm and an emission wavelength of 600 nm. Subsequently, the major organs were collected and imaged using the same instrument to capture fluorescence signals. Simultaneously, gastrointestinal tissues were collected within 12 hpi for fluorescence imaging to estimate the rate of mCherry degradation in different treatment groups. All data were collected using IndiGO2 software and statistically analysed using GraphPad Prism 9.5.1. The results are presented as bar graphs.

At 35 days post initial immunization, six mice from each group were randomly chosen and challenged with 50 × LD_50_ of *S. suis* 2 and 10 × LD_50_ of PRV strain HLJ8. The health status of the mice was monitored, and survival rates were recorded and statistically analysed over a 2‐week period post challenge. At 14 days post challenge, the remaining animals were humanely euthanized, and the major organs (brain and lung for PRV‐challenged mice; lung, liver, spleen and kidney for *S. suis* 2‐challenged mice) were excised and fixed with 4% paraformaldehyde for haematoxylin and eosin (H&E) staining.

### 2.14 Determination of Antibody Levels and Cytokines in Mice

Before and after immunization, blood and vaginal discharge of the mice were collected to evaluate antibody levels and cytokine concentrations. Blood samples were allowed to stand at 37°C for 1 h and then stored overnight at 4°C for serum extraction. ELISA was used to quantify the levels of IgG and sIgA against SC‐LPS, SC outer membrane proteins (OMPs) and specific proteins (mCherry, GDH and gD). mCherry, GDH, gD, LPS and OMPs derived from SC bacteria, were used to coat 96‐well plates at a concentration of 5 µg/mL. After washing and blocking, the 50‐fold diluted serum and vaginal discharge samples were added and incubated. Subsequently, enzyme‐labelled antibodies against IgG (1:10,000, Invitrogen), IgG1 (1:8000, Southern Biotech), IgG2a (1:8000, Southern Biotech) and sIgA (1:8000, Southern Biotech) were introduced, followed by the addition of the 3,3′,5,5′‐tetramethylbenzidine substrate to measure the absorbance for IgG, IgG1, IgG2a and sIgA quantification. Additionally, IL‐4 and IFN‐γ cytokine concentrations in serum were measured using ELISA kits. The sample sizes were six for the antibodies and three for the cytokines. For the serum neutralization assay, 100 µL of Vero E6 cells (10^5^ cells/mL) were seeded into a 96‐well culture plate and cultured at 37°C in the atmosphere of 95% air and 5% CO_2_ until 80%–90% confluence. Serum samples were inactivated at 56°C for 30 min, followed by serial two‐fold dilutions starting at 1:2. Then, diluted sera were mixed with an equal volume (50 µL) of 200 TCID_50_ PRV and incubated at 37°C in the atmosphere of 95% air and 5% CO_2_ for 1 h. Subsequently, 100 µL of the mixture was inoculated into Vero E6 cells in a 96‐well plate to monitor the appearance of specific cytopathic effect. Controls included pure viruses, diluted serum and blank cells. All serum samples were tested in quadruplicate. The highest serum dilution that resulted in 50% inhibition of PRV infection was recorded as the serum neutralization titre, which was calculated using the Reed–Muench method.

### 2.15 Determination of Splenic T‐Lymphocyte Subsets, T‐Cell Proliferation and Cytokines

One week after the second immunization, three mice from each group were randomly selected and euthanized. Spleens were collected, and splenic lymphocytes were isolated using the Mouse Spleen Lymphocyte Isolation Kit. Subsequently, the isolated splenic lymphocytes were incubated with either PerCP‐Cyanine5.5‐conjugated anti‐CD3, FITC‐conjugated anti‐CD4, or PE‐conjugated anti‐CD8 in a dark environment at 4°C for 30 min. The cells were then analysed using flow cytometry with appropriate gating controls established for the fluorescent antibodies. Following the selection of CD3^+^ cells, the percentages of CD4^+^ (CD3^+^CD4^+^) and CD8^+^ T cells (CD3^+^CD8^+^) were assessed. Data analysis was performed using the FlowJo version 10.8.1 software.

For the analysis of T cell proliferation, splenic lymphocyte suspension was adjusted to 2 × 10^7^ cells/mL and inoculated into a 96‐well cell culture plate at 100 µL per well. The cells were subsequently stimulated in vitro with respective antigens for 72 h. Then, 10 µL of the MTT solution was added to each well. After additional incubation for 2 h, the OD_450_ of each well was measured using a standard instrument. Data are presented as a proliferation index calculated using the following formula:

$$\begin{array}{ccc} & & \mathrm{Proliferation}\;\mathrm{index}\\ & & =\mathrm{stimulated}\;\mathrm{group}\;\mathrm{OD}/\mathrm{unstimulated}\;\mathrm{group}\;\mathrm{OD}. \end{array}$$

The supernatants from stimulated splenic lymphocytes were collected after 72 h of incubation for cytokine expression analysis. The appropriate ELISA kits were used to quantify IL‐4 and IFN‐γ cytokine concentrations in the supernatants.

### 2.16 Complement‐Mediated Bactericidal Assay

The bactericidal activity of the serum from mice immunized with mCherry‐CSS‐BBV@COS at 35 dpi was evaluated. The SC bacterial suspension was diluted to 1 × 10^3^ CFU/mL, and both immune and untreated sera were heat‐inactivated at 56°C. Serum samples were serially diluted two‐fold in 96‐well plates and mixed with rabbit complement and bacterial suspensions. The control group contained bacterial mixtures without complement or serum. After incubation at 37°C for 1 h, the mixtures were plated on the LB medium, and colonies were counted after 24 h. The bactericidal rate was calculated using the following formula: bactericidal rate = [1−(average CFU of the test control/the negative control)] × 100%.

### 2.17 Data Analysis

Data analyses were performed using GraphPad Prism version 9.5.1 (GraphPad Software, California, USA). Data are presented as the mean ± standard deviation of the mean (SD) or standard error of mean (SEM). The data points in the statistical graph represent the n values (*n* = 3 or 6). The significance of differences between numerical values was assessed using one‐way or two‐way analyses of variance (ANOVA) followed by the Tukey multiple comparisons test, if appropriate. Two‐way ANOVA was used to compare the levels of IgG against GDH, gD, LPS, OMP, TNF‐α and IL‐6 in the serum of immunized mice determined using ELISA. Other comparisons were performed using one‐way ANOVA. The mean of each group was arranged from the largest to the smallest, and the largest mean was marked with the letter a. Values marked by distinct letters are significantly different (*p* ˂ 0.05). Values marked by the same letter or letter colour are not statistically different.

## 3 Results

### 3.1 Lipid A‐Modified SC Significantly Reduces the Inflammatory Response

**FIGURE 2:**
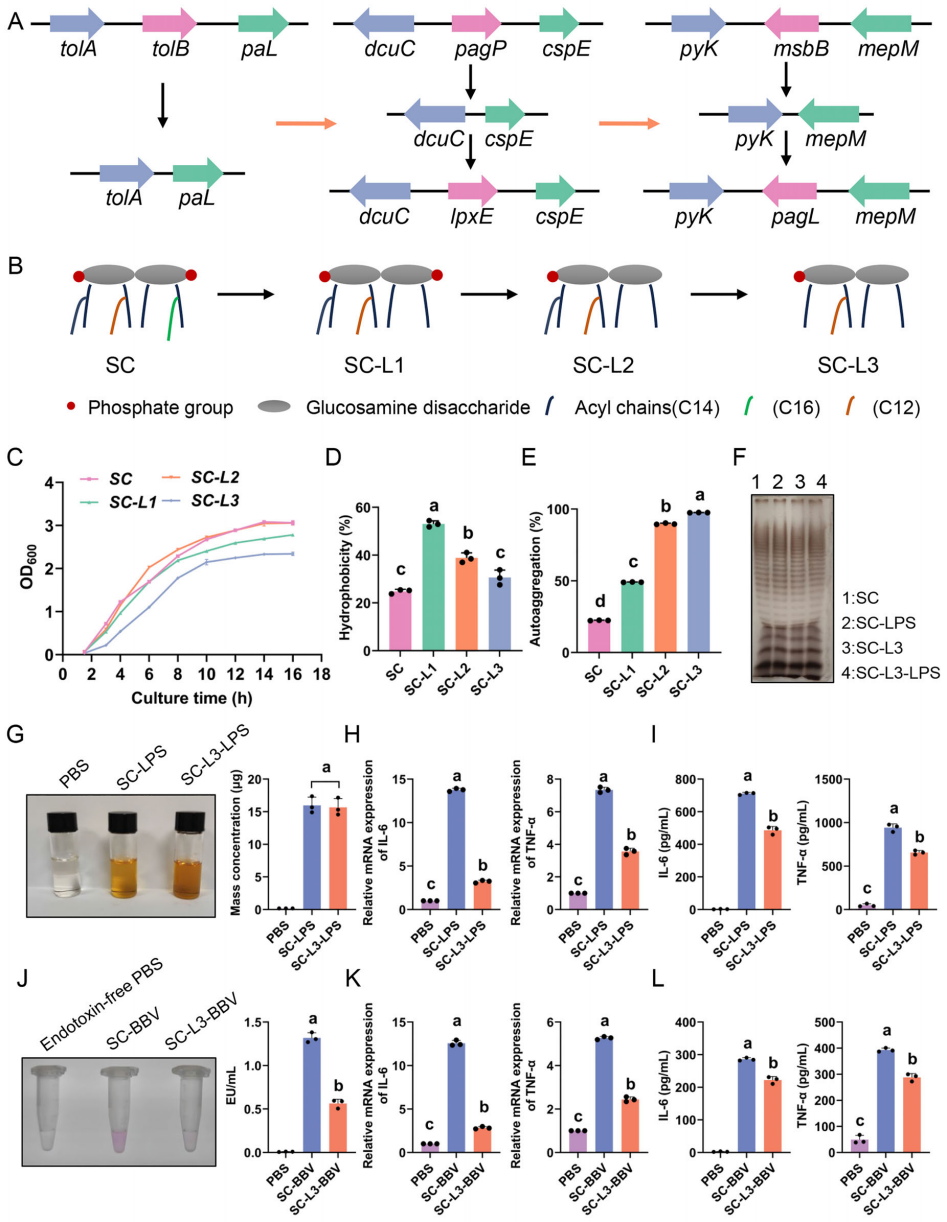
Lipid A‐modified *Salmonella choleraesuis* strain (SC)‐L3 significantly reduces the inflammatory response. (A) Schematic diagram of the chromosomal structure of SC ∆*tolB*∆*pagP*::*lpxE* and ∆*msbB*::*pagL*. (B) Schematic structure of lipid A illustrating the number of phosphate groups and acyl chains in lipid A. Theoretically, lipid A of the SC‐L3 mutant strain has a penta‐acyl monophosphate structure. (C) Comparison of growth curves of SC and its derivative mutant strains. (D, E) Hydrophobicity (D) and autoaggregation ability (E) of SC and its derivative strains. (F) Comparison of LPS phenotypes of SC and SC‐L3 using the silver staining technique. (G) Quantification of LPS in SC and SC‐L3 using the aldol condensation reaction. (H, I) Expression levels of pro‐inflammatory cytokines IL‐6 and TNF‐α following stimulation of RAW 264.7 cells with 1 µg/mL of LPS. qPCR (H) and ELISA (I) were used for the detection of mRNA and protein levels, respectively. (J) Quantification and statistical analysis of endotoxin activity in SC‐biomimetic vesicles (BBV) and SC‐L3‐BBV using the limulus amebocyte lysate endotoxin assay kit. (K, L) Expression levels of pro‐inflammatory cytokines IL‐6 and TNF‐α in RAW 264.7 cells measured after stimulation with SC‐BBV and SC‐L3‐BBV for 12 h. qPCR (K) and ELISA (L) were used for the detection of mRNA and protein levels, respectively.

To reduce endotoxin activity of *S. choleraesuis* strain SC014 (SC), suicide plasmids P1–P5 were used to delete the *tolB*, *msbB* and *pagP* genes and insert the *lpxE* and *pagL* genes of the target strain through allelic exchange and SacB counter‐selection. Figure 2A illustrates the chromosomal structures of the SC‐L1 (Δ*tolB*), SC‐L2 (Δ*tolB*Δ*pagP*::*lpxE*) and SC‐L3 (Δ*tolB*Δ*pagP*Δ*msbB*::*lpxE*::*pagL*) mutants. Primers DtolB‐1F/2R, DpagP‐1F/2R and DmsbB‐1F/2R were used to identify the corresponding mutants. Based on the SC genome, the expected PCR product sizes were approximately 2220, 1555 and 1911 bp. The *tolB*, *pagP* and *msbB* genes have 1293, 573 and 969 bp, respectively, therefore, successful mutations would reduce the PCR product sizes to 927, 982 and 942 bp, respectively. The sizes of the actual PCR products were in accordance with their theoretical sizes (Figure S1A). Furthermore, PCR using specific primers confirmed *lpxE* and *pagL* insertion (Figure S1B, C). These results indicated successful SC deletions and insertions of the target gene.

Lipid A was extracted from both the SC and SC‐L3 strains and subjected to mass spectrometry. Figure 2B illustrates schematic structural changes in lipid A in SC‐L3 from the hepta‐acylated bis‐phosphate to the penta‐acylated monophosphate form. The *msbB* mutation in SC led to the loss of the myristoyl chain (C14:0), resulting in the hexa‐acylated lipid A. Additionally, the introduced *LpxE* gene could remove a phosphate group from lipid A, whereas mutations and insertions in *pagP* and *pagL* eliminated the acyl chains (C16:0). Mass spectrometry results indicated that the maximum peak of lipid A isolated from SC‐L3 was between 1465 and 1665 m/z, which was lower than the 1938 m/z peak observed for the parent SC strain (Figure S2A, B). This reduction in the m/z value corresponded to the loss of two acyl chains and a phosphate group, demonstrating the successful modification of lipid A. The growth dynamics, surface hydrophobicity and autoaggregation properties of SC were assessed before and after the modification. The growth pattern of the SC‐L3 mutant strain was similar to that of the parental strain (Figure 2C). In terms of surface hydrophobicity, SC‐L3 showed no obvious differences from the parent strain; however, its auto‐aggregation capability was markedly enhanced (Figure 2D, E). These modifications facilitated the subsequent purification of BBVs and enhanced their adherence to mucosal surfaces.

LPS is a prototypical endotoxin that promotes the secretion of pro‐inflammatory cytokines TNF‐α and IL‐6 (Shen et al. 2024; Beutler et al. 1986; Liu et al. 2020). Cytokine levels in LPS‐stimulated murine macrophages were measured to assess the pro‐inflammatory effects of SC‐L3. The high purity of LPS extracted from both the SC and SC‐L3 strains was confirmed using silver staining, which revealed a structural phenotype consistent with that of the strains themselves (Figure 2F). Subsequently, the mass concentration of LPS was determined using the phenol‐sulfuric acid method. The colour intensity after the reaction was comparable between the two strains (Figure 2G). Therefore, modifying lipid A did not significantly affect LPS purification, thereby excluding the potential interference of variations in LPS content on in vitro and in vivo inflammatory responses. The mRNA and protein levels of TNF‐α and IL‐6 were quantified using real‐time PCR and ELISA, respectively. The protein level and mRNA expression of TNF‐α and IL‐6 were reduced by SC‐L3‐LPS compared with those in SC‐LPS‐induced RAW 264.7 cells (Figure 2H, I). In the limulus amebocyte lysate chromogenic assay, the absorbance of the red product positively correlated with endotoxin activity of BBVs, expressed in EU/mL. As shown in Figure 2J, SC‐L3‐BBV had a lighter red colour than SC‐BBV. Statistical analysis indicated that endotoxin activity of SC‐L3‐BBV was approximately 0.5 EU/mL and significantly lower than that of SC‐BBV. The protein level and mRNA expression of TNF‐α and IL‐6 were decreased by SC‐L3‐BBV compared with those in the SC‐BBV (Figure 2K, L). Therefore, lipid A‐modified SC‐L3 allowed isolation of BBVs with low endotoxin activity, which is crucial for ensuring the safety of BBVs as vaccine delivery systems.

### 3.2 ClyA Facilitates the Display of Heterologous Proteins to Bacterial and BBV Surfaces

To evaluate the ability of ClyA to facilitate the display of heterologous proteins on the surface of SC‐L3 and BBVs derived from it, mCherry, a fluorescent protein, was fused to ClyA using three GGGS linkers, creating the SC‐ClyA‐mCherry strain (Figure S3A). A control strain, SC‐mCherry, was constructed to express mCherry in the SC without ClyA. The fusion of mCherry (∼26 kDa) with ClyA produced a significantly larger band on the SDS‐PAGE gel (∼60 kDa), corresponding to the expected molecular weight (Figure S3B), thereby confirming the expression of the fusion protein in a soluble form. After IPTG induction, SC‐ClyA‐mCherry and SC‐mCherry strains were incubated with anti‐mCherry monoclonal antibodies and FITC‐conjugated secondary antibodies. The flow cytometry results demonstrated that the SC‐ClyA‐mCherry strain, which incorporated the ClyA fusion, presented a detectable fluorescent signal, whereas no signal was observed in the other groups (Figure S3C). Furthermore, laser confocal microscopy indicated that only the SC‐ClyA‐mCherry strain showed the co‐localization of green and red fluorescence (Figure S3D). Therefore, the mCherry protein was localized on the bacterial surface. Subsequently, the distribution of mCherry in the outer membrane components of the SC‐mCherry and SC‐ClyA‐mCherry strains was investigated. Membrane proteins are typically localized in the detergent phase. In the SC‐ClyA‐mCherry strain, mCherry was predominantly present in the detergent phase, whereas it was mostly located in the aqueous phase in the SC‐mCherry strain (Figure S3E). These observations were corroborated by SDS‐PAGE and Western blotting (Figure S3F). Collectively, these results indicate that ClyA facilitated the surface localization of the target protein in the SC‐L3 strain.

To determine whether the introduction of ClyA enhanced the surface display of mCherry in BBVs without affecting its expression, BBV‐ClyA‐mCherry was purified and characterized. Size‐exclusion chromatography of purified BBV‐ClyA‐mCherry revealed a singular elution peak (Figure S3G). Transmission electron microscopy of BBV‐ClyA‐mCherry revealed vesicle‐like structures with diameters ranging from 50 to 100 nm, validating the efficacy of combining ultra‐high‐pressure homogenization with size‐exclusion chromatography for BBV purification (Figure S3H). The NanoZS90 test results indicated that the size distribution and zeta potential of the BBV‐ClyA‐mCherry were 89.92 ± 1.73 nm (polydispersity index [PDI] = 0.334 ± 0.044) and −19.8 ± 0.5 mV, respectively (Figure S3I, J). The strong negative charge, as suggested by the zeta potential, facilitated the accumulation of cationic substances on the BBV surface. To further investigate the localization of mCherry, BBV‐mCherry and BBV‐ClyA‐mCherry were subjected to treatment with PK. mCherry, which is located on the outer surface of BBV, was degraded by PK (Figure S3K). Therefore, ClyA facilitated presentation of heterologous proteins on the surface of engineered BBVs from the SC‐L3 strain.

### 3.3 Design and Characterization of the CSS‐BBV‐Based Dual Delivery System

**FIGURE 3:**
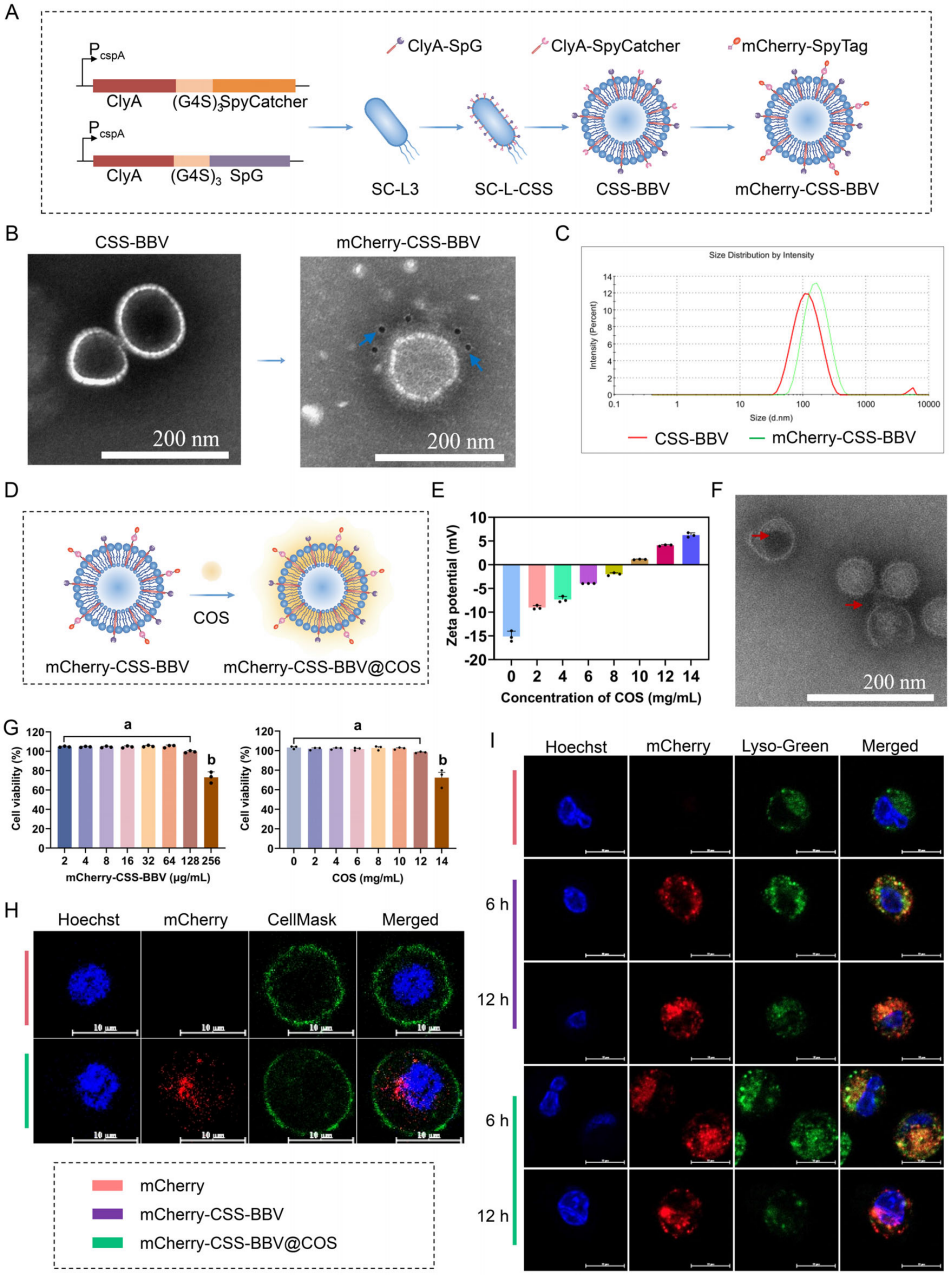
mCherry‐CSS‐BBV@COS escape from lysosomes. (A) Schematic diagram of the CSS‐BBV system for conjugating mCherry‐SpyTag. SpyCatcher and SpG were expressed on the BBV surface as fusion proteins with ClyA. SpyTag‐labelled mCherry binds to CSS‐BBV through isopeptide bond formation between the tag and catcher. (B) Immuno‐EM analysis of CSS‐BBV before and after conjugation with mCherry‐SpyTag. Antibodies labelled with 10‐nm gold particles (blue arrows) were used to identify mCherry‐SpyTag on the surface of CSS‐BBV. (C) Particle size analysis of CSS‐BBV before and after conjugation with mCherry‐SpyTag. The particle diameter and PDI of BBVs were measured using NanoZS90 at room temperature. (D–F) The schematic diagram of COS encapsulating mCherry‐CSS‐BBV (D), zeta potential detection of mCherry‐CSS‐BBV at different COS encapsulation concentrations (E) and transmission electron microscopy (F). Red arrows indicate COS adsorbed on the surface of BBV. (G) Cytotoxicity of mCherry‐CSS‐BBV without (left panel) and with the encapsulation with COS (right panel) detected using the CCK‐8 method. (H, I) Confocal microscopy was used to observe the uptake of mCherry‐CSS‐BBV@COS by RAW264.7 cells (H) (after 24 h of incubation) and lysosomal escape (I) after 6 and 12 h of incubation. Cell nuclei were stained with NucBlue Live Cell Stain ReadyProbes (Hoechst), cell membranes were stained with CellMask (CellMask), and lysosomes were labelled with Lyso‐Tracker Green (Lyso‐Green). Scale bar, 10 µm.

To conjugate various antigens onto the surface of BBVs from the SC‐L3 strain, a dual‐antigen plug‐and‐play system composed of the SpyCatcher/SpyTag and SpG/Fc pairs was used. Specifically, SpyCatcher and SpG were expressed on the BBV surface as ClyA fusion proteins (CSS‐BBV), thus enabling SpyTag and Fc‐fused antigens to be rapidly displayed on CSS‐BBV by site‐specific conjugation. The mCherry‐SpyTag model protein was used to assess the antigen‐displaying ability of CSS‐BBV and its potential for in vivo antigen delivery. The construction strategy for mCherry‐CSS‐BBV is illustrated in Figure 3A. SDS‐PAGE indicated that SpyCatcher and SpG were expressed as fusion proteins with ClyA in the CSS‐BBV. Effective separation of CSS‐BBV from other impurities was achieved using molecular sieve chromatography (Figure S4A). Additionally, the mCherry‐SpyTag was expressed in *E. coli* Decreased Endotoxic BL21 (BL21‐DeE) cells and purified using a HisTrap Nickel column; its purity was confirmed using SDS‐PAGE (Figure S4B). After conjugation of mCherry‐SpyTag with CSS‐BBV, immunoelectron microscopy (immuno‐EM) analysis showed that the surface of mCherry‐CSS‐BBV displayed gold nanoparticles, whereas the non‐coupled CSS‐BBV did not (Figure 3B). NanoZS90 analysis revealed that the average diameters of CSS‐BBV and mCherry‐CSS‐BBV were approximately 93.86 ± 0.88 nm (PDI = 0.292 ± 0.007) and 132.2 ± 3.47 nm (PDI = 0.247 ± 0.019), respectively (Figure 3C), indicating the effective conjugation of mCherry‐SpyTag with CSS‐BBV. Western blotting indicated that the coupling efficiency of mCherry‐SpyTag initially increased and then decreased as the CSS‐BBV concentration increased. The highest coupling efficiencies of 58.05 ± 1.01% and 67.49 ± 0.63% were observed at CSS‐BBV concentrations of 1 and 3.5 mg/mL, respectively (Figure S5A, B). Furthermore, nano‐flow cytometry analysis of the samples under the same binding conditions revealed that CSS‐BBV with fluorescent signals yielded coupling efficiencies of 57.7% and 63.3%, respectively (Figure S5C). Therefore, the CSS‐BBV‐based dual‐antigen delivery system showed high efficiency in binding heterologous antigens.

### 3.4 mCherry‐CSS‐BBV@COS Escape From Lysosomes

To achieve oral immunization with mCherry‐CSS‐BBV, COS were used for coating modification, yielding mCherry‐CSS‐BBV@COS (Figure 3D). Theoretically, the zeta potential of mCherry‐CSS‐BBV@COS increased because of COS adsorption. A dose‐dependent increase in zeta potential was observed with increasing COS concentration, rising from −15.1 ± 1.1 mV to +6.3 ± 0.5 mV at a concentration of 14 mg/mL (Figure 3E). TEM observations revealed that black deposits of COS were located around the surface of the BBVs (Figure 3F). These results provide direct evidence for the surface coating modification.

BBVs cytotoxicity is a critical factor in antigen delivery systems. mCherry‐CSS‐BBV caused no significant in vitro cytotoxicity in RAW264.7 cells at a concentration of 128 µg/mL (Figure 3G). Moreover, with such a biocompatible COS coating, mCherry‐CSS‐BBV@COS showed no significant in vitro cytotoxicity in RAW264.7 cells even at a relatively high concentration of 12 mg/mL. The cellular uptake of antigens is a crucial step in facilitating the initial immune response. To investigate the cellular uptake of BBVs, mCherry‐CSS‐BBV@COS was incubated with RAW264.7 cells. Red fluorescence was observed in the cytoplasm under a confocal laser scanning microscope (Figure 3H), suggesting that mCherry‐CSS‐BBV@COS had been taken up by RAW264.7 cells. The lysosomal escape of mCherry‐CSS‐BBV and mCherry‐CSS‐BBV@COS was investigated using the LysoTracker Green lysosomal fluorescent probe. Within 6 h, mCherry‐CSS‐BBV and mCherry‐CSS‐BBV@COS were captured by the lysosomes, as evidenced by the substantial orange fluorescence from the co‐localization of green and red fluorescence (Figure 3I). However, the co‐localization was transient and significantly diminished at 12 h after treatment (Figure 3I), indicating that mCherry‐CSS‐BBV and mCherry‐CSS‐BBV@COS successfully escaped from the lysosomes.

M1 polarization is essential for immune activation and antigen presentation. The differences in the ability of mCherry and mCherry‐CSS‐BBV@COS to promote M1 polarization in RAW264.7 cells were identified using flow cytometry. Compared with the effects of free mCherry, expression levels of the M1 macrophage‐specific marker CD86 were significantly higher, whereas expression levels of the M2 macrophage‐specific marker CD206 were significantly lower in the mCherry‐CSS‐BBV@COS stimulation group (Figure S6A, B). Additionally, the M1/M2 ratio in the mCherry‐CSS‐BBV@COS‐stimulated group was significantly higher than that in the other two groups (Figure S6B). Furthermore, the mRNA expression levels of IL‐12 and TNF‐α, along with the protein expression level of iNOS in RAW264.7 cells stimulated by mCherry and mCherry‐CSS‐BBV@COS, were assessed. Compared to the effects of free mCherry, the expression levels of IL‐12 (Figure S6C), TNF‐α (Figure S6D) and iNOS (Figure S6E) were significantly elevated in the mCherry‐CSS‐BBV@COS stimulation group. Therefore, the mCherry‐CSS‐BBV@COS complex could be effectively internalized by macrophages, facilitating lysosomal escape and promoting M1 macrophage polarization.

### 3.5 COS Improves mCherry‐CSS‐BBV Resistance to the Gastrointestinal Fluid Environment

**FIGURE 4:**
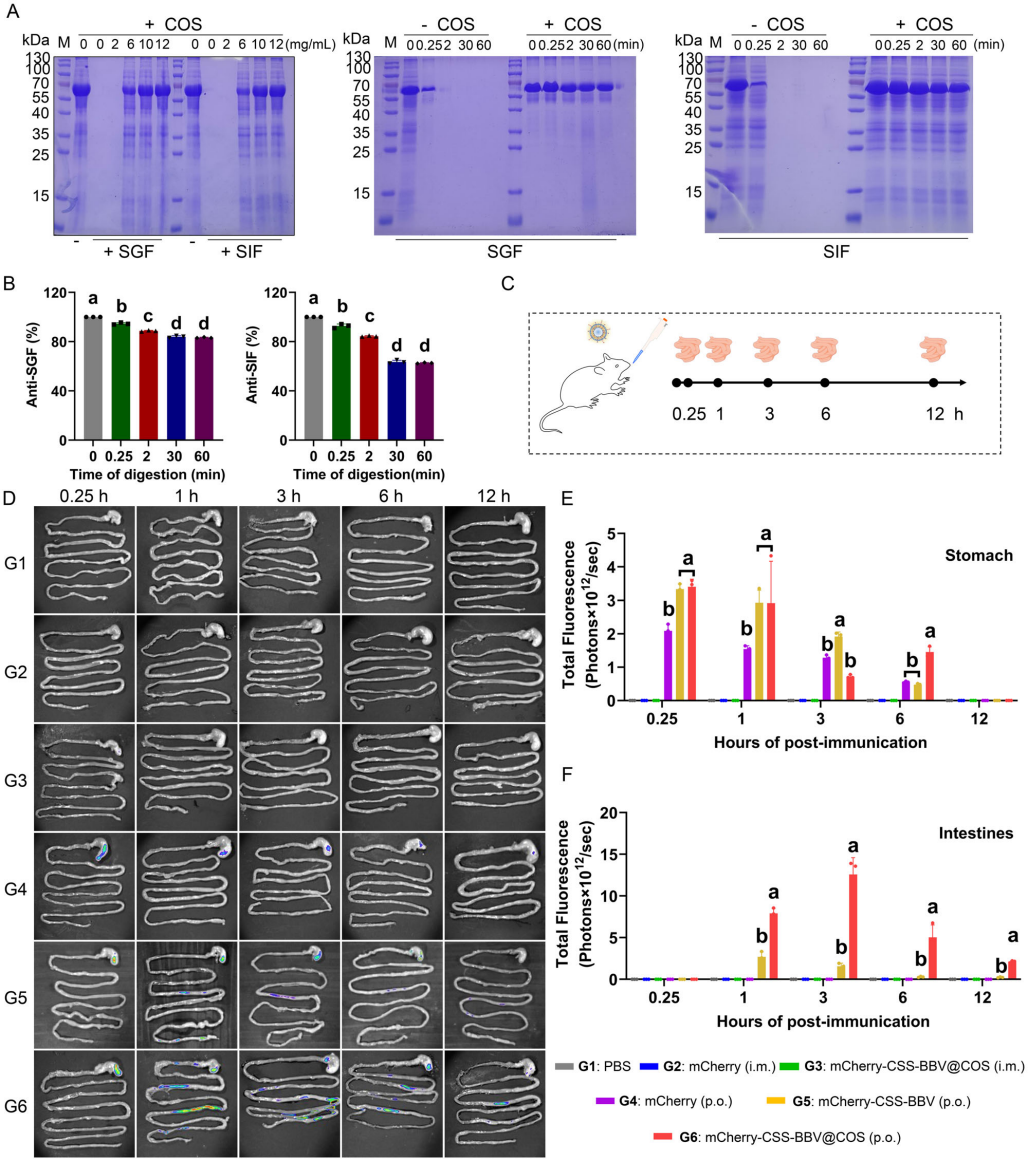
Resistance of mCherry‐CSS‐BBV@COS to the destructive environment of gastrointestinal fluids. (A) SDS‐PAGE gels illustrating concentration‐dependent effects of COS encapsulation on mCherry‐CSS‐BBV (left panel) and resistance of mCherry‐CSS‐BBV to the digestion in the simulated gastric fluid (SGF) and simulated intestinal fluid (SIF) at different time points (central and right panels). (B) Statistical analysis of data illustrated in (A). The Y‐axis shows percentage of undigested mCherry‐CSS‐BBV. (C) Schematic diagram of the in vivo imaging system used to detect the dynamic changes in mCherry levels in gastrointestinal tissues within 12 h post‐immunization in mice. (D–F) Imaging of the gastrointestinal tract in BALB/c mice after administration of mCherry, mCherry‐CSS‐BBV and mCherry‐CSS‐BBV@COS via different routes. Gastrointestinal tissues from each group of mice were harvested for imaging at 0.25, 1, 3, 6 and 12 h post‐administration (D). Fluorescence intensity was quantified in the stomach (E) and intestines (F).

The stability of mCherry‐CSS‐BBV@COS in the gastrointestinal environment was evaluated using simulated gastrointestinal fluid containing proteolytic enzymes. In the absence of COS, mCherry in CSS‐BBV cells was completely degraded in both SGF and SIF. However, COS provided partial protection against degradation at 6 mg/mL. When the COS concentration increased to 10 mg/mL, mCherry‐CSS‐BBV showed significant resistance to degradation by SGF and SIF for up to 1 h (Figure 4A). As the digestion time increased, antigen resistance gradually diminished and stabilized after 30 min, with protection rates of approximately 83% in the SGF and 63% in the SIF (Figure 4B). To investigate the distribution of mCherry in the digestive tract of mice after immunization, an in vivo imaging system was used to detect specific mCherry fluorescence (Figure S7). Dynamic changes in mCherry fluorescent protein levels in the gastrointestinal tissues of mice were observed within 12 h post‐immunization (Figure 4C). Gastrointestinal tissue imaging revealed that fluorescent signals were exclusively observed in the oral immunization group (Figure 4D). Owing to the physiological function of the pylorus, the fluorescence signals were mainly concentrated in the stomach within 15 min post‐administration and gradually diminished over 12 h (Figure 4D, E). Both the mCherry‐CSS‐BBV and mCherry‐CSS‐BBV@COS orally immunized group, fluorescent aggregation appeared in the intestines of mice at 1 h after administration. Notably, the fluorescence signal in the mCherry‐CSS‐BBV@COS group was significantly stronger than that in the mCherry‐CSS‐BBV group. However, no fluorescence signal was detected in the intestines of mice that were orally administered free mCherry (Figure 4D, F). Collectively, these results indicate that COS effectively protected mCherry‐CSS‐BBVs from degradation by gastric fluids and facilitated their delivery to the intestines.

### 3.6 Significantly Enhanced In Vivo Delivery Efficiency of mCherry‐CSS‐BBV@COS

**FIGURE 5:**
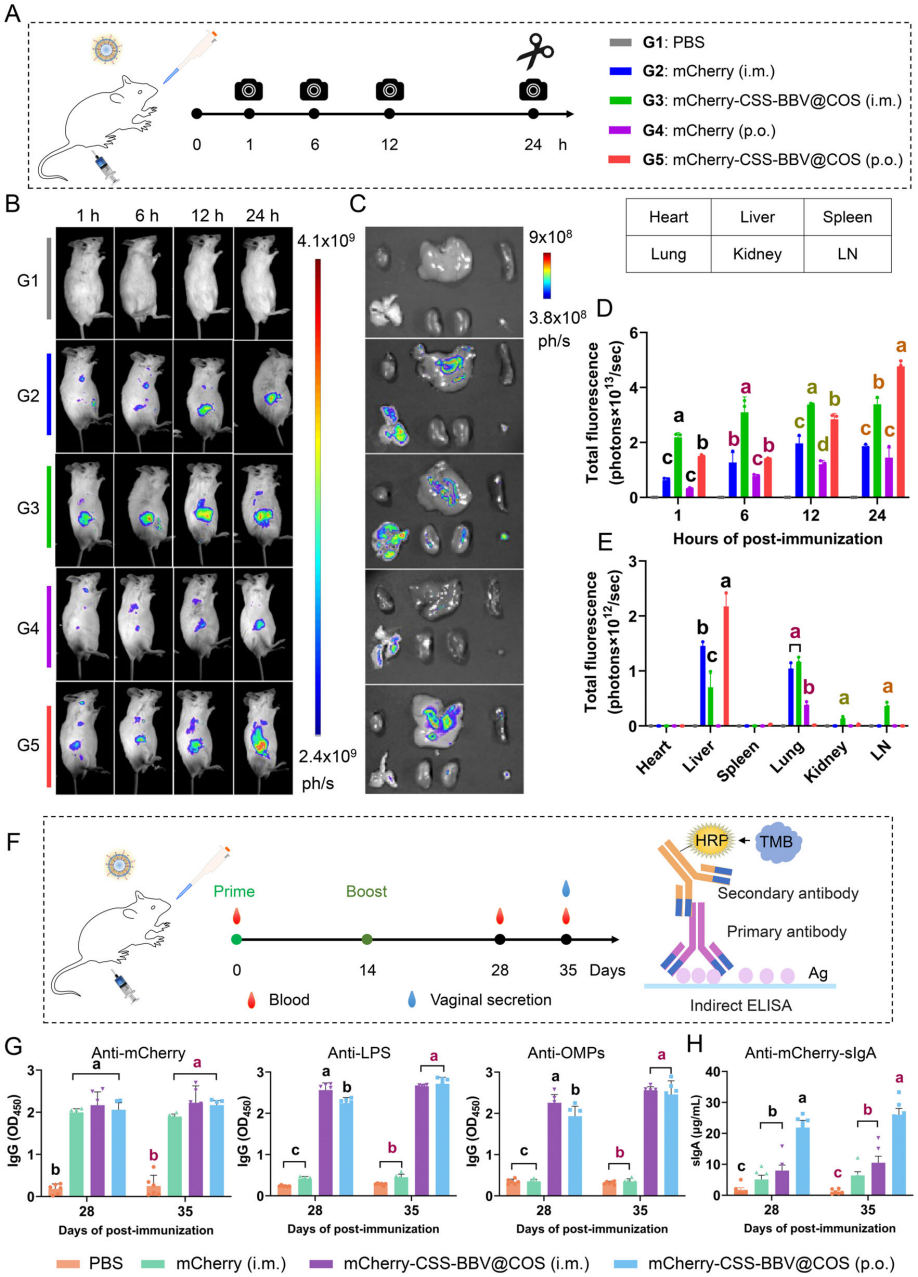
Oral administration maximizes in vivo delivery of mCherry‐CSS‐BBV@COS and induces specific antibody responses. (A) Schematic diagram of in vivo and ex vivo fluorescence imaging of BALB/c mice at different time intervals after mCherry‐CSS‐BBV@COS administration via various routes. (B) Examples of in vivo fluorescence scans performed within 24 h after oral or intramuscular administration of mCherry (30 µg) or mCherry‐CSS‐BBV@COS (containing 30 µg mCherry) to mice. (C) Examples of ex vivo imaging of different organs performed 24 h after administration. (D, E) Summary graphs of experiments in vivo (D) and ex vivo (E). (F) Scheme of the immunization experiment. The mice were immunized with 30 µg of vaccine (mCherry content) via intramuscular injection and oral administration on Day 0, followed by a booster immunization on Day 14. Serum levels of specific IgG and sIgA were detected using indirect ELISA. (G) Serum levels of IgG against mCherry, LPS and OMPs. Data are presented as OD_450_ values. (H) Levels of sIgA against mCherry in vaginal secretions. Data are presented in µg/mL.

To investigate the dynamic distribution of mCherry‐CSS‐BBV@COS, in vivo fluorescence imaging was performed at various time points following oral or intramuscular injections (Figure 5A). In vivo imaging revealed fluorescent signals in all immunized mice containing mCherry, confirming the specific imaging capability of mCherry (Figure 5B). Fluorescence signals in the oral and intramuscular immunization groups originated from the oral digestive tract and leg muscles, respectively, and gradually accumulated in specific areas over time (Figure 5B). Fluorescence intensity in all CSS‐BBV‐treated groups was higher than that in the free mCherry group (Figure 5D), indicating that CSS‐BBV enhanced the efficiency of mCherry tissue delivery. This may be related to the delayed in vivo clearance of CSS‐BBV. Moreover, although the fluorescence value in the mCherry‐CSS‐BBV@COS oral group was lower than that in the intramuscular injection group within the first 12 h, it significantly increased by the 24 h time point.

Surveillance of pathogens by lymph nodes (LNs) and the subsequent activation of the immune response are key components of a functional immune system. LN localization is crucial for responses based on DCs and antigen transport to lymph nodes. Therefore, major organs were harvested for ex vivo imaging at 24 h post immunization (hpi). Fluorescent signals were detected in various tissues and organs of the mice, except in the PBS group (Figure 5C). Specifically, fluorescence signals in the free mCherry group primarily localized to the liver and lungs, whereas the mCherry‐CSS‐BBV@COS‐i.m. group showed signals in the spleen, kidneys and inguinal LNs. However, only faint fluorescence signals were observed in the inguinal LNs of the orally administered group (Figure 5C,E), suggesting that mCherry‐CSS‐BBV@COS primarily targeted gut‐associated lymphoid tissues upon oral administration. The highest fluorescence signals in all immunized groups were noted in the liver (Figure 5E), possibly because of its capacity to process and store substances from the blood via the portal vein system. Therefore, the results of these in vivo experiments indicate that CSS‐BBV carriers facilitated antigen delivery to antigen presenting cells in LNs.

### 3.7 mCherry‐CSS‐BBV@COS Immunization Induces Specific Antibody Responses to mCherry and SC in Mice

To further assess the capacity of mCherry‐CSS‐BBV@COS to induce antigen‐specific immune responses in vivo, mice were administered antigens intramuscularly or orally on Day 0, followed by a booster immunization on Day 14 (Figure 5F). The levels of antigen‐specific IgG and sIgA in serum and vaginal secretions were quantified using indirect ELISA (iELISA). Intramuscular and oral administrations of mCherry‐CSS‐BBV@COS led to significantly higher levels of IgG against mCherry, LPS and OMPs compared to those observed in the PBS group, indicating that mCherry‐CSS‐BBV@COS elicited a robust antibody response (Figure 5G). No significant difference in anti‐mCherry IgG levels was observed between the free mCherry and mCherry‐CSS‐BBV@COS groups, suggesting that the adjuvanticity of CSS‐BBV was comparable to that of the commercial adjuvant, ISA201. Additionally, at 5 weeks after oral immunization, mCherry‐CSS‐BBV@COS induced LPS‐ and OMP‐specific IgG levels comparable to those induced by injection immunization. Moreover, mice orally immunized with mCherry‐CSS‐BBV@COS showed significantly higher anti‐mCherry sIgA levels than those in the other groups (Figure 5H). Therefore, oral immunization elicited robust systemic and mucosal immune responses, thereby validating the conclusions drawn from the fluorescence imaging of gastrointestinal tissues. Furthermore, the bactericidal activity of serum mCherry‐CSS‐BBV@COS was evaluated. When diluted at 1:80, the serum still had an exceptionally strong bactericidal activity of approximately 80% against the SC strain (Figure S8). These data show the potential of CSS‐BBV as oral antigen delivery carriers after coating with COS.

### 3.8 Coupling of CSS‐BBV to GDH‐SpyTag and gD‐Fc Double Fusion Proteins

**FIGURE 6:**
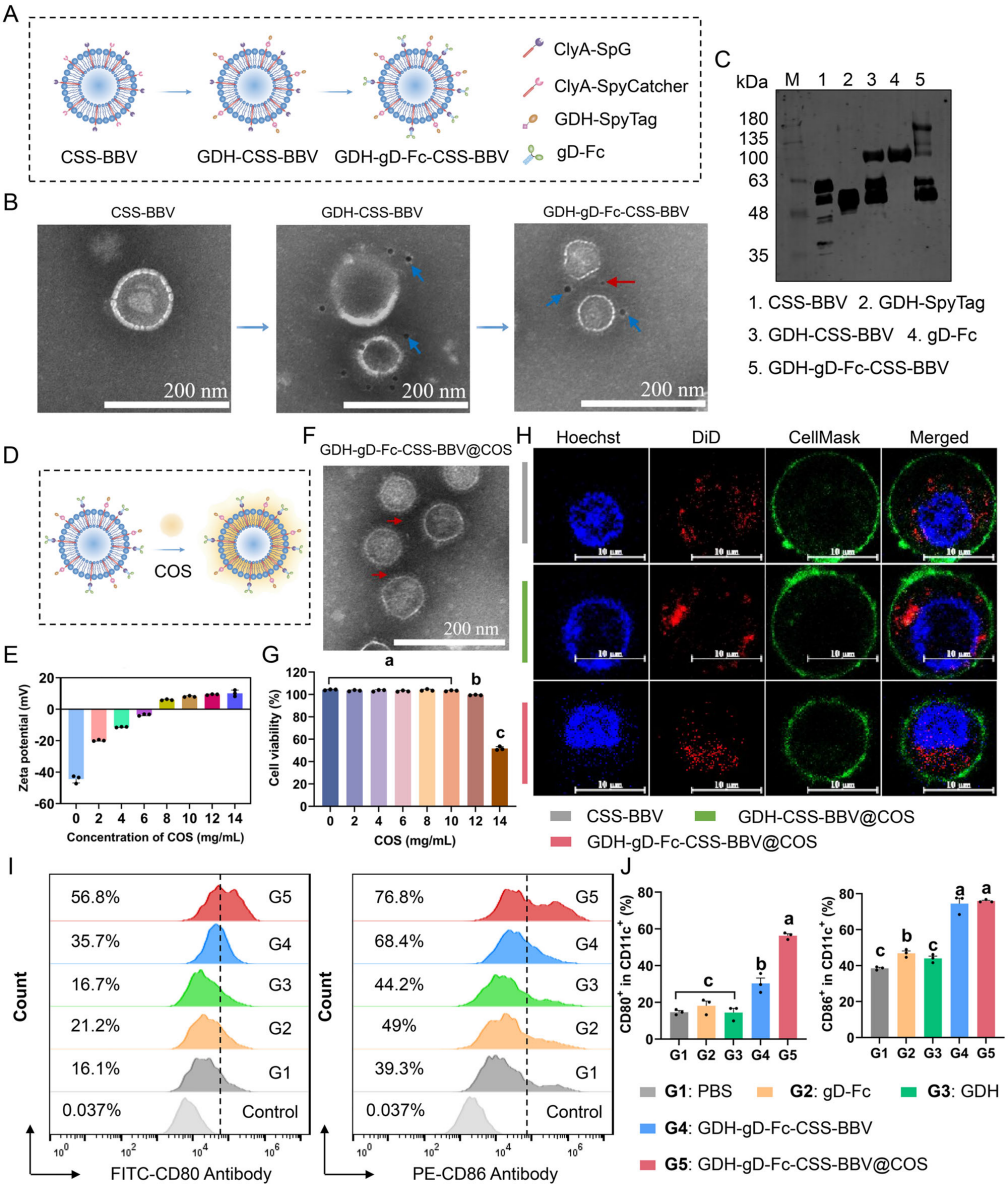
Coupling of CSS‐BBV to GDH‐SpyTag and gD‐Fc double fusion proteins. (A) Schematic diagram of the CSS‐BBV dual fusion protein conjugate. CSS‐BBV was first conjugated with GDH‐SpyTag to obtain GDH‐CSS‐BBV, followed by adsorption of gD‐Fc, ultimately yielding GDH‐gD‐Fc‐CSS‐BBV. (B) Immunoelectron microscopy observation of CSS‐BBV dual antigen conjugation. Antibodies labelled with 10 nm (blue arrows) and 5 nm (red arrows) gold nanoparticles were used to recognize GDH and gD‐Fc on CSS‐BBV, respectively. (C) The formation of CSS‐BBV conjugated dual antigens were confirmed using Western blotting with a monoclonal antibody against His‐tag. (D, E) Schematic diagram of COS‐coated GDH‐gD‐Fc‐CSS‐BBV (D) and zeta potential measurements (E). (F) Transmission electron microscopy observation of COS‐coated GDH‐gD‐Fc‐CSS‐BBV. (G) Cytotoxicity of COS‐encapsulated GDH‐gD‐Fc‐CSS‐BBV. (H) Confocal microscopy observations of the GDH‐gD‐Fc‐CSS‐BBV@COS uptake by RAW264.7 cells after 24 h of incubation. The cell nuclei were stained with Hoechst, cell membranes were stained with CellMask and BBVs were labelled with DiD. Scale bar, 10 µm. (I, J) Flow cytometry of the proportions of CD11c^+^ CD80^+^ and CD11c^+^ CD86^+^ cells (I) 24 h after stimulation with 5 µg/mL gD‐Fc, GDH, CSS‐BBV‐GDH‐gD‐Fc, or CSS‐BBV‐GDH‐gD‐Fc@COS and statistical analysis of the data obtained (J).

These experiments demonstrated that CSS‐BBV could be effectively conjugated with the SpyTag‐fused mCherry model antigen. Next, conjugation efficiency of CSS‐BBV with *S. suis* 2 GDH and PRV gD were investigated. GDH, a protective antigen across multiple serotypes, was labelled with SpyTag (GDH‐SpyTag) to bind to SpyCatcher on CSS‐BBV surface. gD, an essential PRV glycoprotein with high immunogenicity, was fused to the Fc fragment of porcine IgG1 (gD‐Fc) for coupling with SpG on CSS‐BBV surface. The purified GDH‐SpyTag and gD‐Fc fusion proteins showed high purity with a single band of the expected molecular weight (Figure S9A, B). To obtain GDH‐gD‐Fc‐CSS‐BBV, CSS‐BBV was conjugated with GDH‐SpyTag before conjugation with gD‐Fc (Figure 6A). Successful conjugation of GDH‐gD‐Fc‐CSS‐BBV was directly visualized using immuno‐EM (Figure 6B). GDH‐CSS‐BBV displayed 10 nm gold‐labelled particles (blue arrows), and after coupling with gD‐Fc, both 10 and 5 nm gold‐labelled particles (red arrows) were observed on GDH‐gD‐Fc‐CSS‐BBV. Western blotting confirmed the successful conjugation of GDH‐SpyTag and gD‐Fc to CSS‐BBV, with a gradual increase in molecular weight compared with that of CSS‐BBV and GDH‐CSS‐BBV (Figure 6C). The average diameter of GDH‐gD‐Fc‐CSS‐BBV (195.57 ± 1.14 nm, PDI = 0.214 ± 0.187) was larger than those of GDH‐CSS‐BBV (144.87 ± 1.69 nm, PDI = 0.214 ± 0.021) and CSS‐BBV (94.91 ± 0.96 nm, PDI = 0.294 ± 0.003) (Figure S10). The conjugation efficiency was tested by varying the concentration of CSS‐BBV. As the concentration of CSS‐BBV increased, the conjugation efficiency of GDH‐SpyTag reached 55.83 ± 0.05% (Figure S11A, C). Furthermore, GDH‐CSS‐BBV could be conjugated with the fluorescently labelled antibody Fc domain, indicating that SpG sites on CSS‐BBV was still available for further conjugation (Figure S11B). Finally, the maximum efficiency of GDH‐CSS‐BBV binding to gD‐Fc was 69.92% ± 0.02% within a certain concentration range of CSS‐BBV (Figure S11D). Therefore, these results indicate that GDH‐SpyTag and gD‐Fc dual antigens could effectively be conjugated to CSS‐BBV.

### 3.9 GDH‐gD‐Fc‐CSS‐BBV@COS Promote Cell Polarization and Bone Marrow‐Derived DC (BMDC) Maturation In Vitro

After GDH‐gD‐Fc‐CSS‐BBV underwent COS coating (Figure 6D), the zeta potential of BBVs substantially changed from −44.4 ± 2.5 mV to 10.2 ± 1.9 mV (Figure 6E). Transmission electron microscopy revealed dispersed black spots on the surface of GDH‐gD‐Fc‐CSS‐BBV@COS (red arrows in Figure 6F), providing direct evidence of COS adsorption. ELISA assay was utilized to assess the surface exposure of conjugated antigens on COS‐coated BBVs. The control groups (PBS, COS and antigen‐free CSS‐BBV) showed only minimal signals. In contrast, the samples GDH‐gD‐Fc‐CSS‐BBV, GDH‐gD‐Fc‐CSS‐BBV@COS and gD‐Fc exhibited strong and specific signals. Notably, the strongest signal was exhibited by gD‐Fc, followed by GDH‐gD‐Fc‐CSS‐BBV, while the weakest signal was produced by GDH‐gD‐Fc‐CSS‐BBV@COS (Figure S12). these results indicate that gD‐Fc remains partially exposed on the surface after COS encapsulation, thus remaining accessible for recognition by its homologous antibodies. GDH‐gD‐Fc‐CSS‐BBV@COS showed no obvious cytotoxicity at a COS concentration of 10 mg/mL (Figure 6G). Additionally, CSS‐BBV could be internalized by RAW264.7 cells prior to and following antigen binding (Figure 6H). Furthermore, flow cytometry was used to investigate the M1 polarization of RAW264.7 cells induced by GDH‐gD‐Fc‐CSS‐BBV@COS. CD86 was significantly upregulated, whereas CD206 was downregulated in cells treated with GDH‐gD‐Fc‐CSS‐BBV and GDH‐gD‐Fc‐CSS‐BBV@COS compared with expression levels of these markers in other groups (Figure S13A). The GDH‐gD‐Fc‐CSS‐BBV@COS group showed a significantly greater reduction in CD206 levels than the GDH‐gD‐Fc‐CSS‐BBV group. Furthermore, the M1/M2 ratio in the GDH‐gD‐Fc‐CSS‐BBV@COS group was significantly higher than those in the other groups. The mRNA expression levels of IL‐12 and TNF‐α, as well as the protein expression level of iNOS in RAW264.7 cells, were evaluated across all stimulation groups. Compared to the PBS group, no significant differences were observed in the expression of IL‐12, TNF‐α and iNOS between the free gD‐Fc and GDH stimulation groups. However, these expression levels were significantly lower than those in the GDH‐gD‐Fc‐CSS‐BBV and GDH‐gD‐Fc‐CSS‐BBV@COS groups. Notably, the mRNA expression levels of IL‐12 and TNF‐α, along with the protein expression level of iNOS, in the GDH‐gD‐Fc‐CSS‐BBV@COS stimulation group were higher than those in the GDH‐gD‐Fc‐CSS‐BBV group, which may be attributed to the encapsulating effect of COS on BBV (Figure S13B‐D). Therefore, the dual antigens on GDH‐gD‐Fc‐CSS‐BBV@COS continued to promote M1 polarization of macrophages.

To activate T cells, antigen‐presenting cells must express co‐stimulatory molecules on their surface. To further investigate the ability of GDH‐gD‐Fc‐CSS‐BBV@COS to activate T cells, the expression of co‐stimulatory molecules CD80 and CD86 on BMDC surface was detected using flow cytometry. Compared with effects of treatment with free proteins, the proportions of CD80^+^ and CD86^+^ cells were significantly higher in the groups treated with CSS‐BBV (Figure 6I, J). Notably, although GDH‐gD‐Fc‐CSS‐BBV@COS showed similar CD86^+^ cells stimulation capacity to that of GDH‐gD‐Fc‐CSS‐BBV, they significantly enhanced CD80^+^ cells, which may be attributed to the effect of COS. Overall, GDH‐gD‐Fc‐CSS‐BBV@COS effectively induced BMDC maturation in vitro.

### 3.10 Oral Immunization With GDH‐gD‐Fc‐CSS‐BBV@COS Induces a Lower Inflammatory Response in Mice

To study in vivo endotoxin activity of GDH‐gD‐Fc‐CSS‐BBV@COS, the body temperature and levels of pro‐inflammatory cytokines in the immunized mice were monitored at 1, 3, 6 and 12 hpi. Body temperature showed a trend of initially increasing and then decreasing within 12 hpi (Figure S14). Mice immunized orally had a smaller increase in body temperature compared to mice immunized by an intramuscular injection, likely owing to a lower stress response. Similarly, serum pro‐inflammatory cytokine levels initially increased and then decreased, peaking at 6 h (Figure S15). Notably, in all groups immunized with CSS‐BBV, TNF‐α and IL‐6 secretion increased less than those in other groups, which may be related to its activation of innate immunity. Therefore, GDH‐gD‐Fc‐CSS‐BBV@COS showed low endotoxic activity in vivo, with particularly after oral immunization.

### 3.11 GDH‐gD‐Fc‐CSS‐BBV@COS Induce Antigen‐Specific Antibody Responses in Mice

**FIGURE 7:**
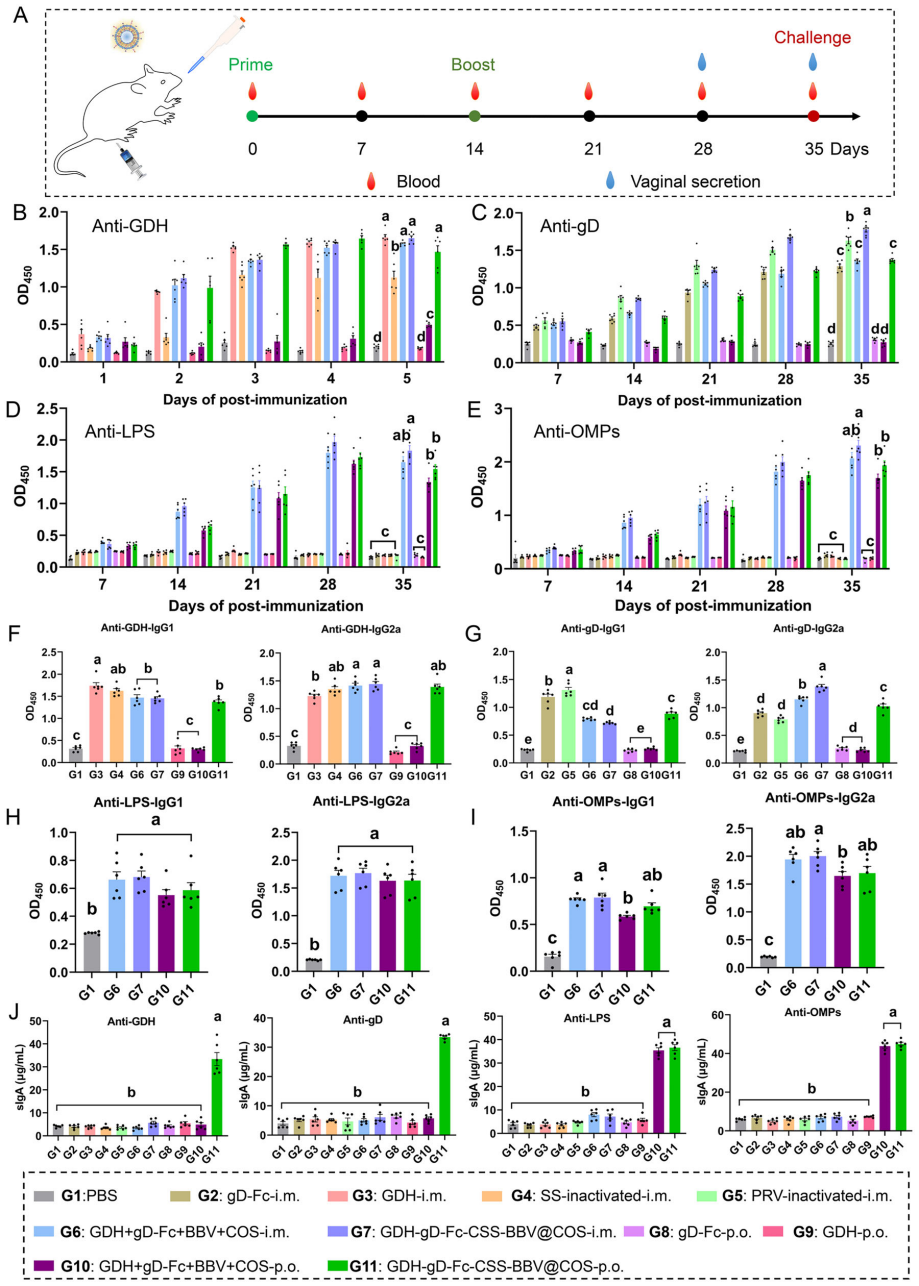
Induction of antigen‐specific antibody responses in mice by GDH‐gD‐Fc‐CSS‐BBV@COS. (A) Schematic diagram of the immunization, blood and vaginal secretion sampling procedures. (B–E) Determination by iELISA of the levels of IgG against GDH (B), gD (C), LPS (D) and OMPs (E) in sera after 50× dilution presented as OD_450_ values. (F–I) Determination by iELISA of the levels of IgG1 and IgG2a against GDH (F), gD (G), LPS (H) and OMPs (I) in sera after 50× dilution presented as OD_450_ values. (J) The levels of sIgA against GDH, gD, LPS and OMP in vaginal secretion determined using iELISA and presented in µg/mL.

To determine whether GDH‐gD‐Fc‐CSS‐BBV@COS could induce specific immune responses, serum IgG levels against GDH, gD, LPS and OMPs were measured using iELISA at 0, 7, 14, 21, 28 and 35 days post‐immunization (dpi) (Figure 7A). All immunization groups except for the PBS, GDH‐p.o. and GDH+gD‐Fc+BBV+COS‐p.o. groups showed high levels of GDH‐specific IgG before the challenge. At 35 dpi, in addition to the GDH+gD‐Fc+BBV+COS‐p.o. group, the GDH‐specific IgG levels in all groups treated with CSS‐BBV were comparable to those in the GDH‐i.m. group injected with the ISA‐201 adjuvant and significantly higher than those in the commercial *S. suis* inactivated vaccine (SS‐inactivated‐i.m.) group (Figure 7B). The trend in the gD‐specific IgG levels was similar. However, the GDH‐gD‐Fc‐CSS‐BBV@COS‐p.o. group showed lower gD‐specific IgG levels compared with those of the GDH‐gD‐Fc‐CSS‐BBV@COS‐i.m. and commercial PRV‐inactivated‐i.m. groups at 35 dpi, but comparable to those in the gD‐Fc‐i.m. group (Figure 7C). Furthermore, all groups that were treated with CSS‐BBV showed elevated levels of LPS‐specific and OMP‐specific IgG, which were significantly higher in the conjugate formulation (GDH‐gD‐Fc‐CSS‐BBV@COS) groups than in the non‐conjugate formulation (GDH+gD‐Fc+BBV+COS) groups (Figure 7D, E). Therefore, the oral delivery of GDH‐gD‐Fc‐CSS‐BBV@COS could effectively induce robust specific humoral immune responses.

In mice, IgG subclasses are closely associated with specific helper T cell (Th cell) responses. Cytokines IL‐4 and IFN‐γ secreted by Th2 and Th1 cells, respectively, promote the differentiation of B cells into plasma cells that produce IgG1 and IgG2a. To clarify the type of Th cell response after immunization, the levels of IgG1 and IgG2a against GDH, gD, LPS and OMPs in mouse sera at 35 dpi were determined using ELISA. Intramuscular and oral immunizations with GDH‐gD‐Fc‐CSS‐BBV@COS stimulated the production of IgG1 and IgG2a specific for GDH, gD, LPS and OMPs in mice (Figure 7F–I). Additionally, oral administration of each CSS‐BBV‐containing formulation induced IgG1 and IgG2a responses against LPS and OMPs. However, the non‐conjugated formulation (GDH+gD‐Fc+BBV+COS) failed to elicit IgG1 and IgG2a responses specific for GDH and gD. Therefore, COS protected heterologous antigens in the GDH‐gD‐Fc‐CSS‐BBV@COS‐p.o. group from degradation by gastrointestinal fluids.

To evaluate the mucosal response induced by the oral administration of GDH‐gD‐Fc‐CSS‐BBV@COS, specific sIgA antibodies against GDH, gD, LPS and OMPs were measured in the vaginal secretions of immunized mice at 35 dpi using iELISA. Among all the immunization groups, only oral immunization with the CSS‐BBV formulation showed elevated levels of specific sIgA against LPS and OMPs (Figure 7J). Furthermore, significantly higher levels of sIgA against both GDH and gD were noted in the GDH‐gD‐Fc‐CSS‐BBV@COS‐p.o. group compared with those in other groups. Overall, oral immunization with GDH‐gD‐Fc‐CSS‐BBV@COS promoted antigen delivery to the intestinal mucosa to produce specific sIgA.

### 3.12 GDH‐gD‐Fc‐CSS‐BBV@COS Induces Cellular Immunity in Mice

**FIGURE 8:**
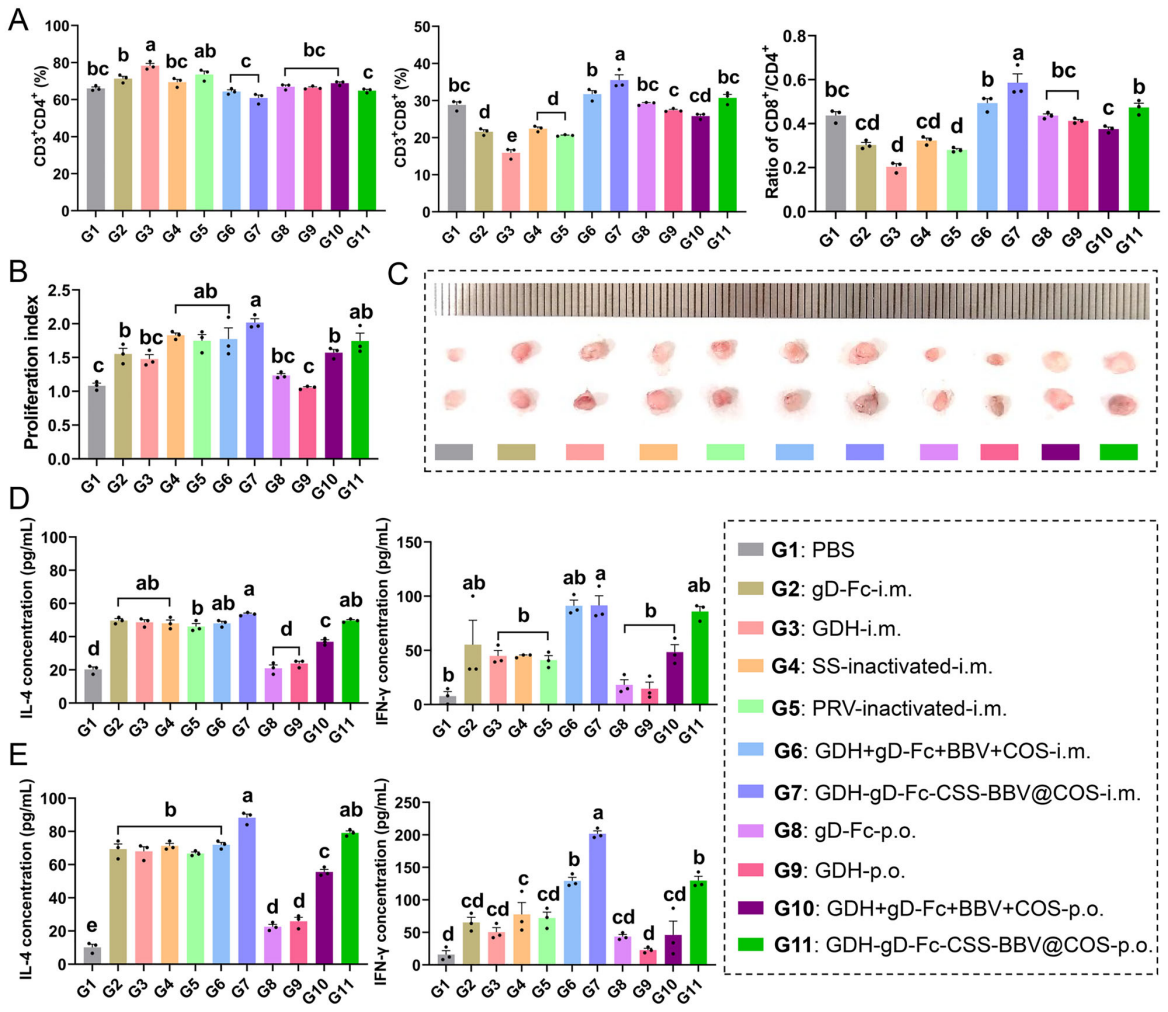
GDH‐gD‐Fc‐CSS‐BBV@COS induce cellular immunity in mice. (A) Flow cytometry analysis of the proportions of the CD3^+^CD8^+^ and CD3^+^CD4^+^ T cells in the spleen. (B) Proliferative capacity in vitro of splenic lymphocytes isolated from mice immunized for 21 days. (C) Inguinal lymph nodes isolated from mice of different immunization groups. (D) IL‐4 and IFN‐γ levels in splenic lymphocyte supernatants measured using ELISA. (E) Serum IL‐4 and IFN‐γ levels measured using ELISA.

The expression of differentiation markers on the surface of splenic lymphocytes was quantified using flow cytometry. Compared with the respective numbers in the PBS group, both intramuscular and oral administrations of GDH‐gD‐Fc‐CSS‐BBV@COS did not significantly change CD4^+^ T cell numbers, whereas CD8^+^ T cell numbers were significantly increased (Figure S16 and Figure 8A). Mice immunized intramuscularly with GDH‐gD‐Fc‐CSS‐BBV@COS showed a significantly higher number of CD8^+^ T cells than those immunized orally. Additionally, oral and intramuscular immunizations with GDH‐gD‐Fc‐CSS‐BBV@COS led to a higher CD8^+^/CD4^+^ T cell ratio compared with that in the PBS group. Therefore, GDH‐gD‐Fc‐CSS‐BBV@COS immunization effectively directed the differentiation pathway of CD8^+^ T cell subsets.

Lymphocytes play a pivotal role in the adaptive immunity. Splenic lymphocytes from immunized mice were isolated and assessed for in vitro proliferation. Lymphocyte proliferation in vitro was promoted in all groups except for the gD‐Fc‐p.o. and GDH‐p.o. groups (Figure 8B). Notably, the conjugate formulation (GDH‐gD‐Fc‐CSS‐BBV@COS) group showed significantly higher proliferation than that of the non‐conjugated GDH+gD‐Fc+BBV+COS group. Although cell proliferation in the orally immunized GDH‐gD‐Fc‐CSS‐BBV@COS group was slightly lower than that in the intramuscularly immunized group, the difference was not statistically significant (Figure 8B). Furthermore, mice administered with formulation containing CSS‐BBV had enlarged inguinal LNs (Figure 8C).

To further evaluate the cellular immune response, IFN‐γ and IL‐4 levels in splenic lymphocyte supernatants were measured. Except for PRV‐inactivated group, the IL‐4 levels were not significantly different between the intramuscular immunization groups (Figure 8D). However, in the oral immunization group, the GDH‐gD‐Fc‐CSS‐BBV@COS‐p.o. group had significantly higher levels of IL‐4 and IFN‐γ than those in the other groups. Furthermore, no significant differences in the IL‐4 and IFN‐γ levels were observed between the non‐conjugated GDH+gD‐Fc+BBV+COS‐i.m., conjugated GDH‐gD‐Fc‐CSS‐BBV@COS‐i.m. and GDH‐gD‐Fc‐CSS‐BBV@COS‐p.o. groups. Additionally, serum levels of IFN‐γ and IL‐4 in immunized mice showed a similar pattern to that in splenic lymphocyte supernatants (Figure 8E). Mice immunized either orally or intramuscularly with GDH‐gD‐Fc‐CSS‐BBV@COS exhibited elevated levels of IL‐4 and IFN‐γ compared with those in other groups. Although IFN‐γ levels in the orally immunized GDH‐gD‐Fc‐CSS‐BBV@COS group were lower than those in the intramuscularly immunized group, IL‐4 levels were comparable. Overall, oral administration of GDH‐gD‐Fc‐CSS‐BBV@COS effectively induced cellular immunity.

### 3.13 GDH‐gD‐Fc‐CSS‐BBV @COS Provides Complete Immunoprotection in Mice

Neutralizing antibodies are crucial indicators of the immunoprotective efficacy of PRV vaccines. Quantification of neutralizing antibodies against PRV in the sera of immunized mice showed that at 35 dpi, the GDH‐gD‐Fc‐CSS‐BBV@COS‐i.m. group had a neutralizing antibody titre of 2^7.14^, comparable to that of the commercial vaccine group (2^7.53^) and significantly higher than that of the free protein (2^5.87^) or GDH+gD‐Fc+BBV+COS‐i.m. (2^6.09^) groups by 2.41‐ and 2.07‐fold, respectively (Figure S17). More importantly, serum neutralizing antibody titre in the GDH‐gD‐Fc‐CSS‐BBV@COS‐p.o. group reached approximately 2^6.32^, which was similar to that in the gD‐Fc‐i.m. group.

**FIGURE 9:**
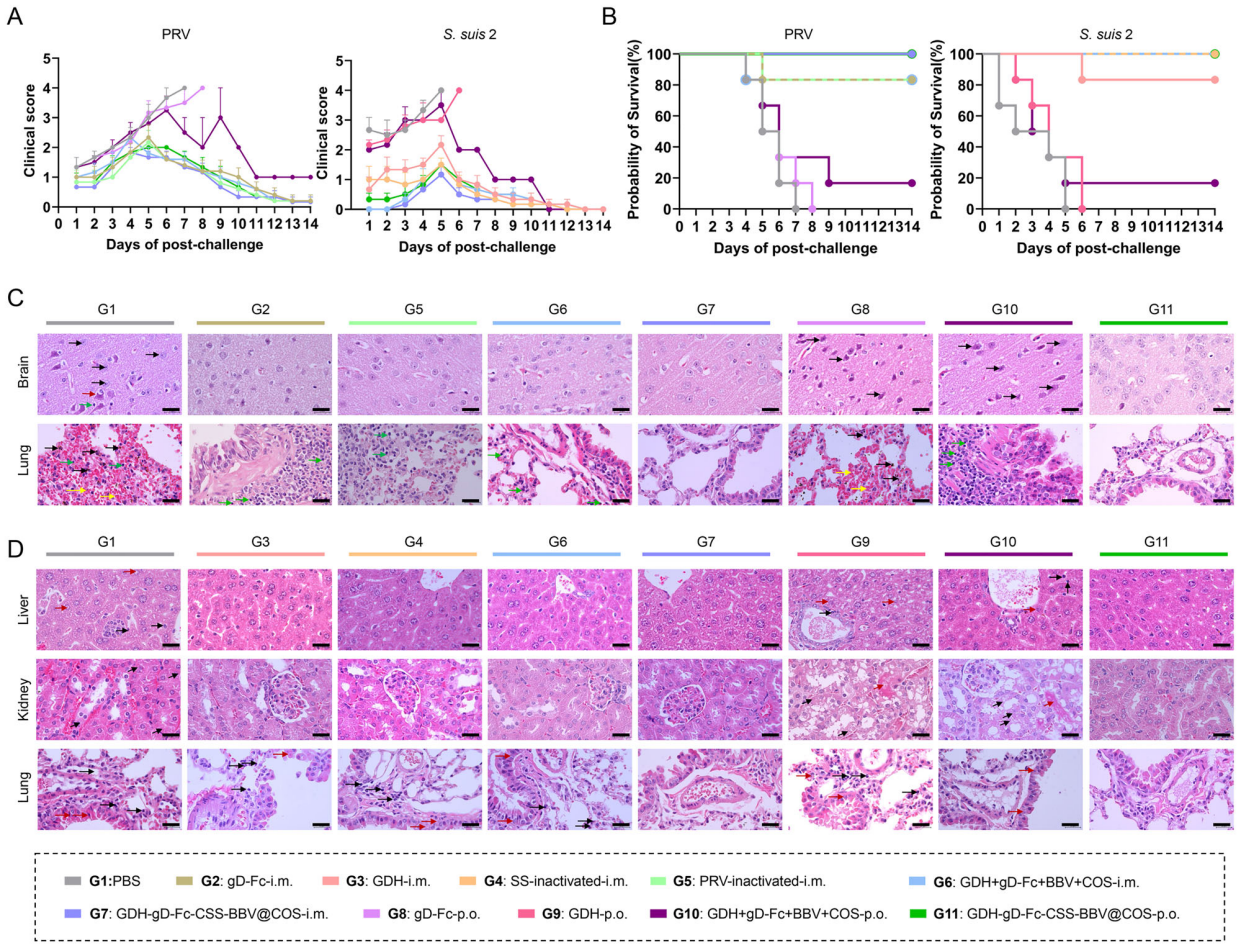
GDH‐gD‐Fc‐CSS‐BBV@COS provide complete immunoprotection in mice. (A) Mouse health status was monitored for 14 days post‐challenge. (B) Mouse survival rates in different immunization groups following challenge with PRV (left panel) or *S. suis* 2 (right panel). (C) Representative images of the histological sections of mouse brain and lung tissues after PRV challenge stained with haematoxylin/eosin. (D) Representative images of the histological sections of mouse liver, kidney and lung tissues after *S. suis* 2 challenge stained with haematoxylin/eosin. Arrows of different colours represent distinct pathological damages. Scale bar, 25 µm.

To evaluate immunoprotective efficacy of GDH‐gD‐Fc‐CSS‐BBV@COS, all mice were challenged with 10 LD_50_ of PRV or 50 LD_50_ of *S. suis* 2 at 35 dpi. The clinical scores indicated that within 14 days, the disease symptoms progressed from mild to severe and then finally returned to baseline, except for fatal cases (Figure 9A). Notably, mice immunized with GDH‐gD‐Fc‐CSS‐BBV@COS displayed the mildest disease symptoms during the observation period. Mouse survival rate was monitored for 14 days after the challenge (Figure 9B). In the context of the PRV challenge, the orally immunized mice receiving GDH‐gD‐Fc‐CSS‐BBV@COS had a 100% survival rate, whereas the gD‐Fc‐p.o. and GDH+gD‐Fc+BBV+COS‐p.o. groups experienced complete mortality and survival rate of 16.6%, respectively. In the intramuscularly immunized mice, the GDH‐gD‐Fc‐CSS‐BBV@COS‐i.m. group showed 100% survival, whereas the PBS group showed complete mortality. The gD‐Fc‐i.m. and commercial PRV‐inactivated‐i.m. groups demonstrated survival rates of 83.3%. For the *S. suis* 2 challenge, the group orally immunized with GDH‐gD‐Fc‐CSS‐BBV@COS also achieved 100% survival, whereas the GDH‐p.o. and GDH+gD‐Fc+BBV+COS‐p.o. groups had survival rates of only 16.6% or less. Among the intramuscularly immunized mice, the GDH‐gD‐Fc‐CSS‐BBV@COS‐i.m., GDH+gD‐Fc+BBV+COS‐i.m. and commercial SS‐inactivated‐i.m. groups demonstrated a 100% survival rate, whereas the GDH‐i.m. group showed 83.3% survival. Overall, these results indicate that oral or intramuscular administration of GDH‐gD‐Fc‐CSS‐BBV@COS offered comprehensive immune protection against lethal doses of PRV and *S. suis* 2.

Histopathological evaluation showed that except in the gD‐Fc‐p.o. and GDH+gD‐Fc+BBV+COS‐p.o. immunization groups, vaccination significantly alleviated or prevented the damage to the major target mouse organs induced by the challenge (Figure 9C, D). In the context of the PRV challenge, only the brain tissue of the PBS, gD‐Fc‐p.o. and GDH+gD‐Fc+BBV+COS‐p.o. groups showed partial neuronal degeneration and necrosis (green arrows), neuronophagia (red arrows) and/or mild glial cell hyperplasia (black arrows) (Figure 9C). Additionally, except for the GDH‐gD‐Fc‐CSS‐BBV@COS immunization groups, which showed no significant pathological damage to the lung tissues, the other immunization groups displayed localized extensive haemorrhage (yellow arrows), hemosiderin deposition (black arrows) and/or accompanying inflammatory cell infiltration (green arrows) (Figure 9C). For the *S. suis* 2 challenge, only the PBS, GDH‐p.o. and GDH+gD‐Fc+BBV+COS‐p.o. groups showed mild inflammatory cell infiltration around the bile ducts in the liver tissue (black arrows), slight hepatocyte degeneration (red arrows) and partial renal tubular epithelial cell degeneration (black arrows) or occasional localized casts (red arrows) (Figure 9D). Additionally, except for the GDH‐gD‐Fc‐CSS‐BBV@COS immunization groups, which showed no significant pathological damage to the lung tissue, other immunization groups showed slight degeneration and necrosis of bronchiolar epithelial cells (red arrows) and/or localized mild inflammatory cell infiltration (black arrows) (Figure 9D).

## 4 Discussion

In the present study, a novel oral dual‐antigen subunit vaccine platform based on genetically engineered BBVs was developed. This platform addresses the key limitations of the traditional subunit vaccines, including poor mucosal immunogenicity, instability in the gastrointestinal tract and safety concerns related to endotoxin activity. Our findings highlight the potential of combining genetic engineering with nanoparticle‐based delivery to create a versatile platform for inducing robust systemic and mucosal immunity against multiple zoonotic pathogens.

In this study, the genetic modification of BBVs reduced endotoxin activity but maintained their adjuvant properties. By targeting lipid A biosynthesis genes (*msbB*, *pagP*, *pagL* and *lpxE*), a mutant strain with penta‐acylated monophosphorylated lipid A was established, which substantially reduced systemic pro‐inflammatory cytokine production compared to that in the wild‐type SC014 strain. Importantly, this modification does not compromise the ability of BBVs to activate BMDCs or function as an adjuvant to elicit antigen‐specific T cell responses. The maturation of BMDCs in vitro, along with the enlargement of LNs in vivo, further substantiated this observation. This finding aligns with previous reports indicating that reducing lipid A acyl chains and phosphate groups decreases TLR4 activation while preserving the immunostimulatory capacity (Bai et al. 2025; Irene et al. 2019; Shen et al. 2024). This delicate balance between safety and efficacy addresses the challenges posed by high endotoxin activity in the development of BBV‐based vaccines.

The success of oral vaccines depends on their ability to overcome gastrointestinal barriers, such as enzymatic degradation and inadequate antigen uptake (Gonzalez‐Cruz and Gill 2021). The COS‐coated CSS‐BBV platform demonstrated enhanced stability in SGF and SIF, likely owing to COS‐mediated protection against pH extremes and bile acids, consistent with previous research findings of COS providing protection against gastrointestinal environment damage (Liu et al. 2019; Yuan et al. 2025). Furthermore, the cationic COS layer may facilitate electrostatic interactions with the intestinal mucus, thereby prolonging the mucosal retention time and enhancing antigen uptake by gut‐associated lymphoid tissue, essential for inducing sIgA. Notably, the high sIgA antibody titer induced by the dual‐antigen GDH‐gD‐Fc‐CSS‐BBV@COS formulation suggests the potential of nanoparticle‐mediated mucosal delivery as an alternative to viral or bacterial vaccine vectors, thereby mitigating safety risks.

The SpyCatcher/SpyTag and SpG/Fc systems afforded efficient loading of heterologous antigens (GDH from *S. suis* 2 and gD from PRV) onto the surface of CSS‐BBV. Such modular design offers significant advantages in facilitating rapid antigen replacement, whereas conventional gene fusion strategies typically require extensive and laborious optimization for each antigen. Additionally, the Fc domain may further enhance antigen presentation by binding to Fc receptors on the antigen‐presenting cells, thereby synergizing with the inherent DC activation properties of CSS‐BBVs. The flexible antigen display capability of our platform is crucial for addressing pathogen spillover events caused by global environmental change. In the future, researchers may expand this approach to incorporate conserved epitopes from other priority pathogens, such as influenza viruses and coronaviruses, providing new strategies for the development of vaccines against zoonotic pathogens.

Complete protection against lethal *S. suis* 2 and PRV challenges illustrated the capacity of our platform to concurrently target diverse pathogen types. In the case of the *S. suis* 2 infection, the host initiates both cellular and humoral immune responses, releasing numerous cytokines and specific antibodies. Additionally, M1 polarization of macrophages may play a significant role in promoting antigen presentation and bacterial clearance (Chen et al. 2023; Chen et al. 2024). For PRV, the robust IgG2a/IgG1 ratio and IFN‐γ^+^ CD8^+^ T‐cell responses indicate a Th1‐biased immune response, which is essential for the clearance of this intracellular virus (Liu et al. 2021; Doedens et al. 2013). The accumulation of CSS‐BBVs in the inguinal LNs may also enhance antigen cross‐presentation via MHC‐I, thereby explaining the observed activation response of CD8^+^ T cells, which is less commonly observed with soluble subunit vaccines. Additionally, resistance to PRV may depend on the generation of neutralizing antibodies and sIgA, a mechanism that aligns with previous findings from studies of gD‐based subunit vaccines (Wang et al. 2019; Ren et al. 2023). Moreover, mucosal immunity–induced sIgA antibodies are not only widely present at local immune sites but can also migrate to distal locations (Bian et al. 2024; Chiu et al. 2023; Su et al. 2021; Lin et al. 2016; Hu et al. 2001). Therefore, we sought to investigate whether oral immunization with our vaccine could induce protective immunity in distal mucosal sites of mice, such as the reproductive tract. The observed high levels of antigen‐specific sIgA in the vaginal lavage showed that activated antigen‐presenting cells in gut‐associated lymphoid tissue migrated to distal mucosal sites. This dual effect highlights the advantage of combined vaccines for controlling zoonotic diseases, particularly for managing mixed infections caused by bacteria and viruses.

Although our findings are encouraging, several challenges must be addressed before clinical application. First, although COS enhance gastrointestinal stability, the durability of the immune response following oral administration requires further investigation. Second, although evidence suggests that *S. choleraesuis*, which is more readily cleared by the immune system than by gut‐resident probiotics, does not induce immune tolerance, further experimental validation is required to confirm this hypothesis. Given the strong adjuvant activity of the platform, long‐term safety assessments are needed, especially those related to autoimmune responses triggered by BBV bacterial components.

## 5 Conclusion

A novel oral delivery system based on the CSS‐BBV that can flexibly display double‐fusion antigens effectively overcame the limitations posed by the gastrointestinal environment and achieved effective antigen delivery. This study presents a blueprint for the development of next‐generation oral vaccines that target zoonotic threats. By integrating lipid A modification, modular antigen display, and COS‐mediated mucosal targeting technologies, the CSS‐BBV platform has effectively addressed the historical barriers in subunit vaccine design. This platform can induce a balanced Th1/Th2 immune response, humoral immunity and a robust mucosal immune response, making it a viable alternative to traditional injectable vaccines. As the integrated One Health approach has received increasing attention with regard to the preparedness for future pandemics (Gurley and Plowright 2025; Schmiege et al. 2020), our highly adaptable multi‐pathogen vaccine platform could be important for mitigating interconnected infectious diseases that affect both human and non‐human animal populations.

## Author Contributions

**Xuegang Shen**: writing–original draft, software, methodology, data curation, visualization. **Shujie Wang**: methodology, resources, data curation. **Kunying Qiu**: investigation, validation. **Zeqing Liu**: investigation, validation. **Xiaoxiao Tian**: investigation, validation. **Fandan Meng**: investigation. **Yan‐Dong Tang**: investigation. **Haiwei Wang**: investigation. **Mingxia Sun**: investigation. **Xue‐Hui Cai**: conceptualization, supervision, resources. **Tong‐Qing An**: conceptualization, supervision, funding acquisition, writing–review and editing. **Yong‐Bo Yang**: conceptualization, supervision, writing–review and editing, funding acquisition, project administration, data curation, visualization.

## Funding

This study was supported by grants from the National Key Research and Development Program of China (2022YFD1800300), the Natural Science Foundation of Heilongjiang Province (ZD2023C005), the Agricultural Science and Technology Innovation Program (CAAS‐CSLPDCP‐202301), the State Key Laboratory for Animal Disease Control and Prevention (SKLADCPKFKT202405), the Technology Integration and Demonstration Program for High‐Yield and High‐Efficiency Agriculture Program of Chinese Academy of Agricultural Sciences (ZHJGCGX202516), Basic Research Center, Innovation Program of Chinese Academy of Agricultural Sciences (CAAS‐BRC‐LPDC‐2025‐02), and the Central Public‐Interest Scientific Institution Basal Research Fund (No. 1610302022019 and 1610302024007).

## Conflicts of Interest

The authors declare no competing interests.

## Supporting information



Engineered Low‐Endotoxin Bacterial Biomimetic Vesicles for Enhanced Oral Dual‐Antigen Subunit Vaccine Delivery

**Supplementary Figure 1**: PCR identification of SC mutant strains.

**Supplementary Figure 2**: Mass spectrometry analysis of SC and SC‐L3 lipid A structures.

**Supplementary Figure 3**: Expression of mCherry on the surfaces of *Salmonella choleraesuis* strain (SC)‐L3 and biomimetic vesicles (BBVs).

**Supplementary Figure 4**: Expression and purification of CSS‐biomimetic vesicles (BBVs) and mCherry‐SpyTag by SDS‐PAGE.

**Supplementary Figure 5**: Efficiency of mCherry‐SpyTag conjugation to ClyA‐SpyCatcher on the CSS‐BBV surface verified using Western blotting. Nano‐flow cytometry was used to detect mCherry‐SpyTag binding to CSS‐BBV.

**Supplementary Figure 6**: Flow cytometry analysis of the expression of CD86 and CD206 surface molecules on RAW264.7 cells stimulated with mCherry and mCherry‐CSS‐BBV@COS for 24 h.

**Supplementary Figure 7**: *In vitro* fluorescence imaging of mCherry fluorescence in BBVs.

**Supplementary Figure 8**: *In vitro* SC bactericidal activity assessment of the week 5 serum at different dilution ratios after immunization with mCherry‐CSS‐BBV@COS.

**Supplementary Figure 9**: Purification and identification of GDH‐SpyTag and gD‐Fc proteins.

**Supplementary Figure 10**: Particle size analysis of CSS‐BBVs conjugated with dual antigens.

**Supplementary Figure 11**: Determination of the efficiency of conjugation between CSS‐BBVs and the target protein.

**Supplementary Figure 12**: ELISA detection of gD‐Fc exposure on the surface of GDH‐gD‐Fc‐CSS‐BBV@COS.

**Supplementary Figure 13**: Flow cytometry analysis of GDH‐gD‐Fc‐CSS‐BBV@COS promoting M1 polarization in RAW264.7 cells.

**Supplementary Figure 14**: Body temperature changes induced by oral immunization with GDH‐gD‐Fc‐CSS‐BBV@COS in mice.

**Supplementary Figure 15**: Detection of inflammatory factors induced by oral immunization with GDH‐gD‐Fc‐CSS‐BBV@COS in mice.

**Supplementary Figure 16**: Flow cytometry analysis of the proportions of CD3^+^CD8^+^ and CD3^+^CD4^+^ T cells in the spleen.

**Supplementary Figure 17**: Detection of neutralizing antibodies against PRV in the serum of immunized mice; **Table S1**: Primers used in this study.

## Data Availability

Data will be made available on request.
