# Supplementary material for: Engineered Low‐Endotoxin Bacterial Biomimetic Vesicles for Enhanced Oral Dual‐Antigen Subunit Vaccine Delivery
Source: J Extracell Vesicles. 2025 Nov 29;14(12):e70207. doi: 10.1002/jev2.70207 (PMC12663866; doi:10.1002/jev2.70207)
Supplement: Supplementary file 1 — Engineered Low‐Endotoxin Bacterial Biomimetic Vesicles for Enhanced Oral Dual‐Antigen Subunit Vaccine Delivery [file JEV2-14-e70207-s018.docx]

Supporting Information for

Engineered Low-Endotoxin Bacterial Biomimetic Vesicles for Enhanced Oral Dual-Antigen Subunit Vaccine Delivery

Xuegang Shen ^1^, Shujie Wang ^1^, Kunying Qiu ^1^, Zeqing Liu ^1^, Xiaoxiao Tian ^1^, Fandan Meng ^1^, Yan-Dong Tang ^1^, Haiwei Wang ^1^, Mingxia Sun ^1^, Xue-Hui Cai ^1, 2, *^, Tong-Qing An ^1, 3, *^, and Yong-Bo Yang ^1, 2, *^

^1^ State Key Laboratory for Animal Disease Control and Prevention, Harbin Veterinary Research Institute, Chinese Academy of Agricultural Sciences, Harbin 150069, China

^2^ Heilongjiang Veterinary Biopharmaceutical Engineering Technology Research Center, Harbin Veterinary Research Institute, Chinese Academy of Agricultural Sciences, Harbin 150069, China

^3^ Heilongjiang Provincial Key Laboratory of Veterinary Immunology, Harbin Veterinary Research Institute, Chinese Academy of Agricultural Sciences, Harbin 150069, China

* Emails: caixuehui@caas.cn; antongqing@caas.cn; yangyongbo@caas.cn


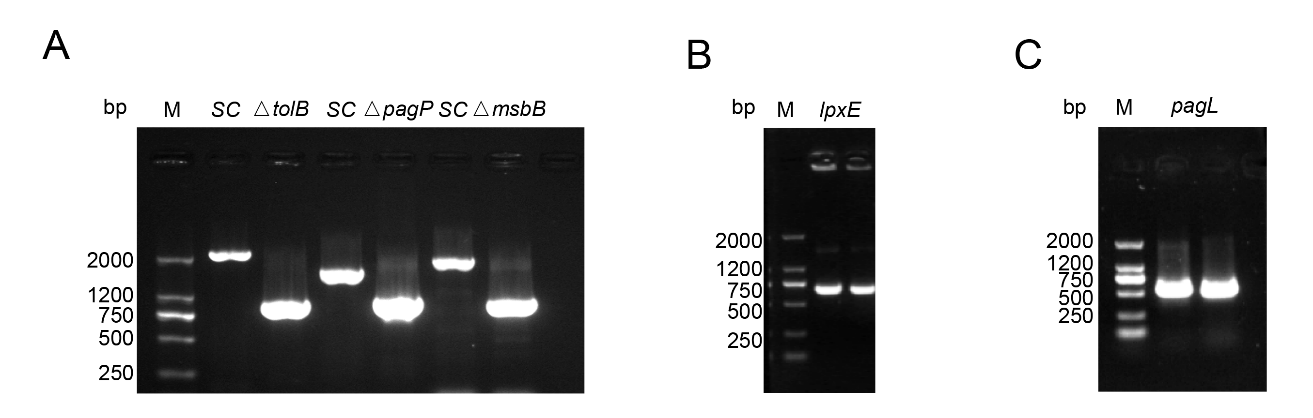


**FIGURE S1.** PCR identification of SC mutantstrains. (A–C) PCR identification of SC ∆*tolB*∆*pagP*∆*msbB* mutation (A), SC *lpxE* (B), and *pagL* (C) gene insertions. SC, *Salmonella choleraesuis*.


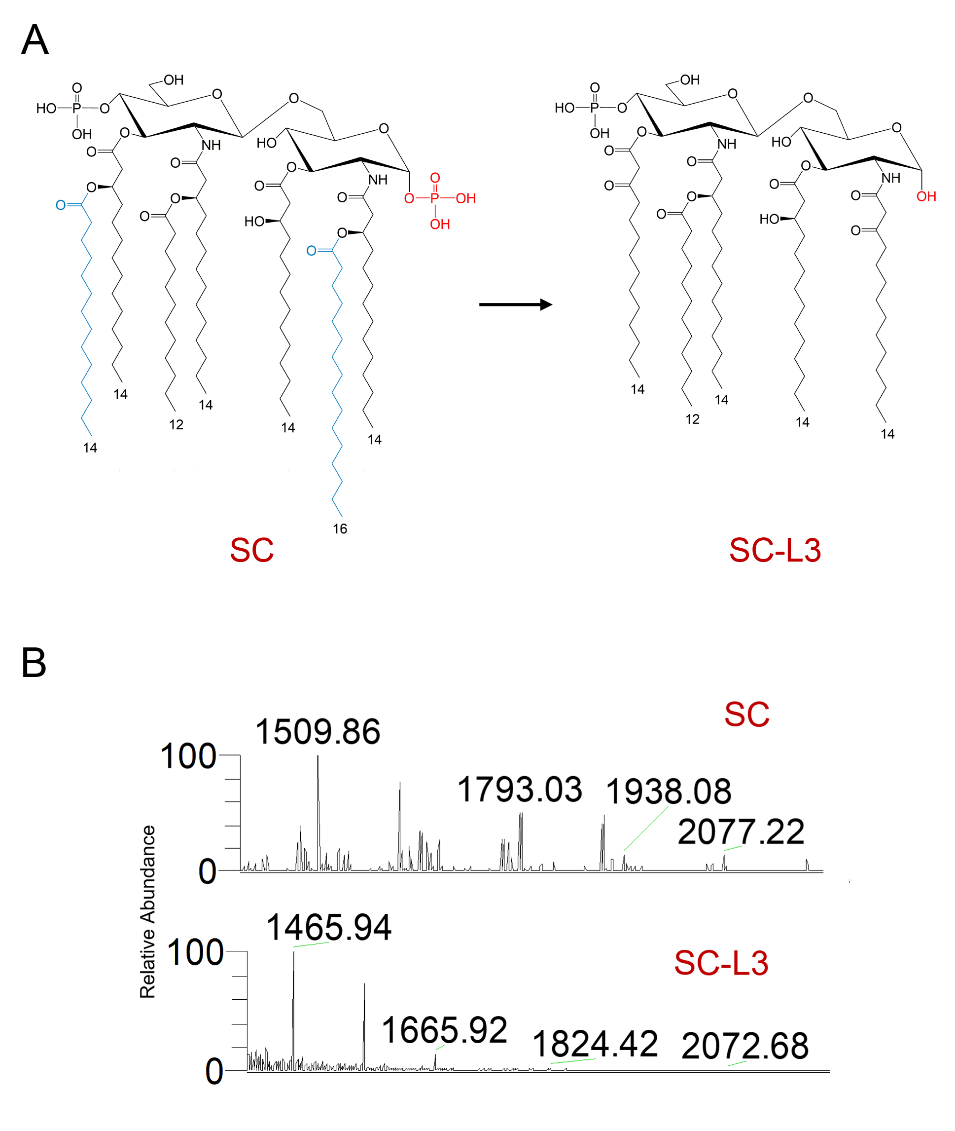


**FIGURE S2.** Mass spectrometry analysis of SC and SC-L3 lipid A structures. (A,B) Schematic diagram of theoretical changes in lipid A structure (A) and Autoflex Speed MALDI-TOF analysis of SC and SC-L3 (B). SC, *Salmonella choleraesuis.*


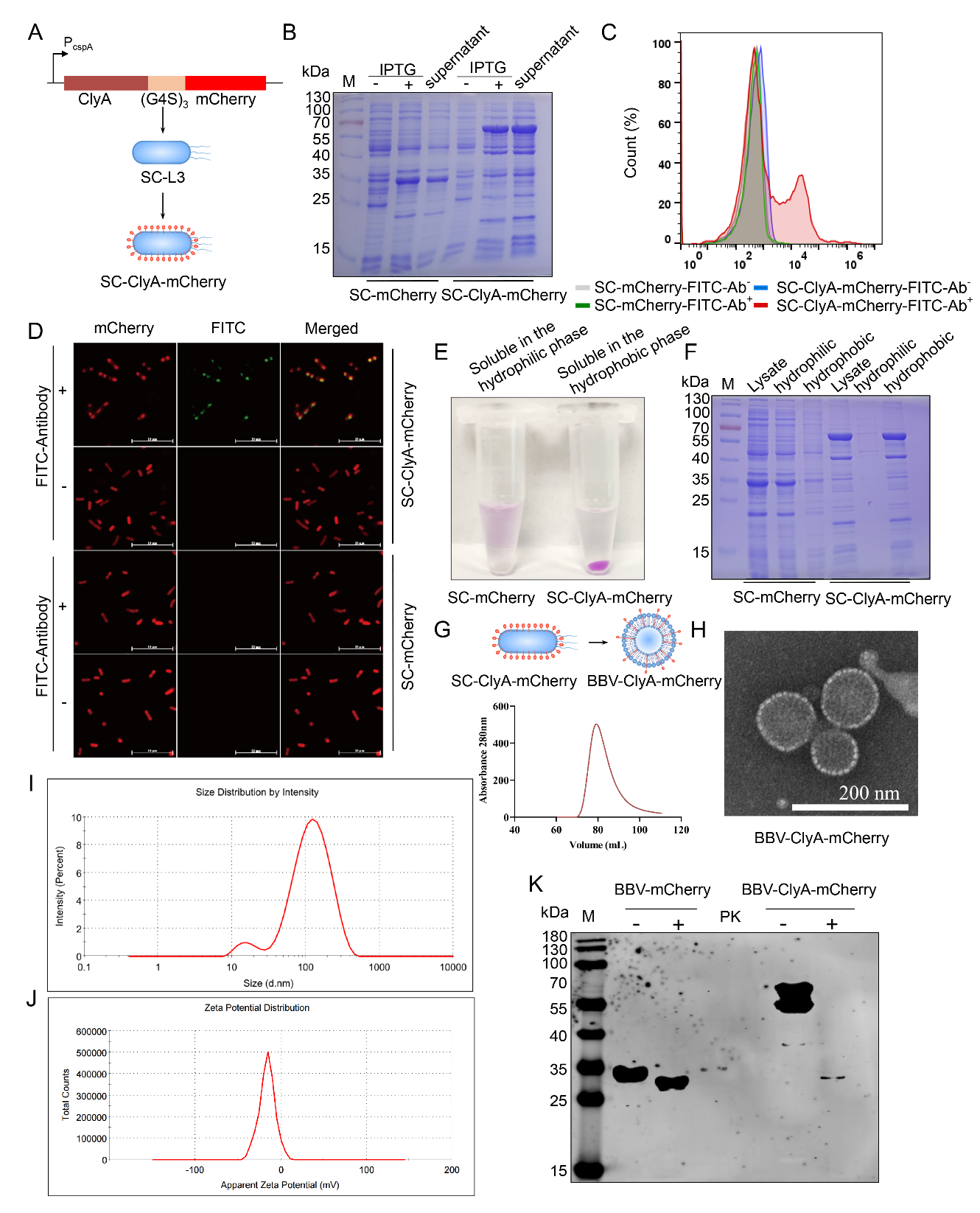


**FIGURE S3.** mCherry was exposed on the surfaces of *Salmonella choleraesuis* strain (SC)-L3 and biomimetic vesicles (BBVs). (A) Schematic diagram of the construction of recombinant plasmid and recombinant bacteria. (B) Verification of the expression of mCherry and ClyA-mCherry recombinant proteins using SDS-PAGE. (C,D) Flow cytometry (C) and confocal microscopy (D) were used to validate the localization of mCherry on the surface of SC-L3. (E, F) The extraction (E) and SDS-PAGE identification (F) of outer membrane component proteins from recombinant strains SC-ClyA-mCherry and SC-mCherry revealed that the mCherry protein expressed by the former is predominantly located in the outer membrane (soluble in the hydrophobic phase). (G) The elution peak of BBV-ClyA-mCherry purified by molecular sieve. (H–J) Transmission electron miscopy (H) scale bar = 200 nm and NanoZS90 nanoparticle size analyzer was used for the analysis of particle size (I) and zeta potential (J) of BBV-ClyA-mcherry (n = 3). K) Western blot analysis of BBV-ClyA-mcherry after Proteinase K (PK) digestion was performed to determine the localization of mCherry on BBV.


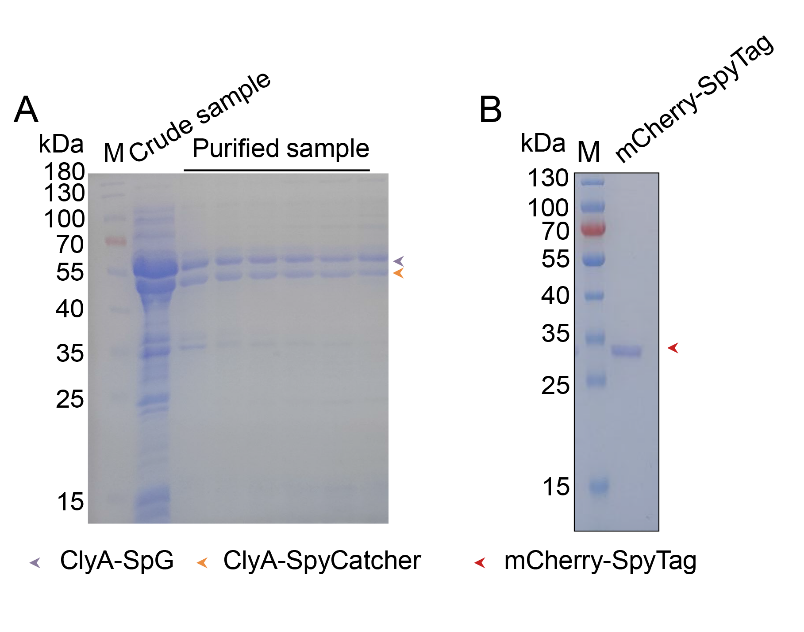


**FIGURE S4.** Expression and purification of CSS-biomimetic vesicle (BBV) and mCherry-SpyTag. (A) SDS-PAGE validation of CSS-BBV after molecular sieve chromatography. (B) SDS-PAGE validation of mCherry-SpyTag fusion protein (red arrow) purified by nickel column.


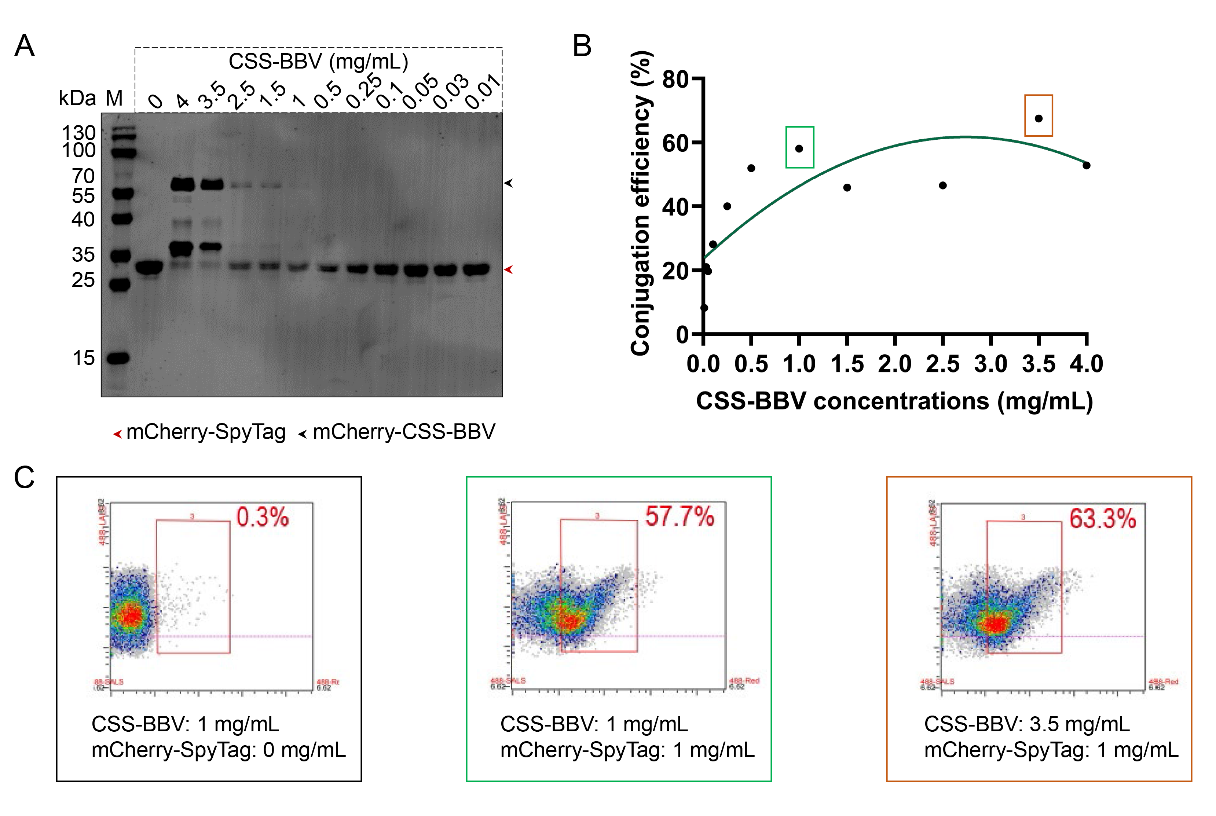


**FIGURE S5.** (A,B) The conjugation efficiency of mCherry-SpyTag to ClyA-SpyCatcher on the CSS-BBV surface was verified using western blotting using an anti-mCherry antibody (A) and corresponding data statistical analysis was performed (B) (Gray scale simulation analysis was conducted using the ImageJ software to quantify the electrophoretic band intensity of mCherry-SpyTag). (C) Nano-flow cytometry was used to detect the binding of mCherry-SpyTag to CSS-biomimetic vesicle (BBV; at concentrations of 1 and 3.5 mg/mL).


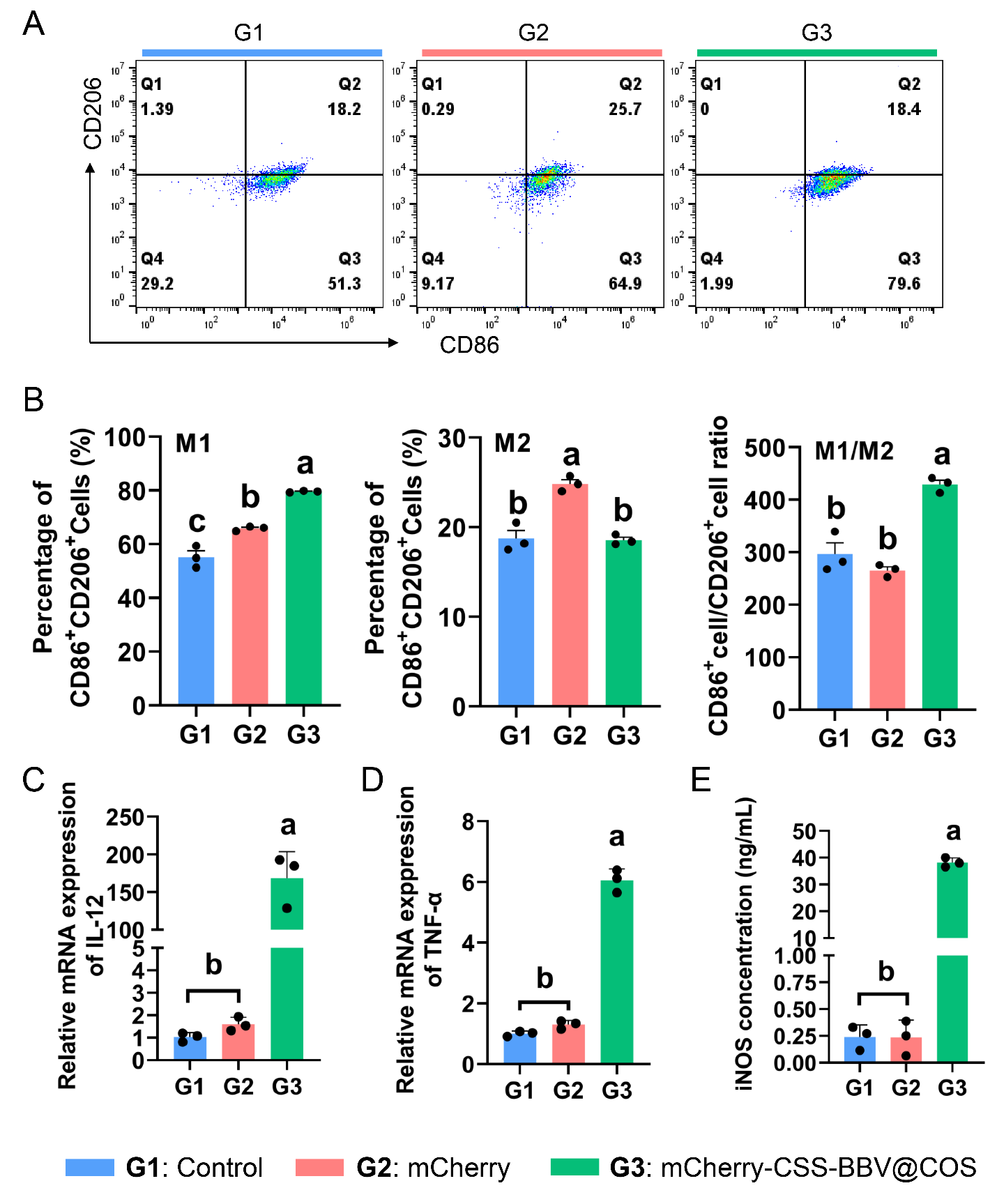


**FIGURE S6.** (A,B) Flow cytometry analysis of the expression of CD86 and CD206 surface molecules on RAW264.7 cells stimulated with mCherry and mCherry-CSS-BBV@COS for 24 h (A), and statistical analysis of M1 (CD86), M2 (CD206), and M1/M2 (B). (C,D) Fluorescent quantitative PCR was used to detect the relative mRNA transcription levels of cytokines IL-12 (C) and TNF-α (D) in RAW 264.7 cells stimulated by mCherry and mCherry-CSS-BBV@COS. (E) ELISA was used to detect the expression level of iNOS in RAW 264.7 cells stimulated by mCherry and mCherry-CSS-BBV@COS.


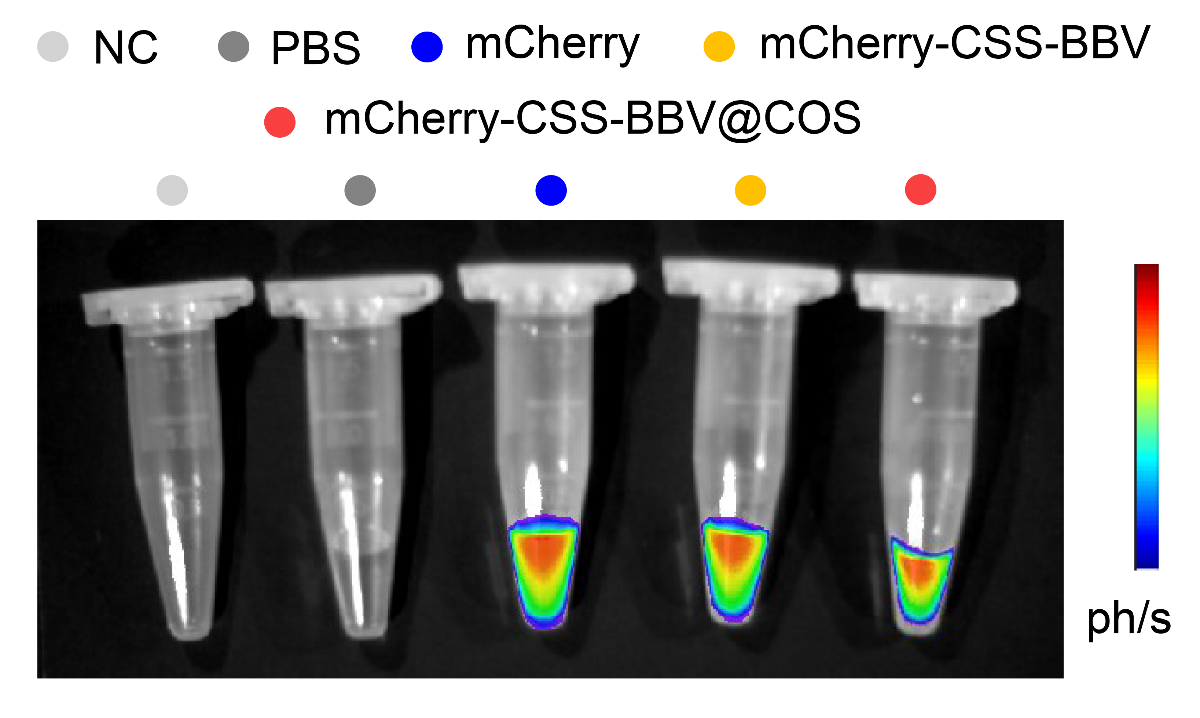


**FIGURE S7.** *In vitro* fluorescence imaging to demonstrate the specific fluorescence of mCherry in BBVs.


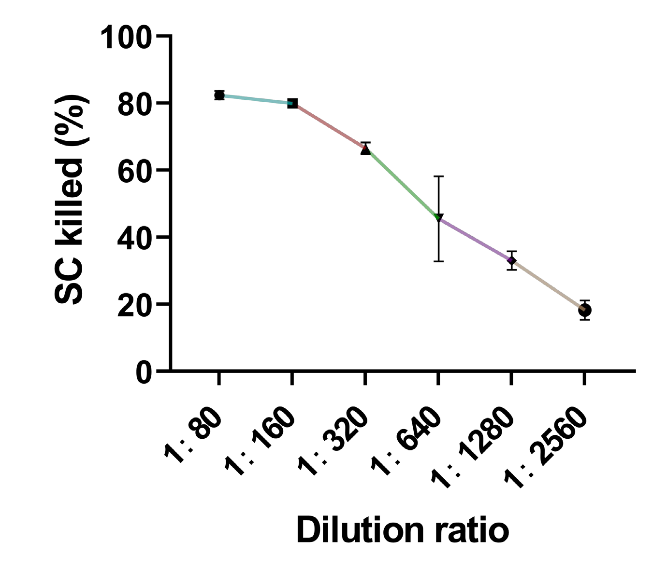


**FIGURE S8.** *In vitro* SC bactericidal activity assessment of the week 5 serum at different dilution ratios after mCherry-CSS-BBV@COS immunization.


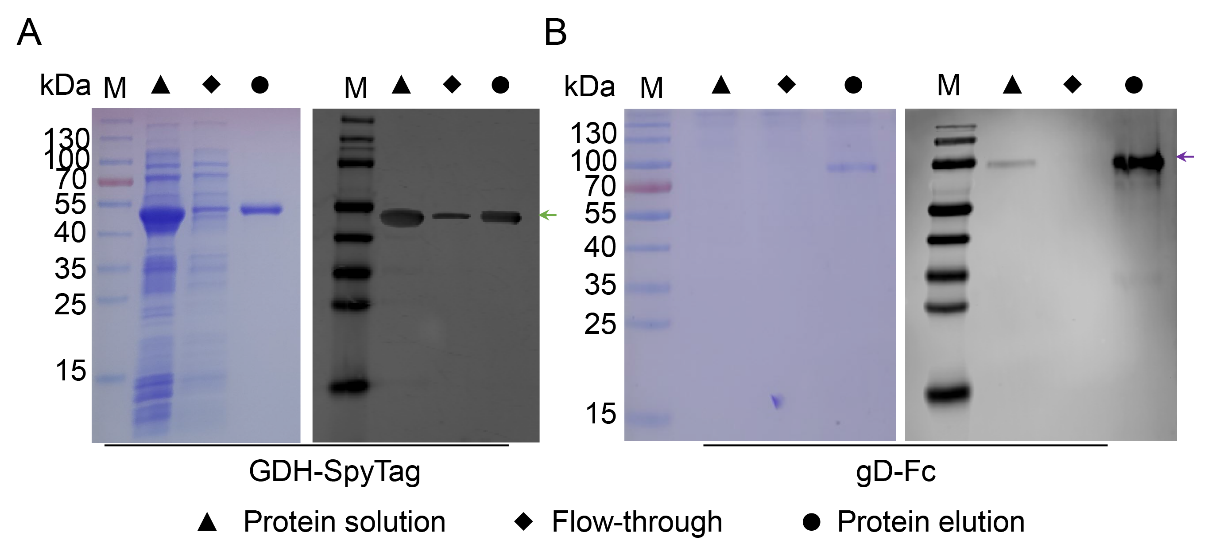


**FIGURE S9.** (A,B) Purification and and identificationof GDH-SpyTag and gD-Fc proteins. Coomassie Brilliant Blue staining or western blotting were used for the identification of purified GDH-SpyTag (A), and purified gD-Fc (B).


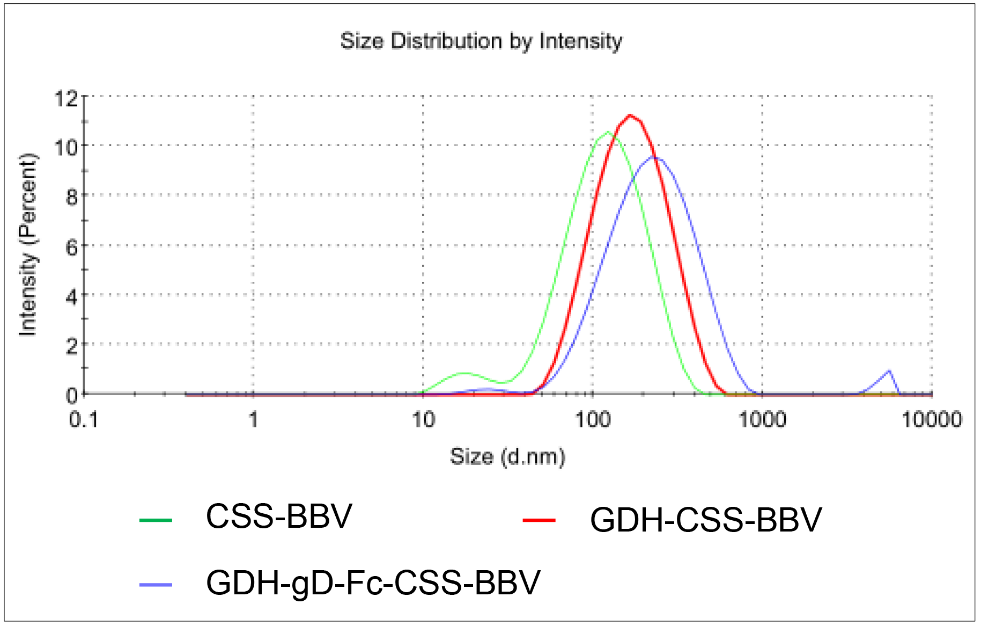


**FIGURE S10.** The CSS-BBV conjugated dual antigen was confirmed through particle size analysis, showing a significant increase in the particle size of the GDH-gD-Fc-CSS-BBV (from 94.91 ± 0.96 nm to 195.57 ± 1.14 nm).


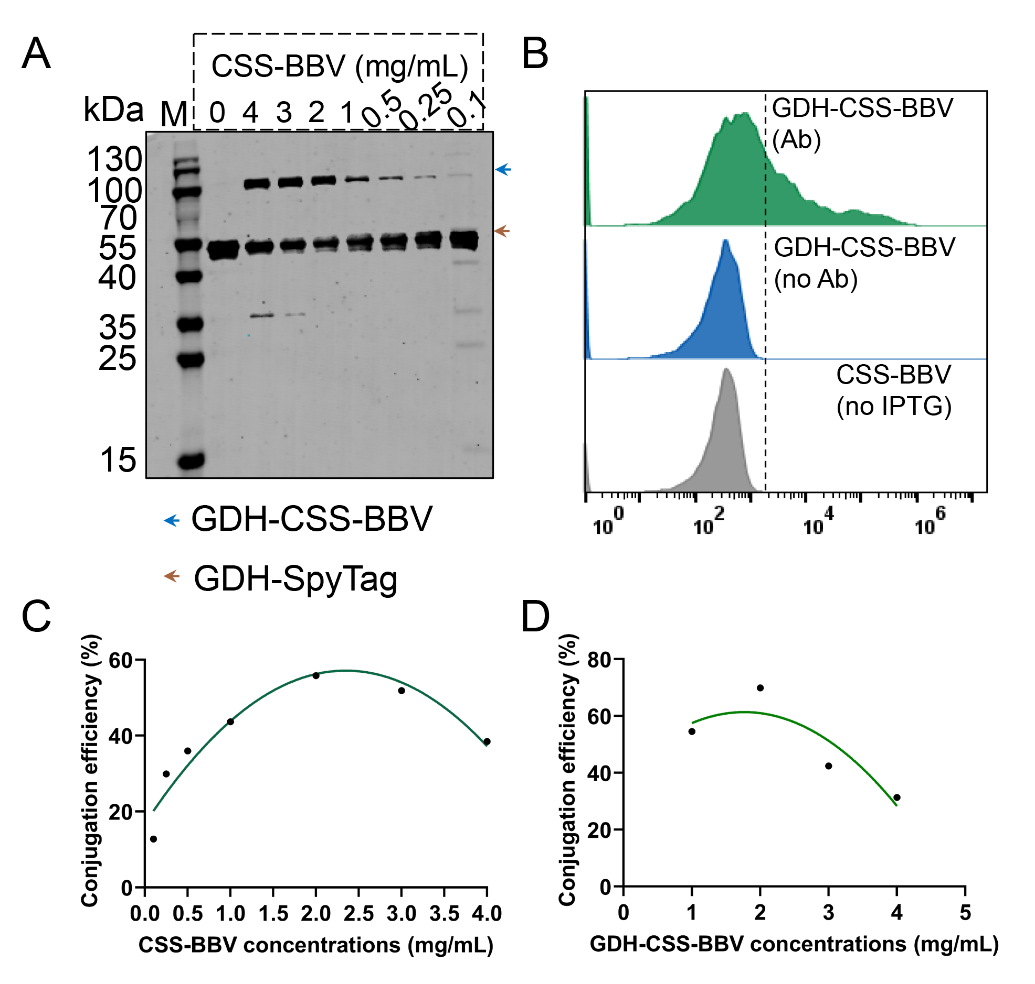


**FIGURE S11.** Determination of the conjugation efficiency between CSS-BBV and the target protein. (A,C) The efficiency of CSS-BBV conjugation with GDH-SpyTag was determined using western blotting (A) and quantification of band intensity (C). (B) The GDH-CSS-BBV conjugated fluorescent antibody was determined using flow cytometry. (D) Detection of the conjugation efficiency of GDH-CSS-BBV to gD-Fc.


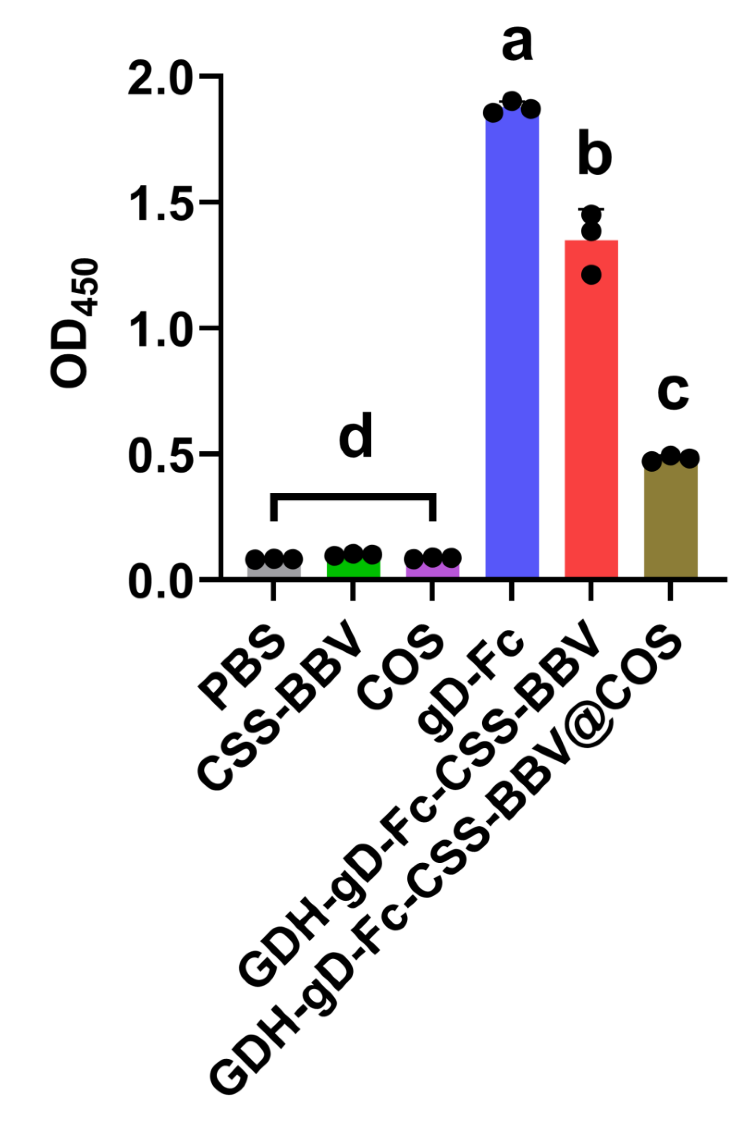


**FIGURE S12**. ELISA detection of gD-Fc exposure on the surface of GDH-gD-Fc-CSS-BBV@COS.


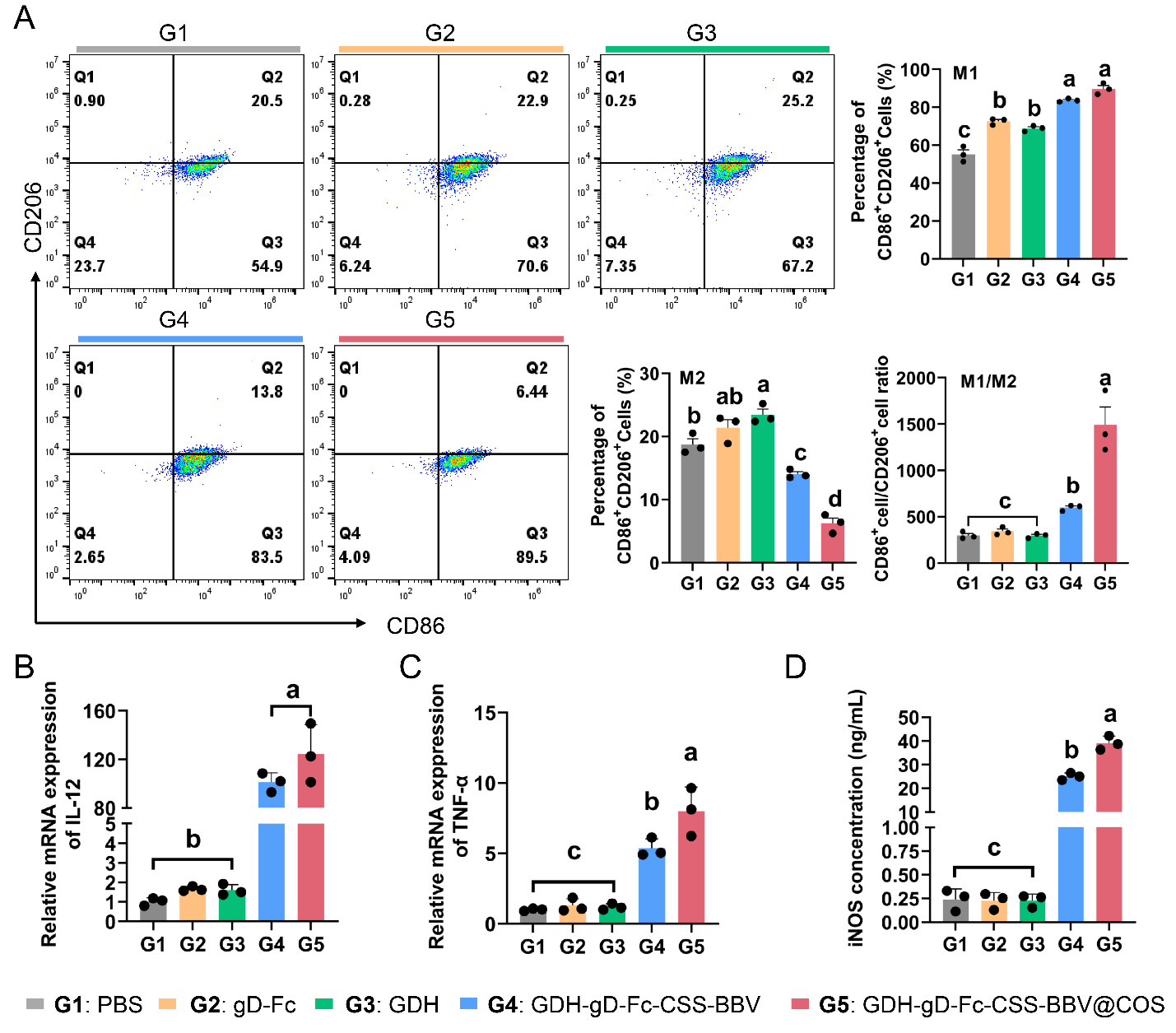


**FIGURE S13.** GDH-gD-Fc-CSS-BBV@COS promotes cell M1 polarization. Flow cytometry of the expression of CD86 and CD206 surface molecules on RAW264.7 cells stimulated with samples for 24 h, and statistical analysis of M1 (CD86), M2 (CD206), and M1/M2. (B,C) Fluorescent quantitative PCR was used to detect the relative mRNA transcription levels of cytokines IL-12 (C) and TNF-α (D) in RAW 264.7 cells stimulated by samples. (D) ELISA was used to detect the expression level of iNOS in RAW 264.7 cells stimulated by samples.


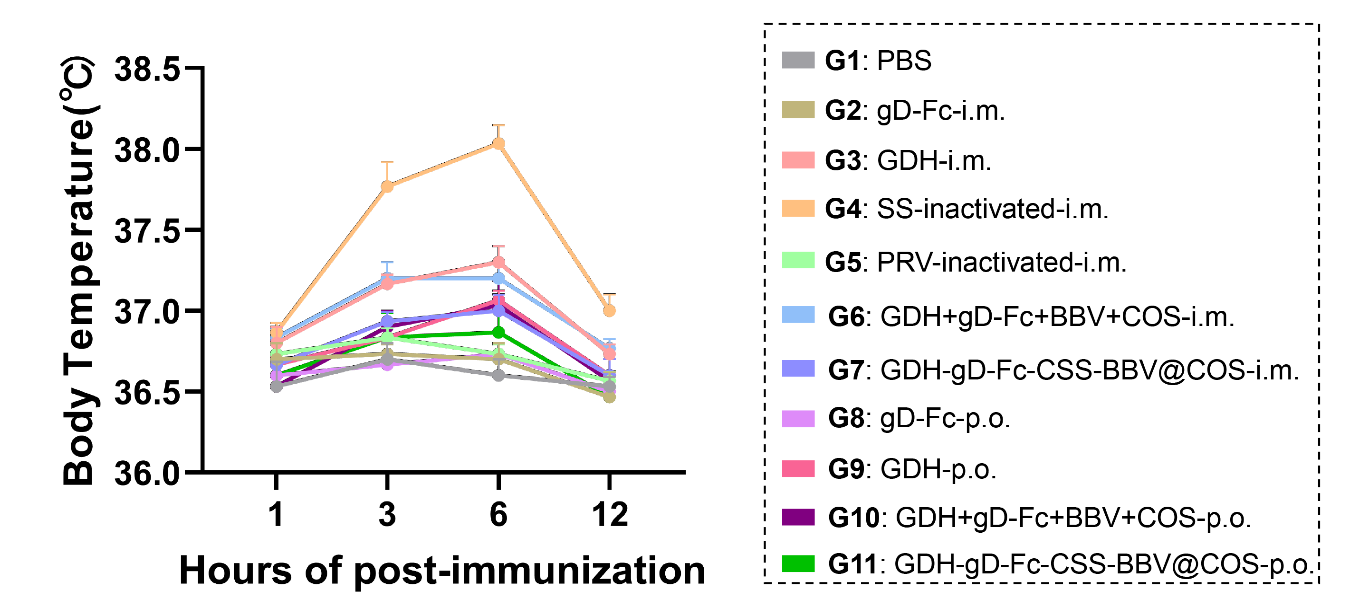


**FIGURE S14.** Oral immunization with GDH-gD-Fc-CSS-BBV@COS induces a lower inflammatory response in mice. The dynamic changes in body temperature of mice in each group within 12 h post-immunization.


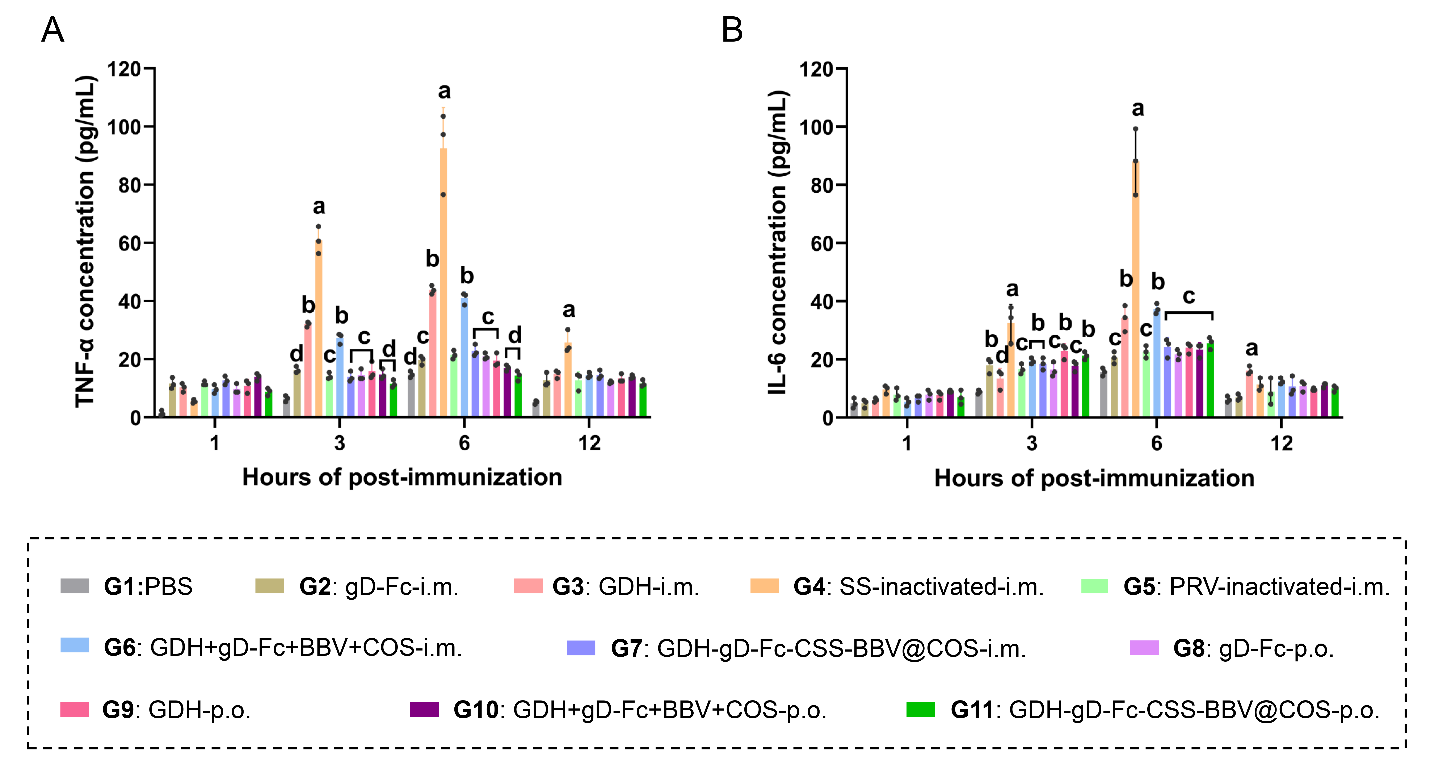


**FIGURE S15.** Oral immunization with GDH-gD-Fc-CSS-BBV@COS induces a lower inflammatory response in mice. (A,B) The dynamic changes of pro-inflammatory cytokines TNF-α (A) and IL-6 (B) in serum within 12 h post-immunization in each group of mice.


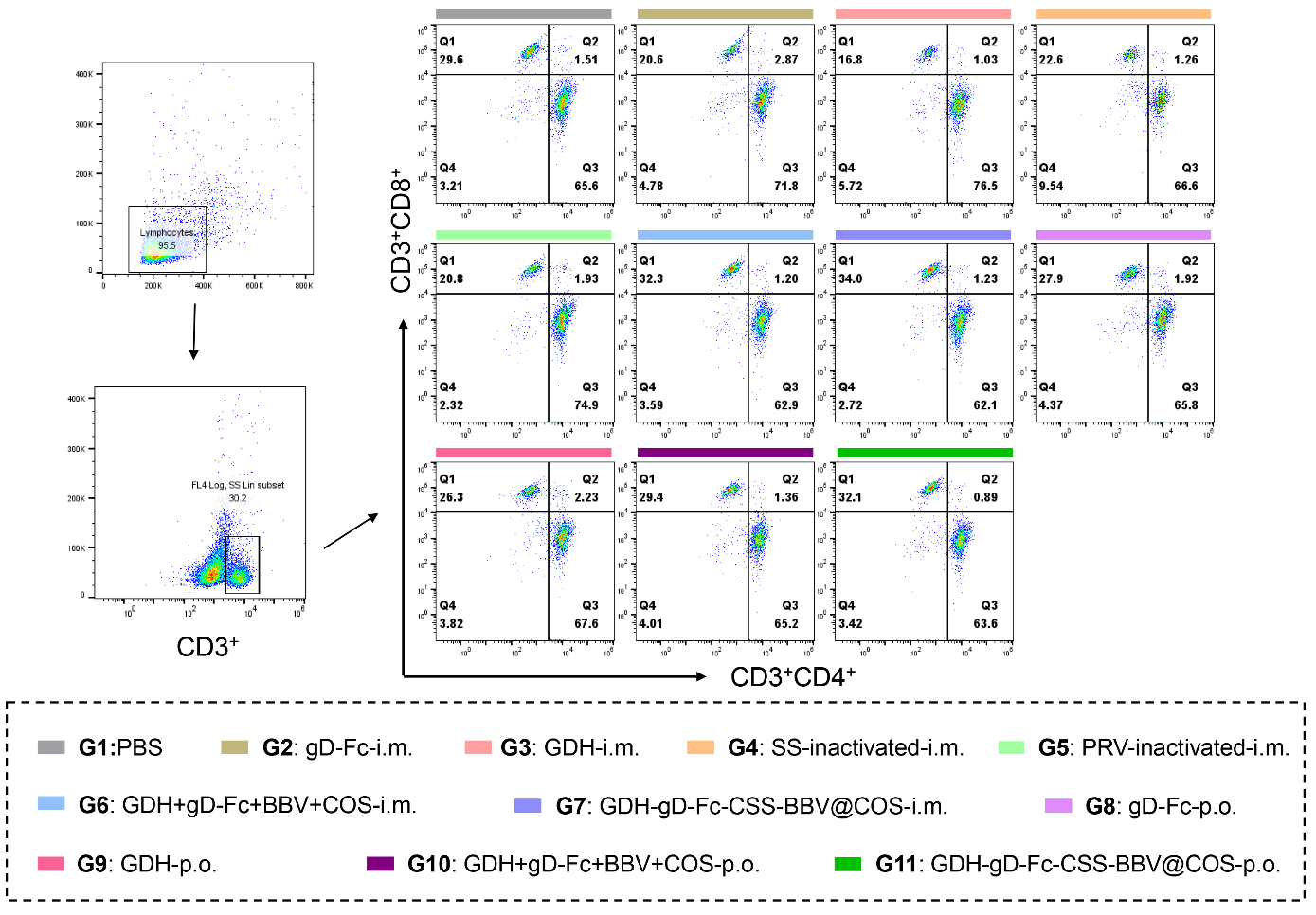


**FIGURE S16.** Flow cytometry analysis of the proportions of CD3^+^CD8^+^ T cells and CD3^+^CD4^+^ T cells in the spleen.


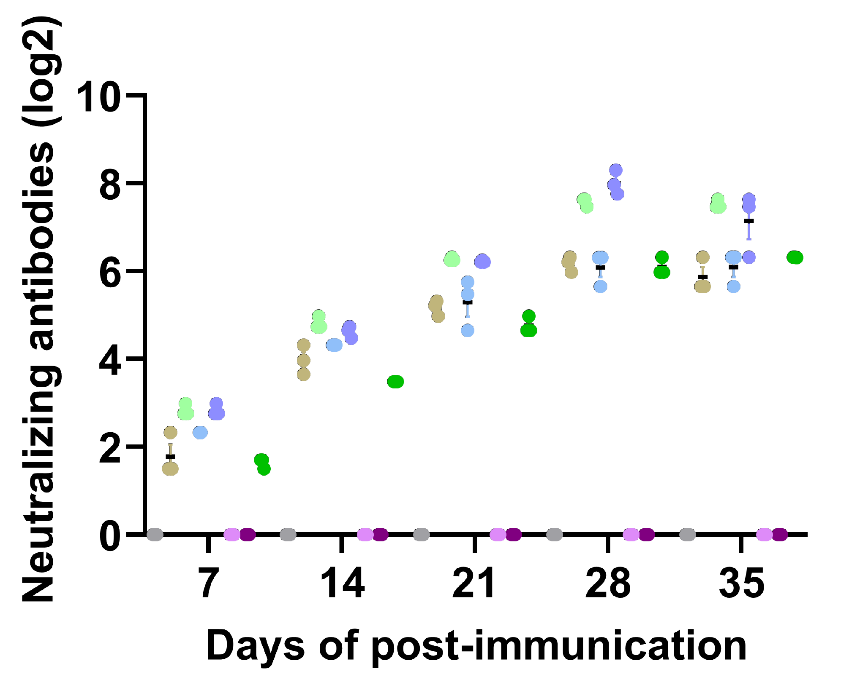


**FIGURE S17.** The neutralizing antibodies were determined against PRV in the serum of immunized mice.

**TABLE S1.** Primers used in this study.

| Primer name | Primer sequence (5′-3′) |
| --- | --- |
| 112-F | AGTGTCTACGTATTAccggg |
| 112-R | gtgtctacgtactagaagaag |
| DtolB-1F | ttctagtacgtagacacgaatggtcagccatcgaaaga |
| DtolB-1R | ttaattattacatatctcccatacctgggc |
| DmsbB-1F | ttctagtacgtagacaccgcagcggcacggcctatctg |
| DmsbB-1R | ctggaaaagcctagtcctgatataggttgac |
| DpagP-1F | ttctagtacgtagacactatcccttaatgatgtagcgc |
| DpagP-1R | cccttccccgaccgttcaaaaattcgactg |
| DtolB-2F | gggagatatgtaataattaattgattacta |
| DtolB-2R | ggTAATACGTAGACACTacgcctttaccctgcaggta |
| DmsbB-2F | tcaggactaggcttttccagggtctgctgac |
| DmsbB-2R | ggTAATACGTAGACACTatggcgaggtcgtggtcgct |
| DpagP-2F | tttgaacggtcggggaagggcattgttcag |
| DpagP-2R | ggTAATACGTAGACACTaacccgccgaaagggcgggt |
