# Supplementary figures and images for: Engineered Low‐Endotoxin Bacterial Biomimetic Vesicles for Enhanced Oral Dual‐Antigen Subunit Vaccine Delivery
Source: J Extracell Vesicles. 2025 Nov 29;14(12):e70207. doi: 10.1002/jev2.70207 (PMC12663866; doi:10.1002/jev2.70207)

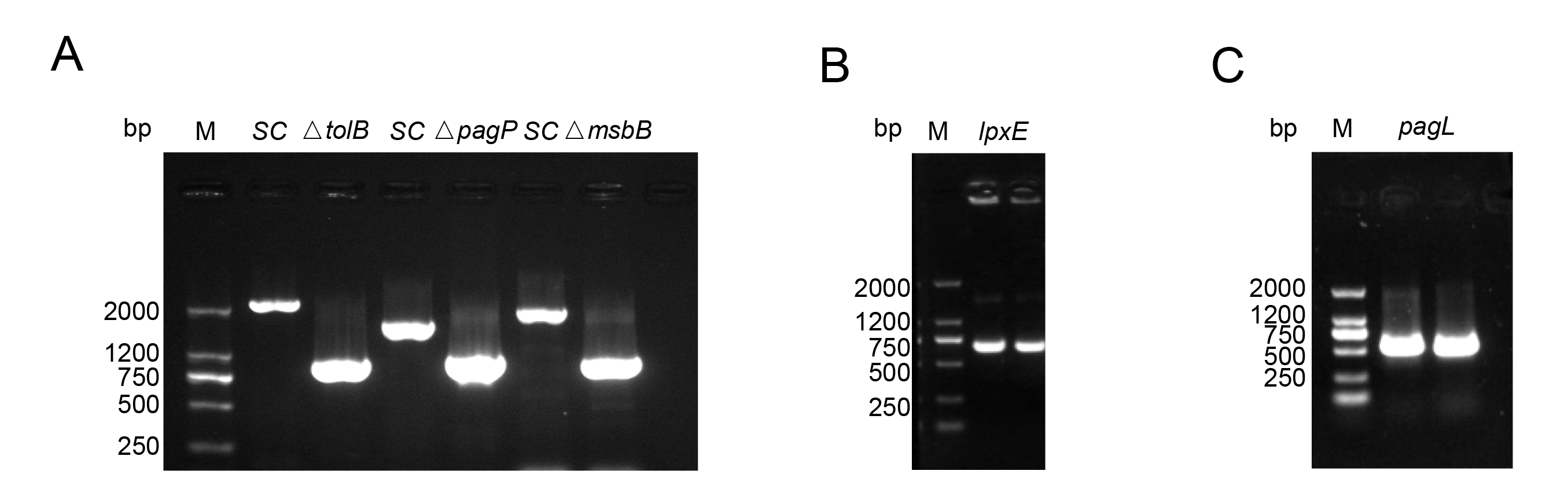

Supplement: Supplementary file 2 — Supplementary Figure 1: PCR identification of SC mutant strains. [file JEV2-14-e70207-s016.tif]

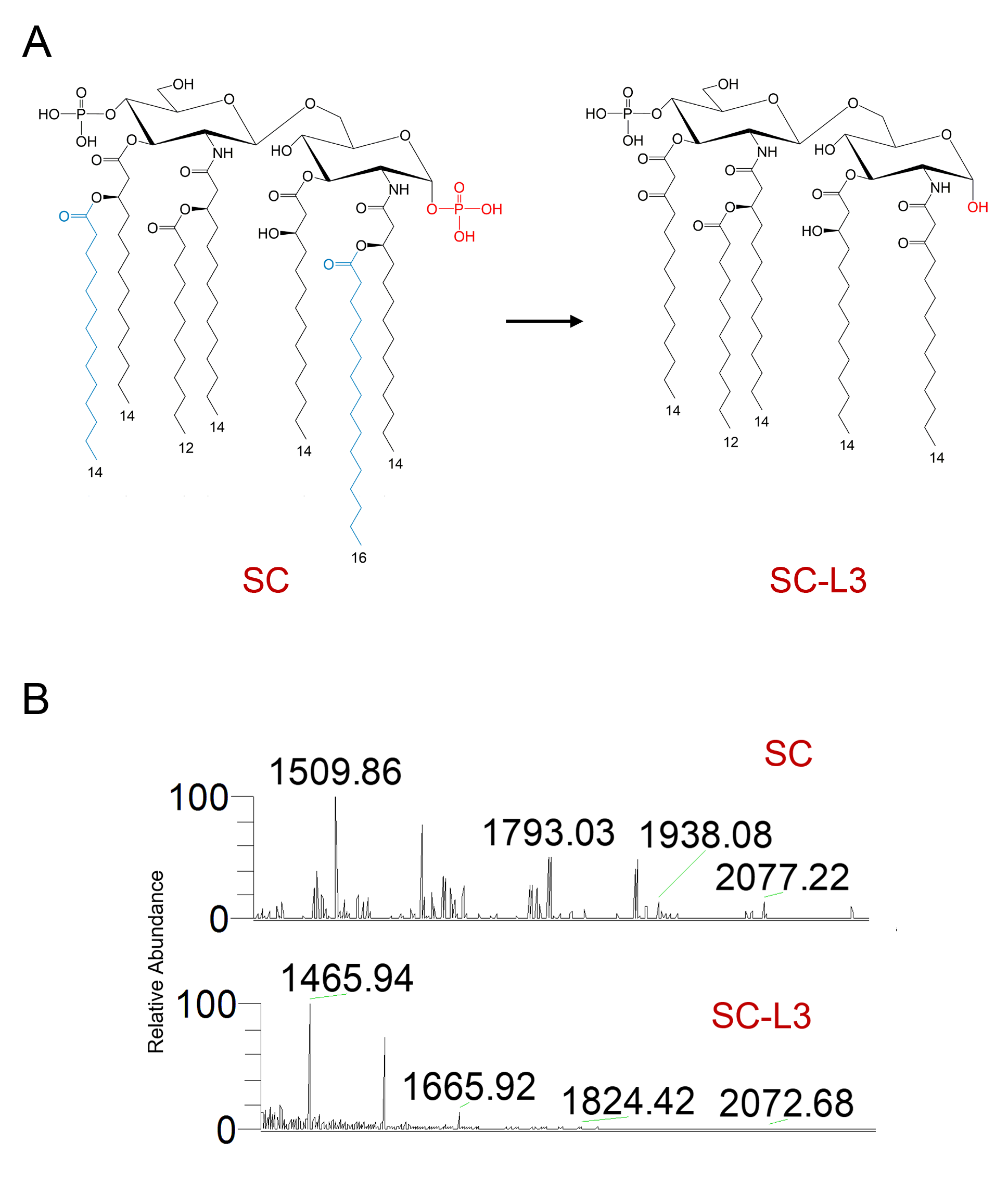

Supplement: Supplementary file 3 — Supplementary Figure 2: Mass spectrometry analysis of SC and SC‐L3 lipid A structures. [file JEV2-14-e70207-s002.tif]

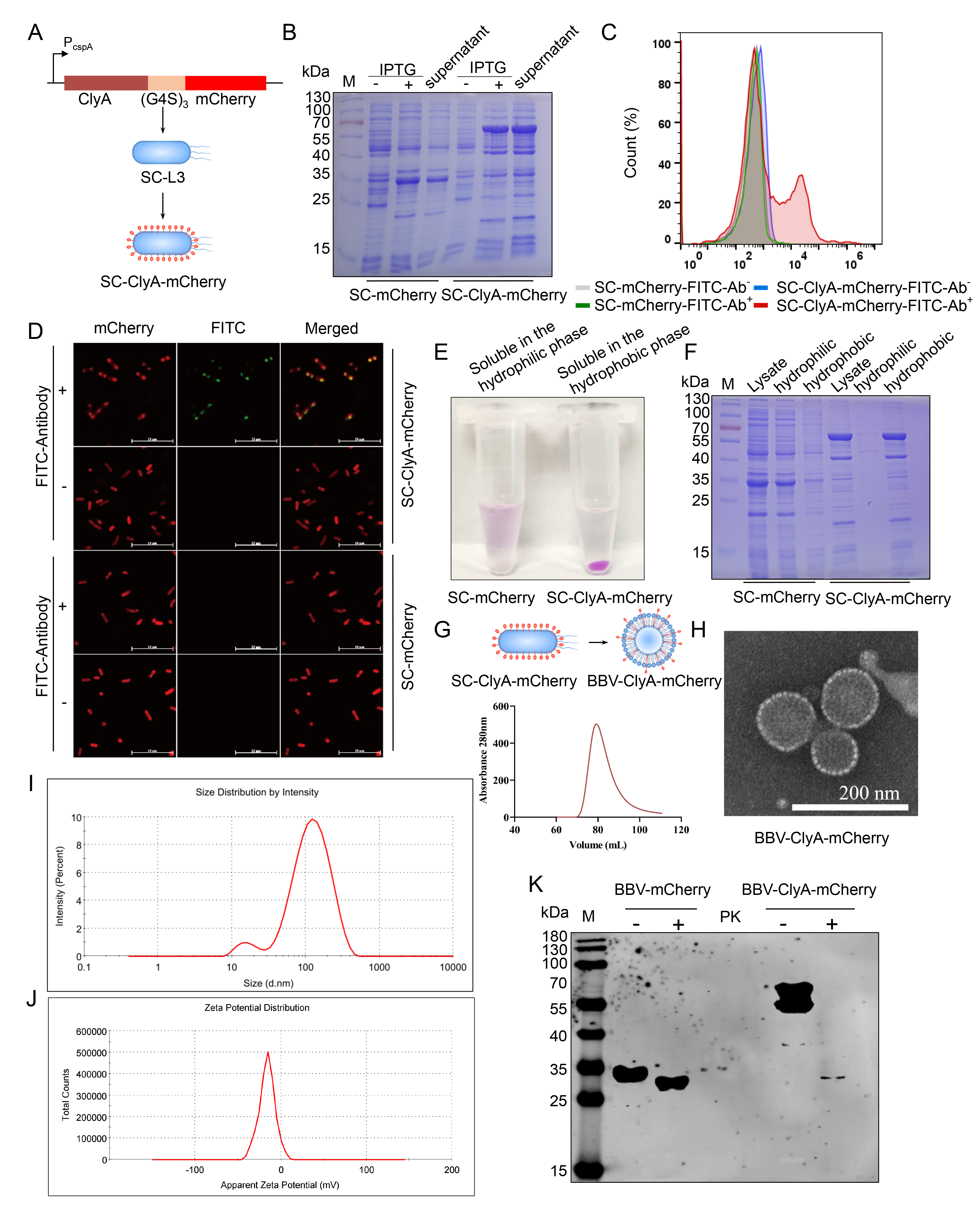

Supplement: Supplementary file 4 — Supplementary Figure 3: Expression of mCherry on the surfaces of Salmonella choleraesuis strain (SC)‐L3 and biomimetic vesicles (BBVs). [file JEV2-14-e70207-s006.tif]

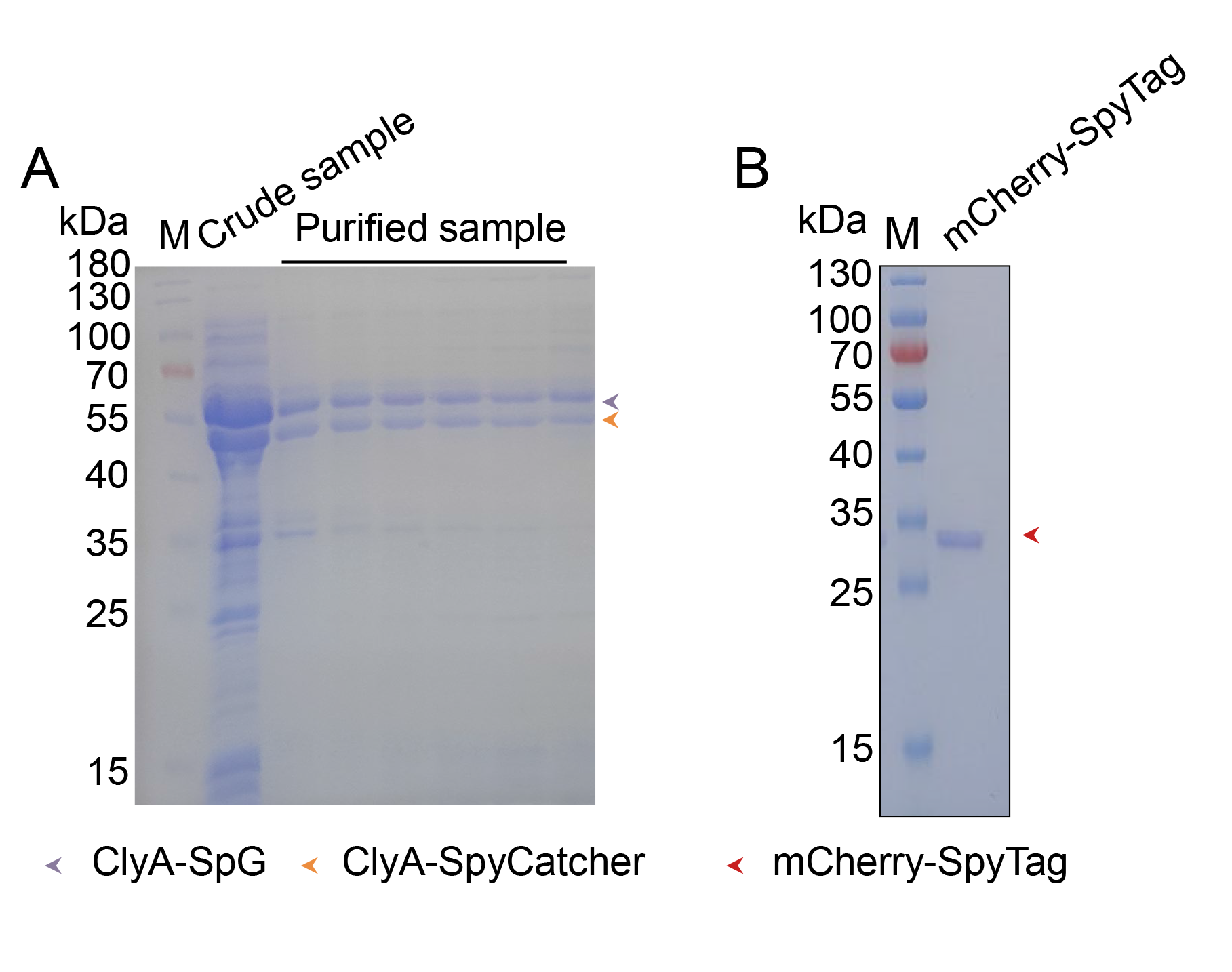

Supplement: Supplementary file 5 — Supplementary Figure 4: Expression and purification of CSS‐biomimetic vesicles (BBVs) and mCherry‐SpyTag by SDS‐PAGE. [file JEV2-14-e70207-s005.tif]

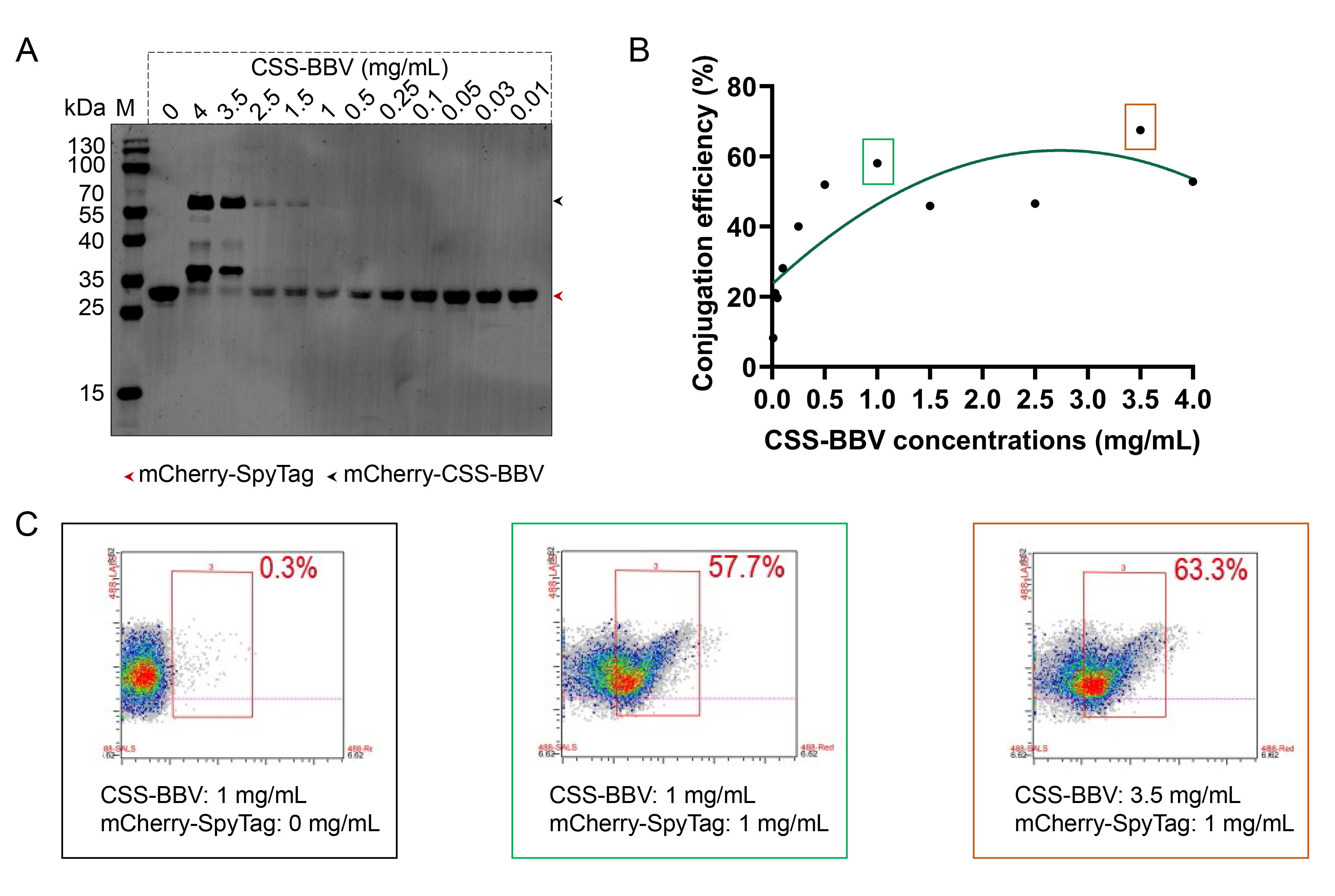

Supplement: Supplementary file 6 — Supplementary Figure 5: Efficiency of mCherry‐SpyTag conjugation to ClyA‐SpyCatcher on the CSS‐BBV surface verified using Western blotting. Nano‐flow cytometry was used to detect mCherry‐SpyTag binding to CSS‐BBV. [file JEV2-14-e70207-s008.tif]

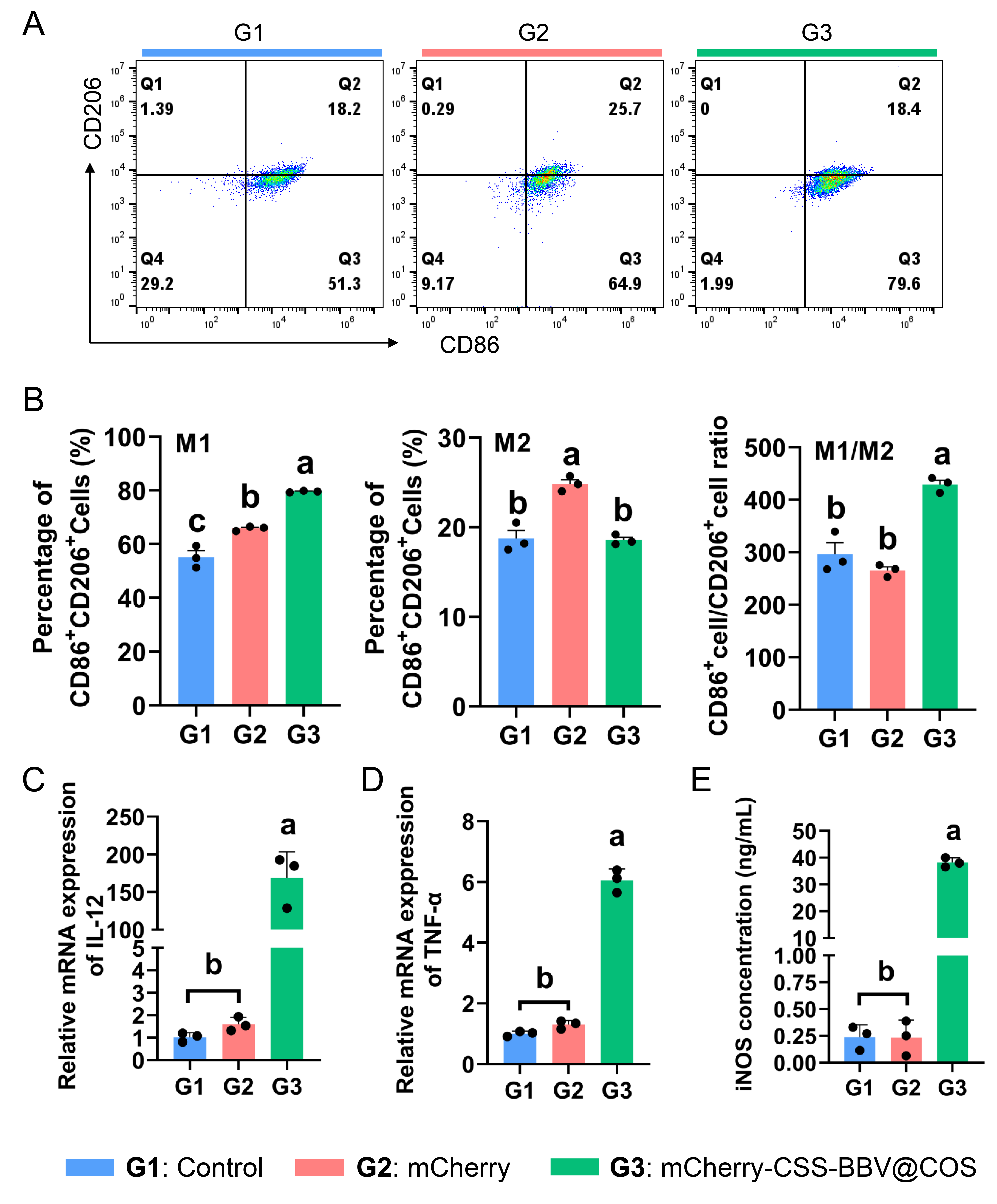

Supplement: Supplementary file 7 — Supplementary Figure 6: Flow cytometry analysis of the expression of CD86 and CD206 surface molecules on RAW264.7 cells stimulated with mCherry and mCherry‐CSS‐BBV@COS for 24 h. [file JEV2-14-e70207-s015.tif]

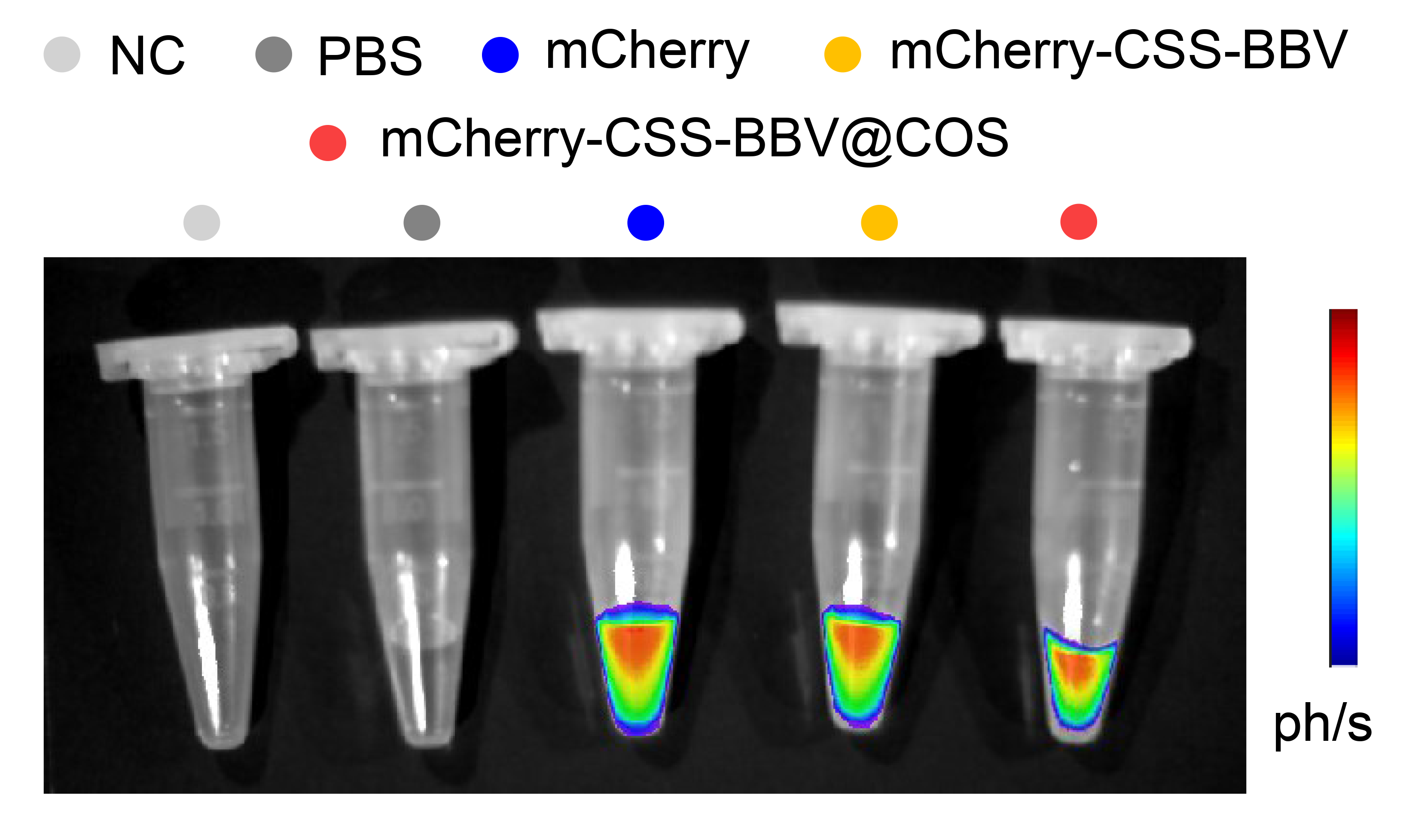

Supplement: Supplementary file 8 — Supplementary Figure 7: In vitro fluorescence imaging of mCherry fluorescence in BBVs. [file JEV2-14-e70207-s013.tif]

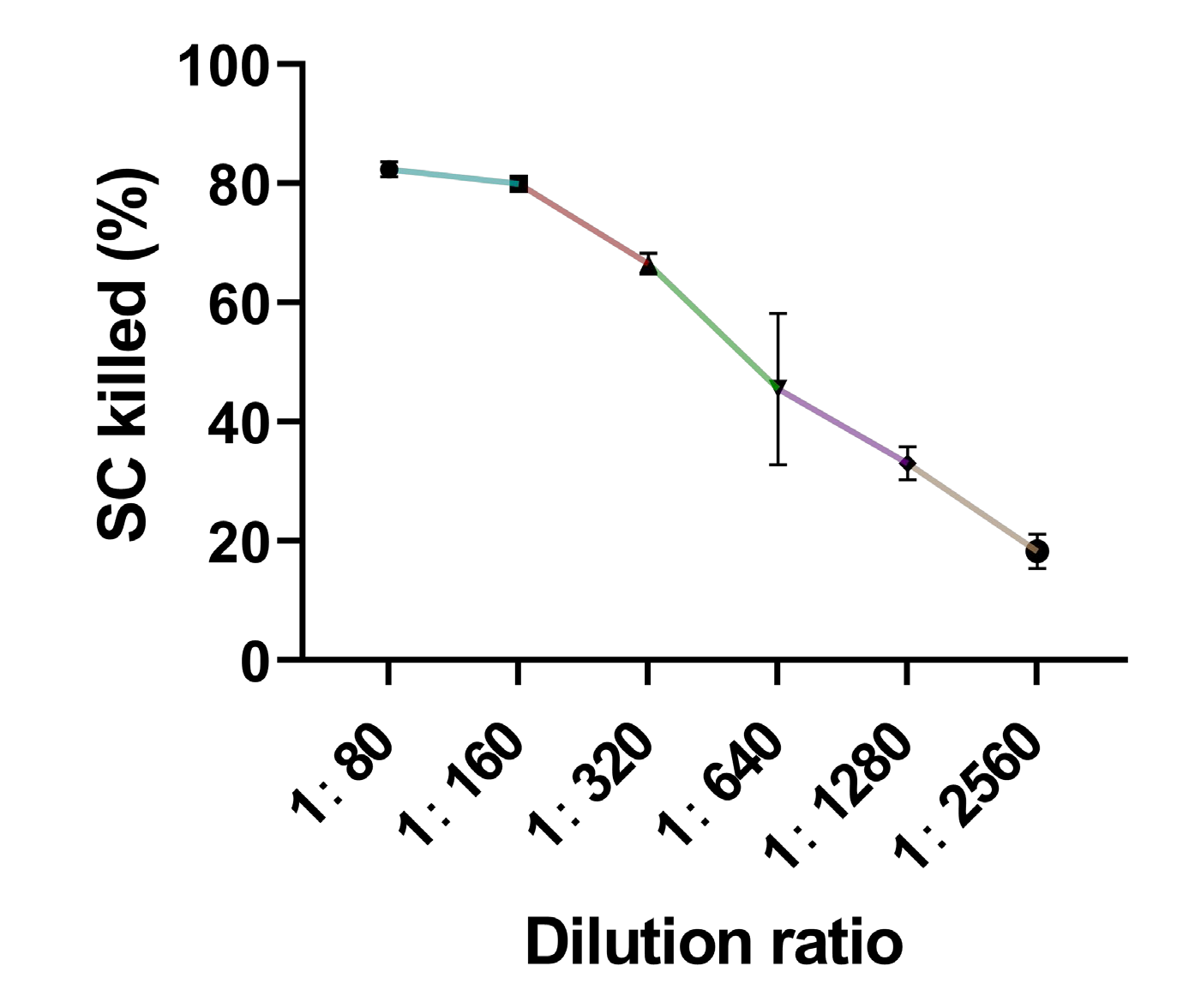

Supplement: Supplementary file 9 — Supplementary Figure 8: In vitro SC bactericidal activity assessment of the week 5 serum at different dilution ratios after immunization with mCherry‐CSS‐BBV@COS. [file JEV2-14-e70207-s012.tif]

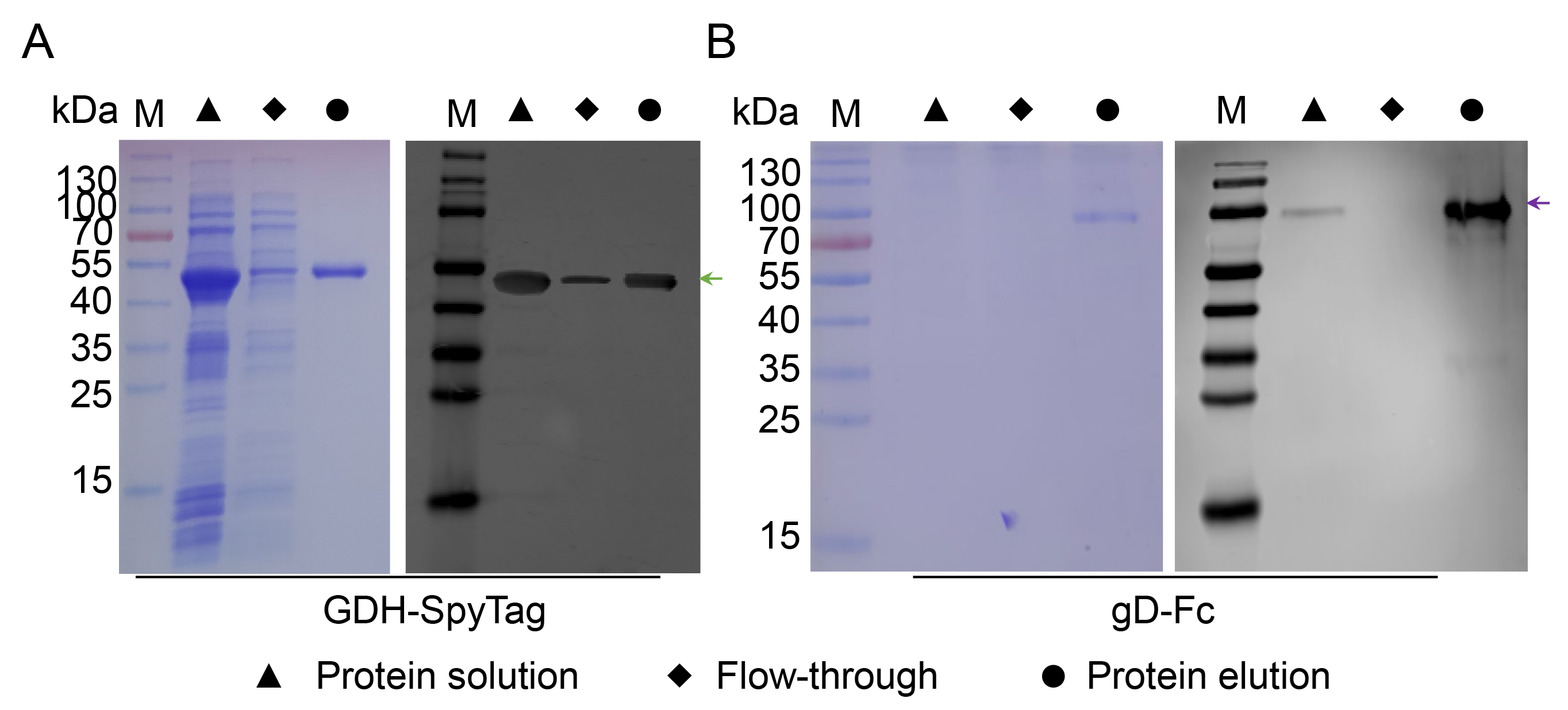

Supplement: Supplementary file 10 — Supplementary Figure 9: Purification and identification of GDH‐SpyTag and gD‐Fc proteins. [file JEV2-14-e70207-s009.tif]

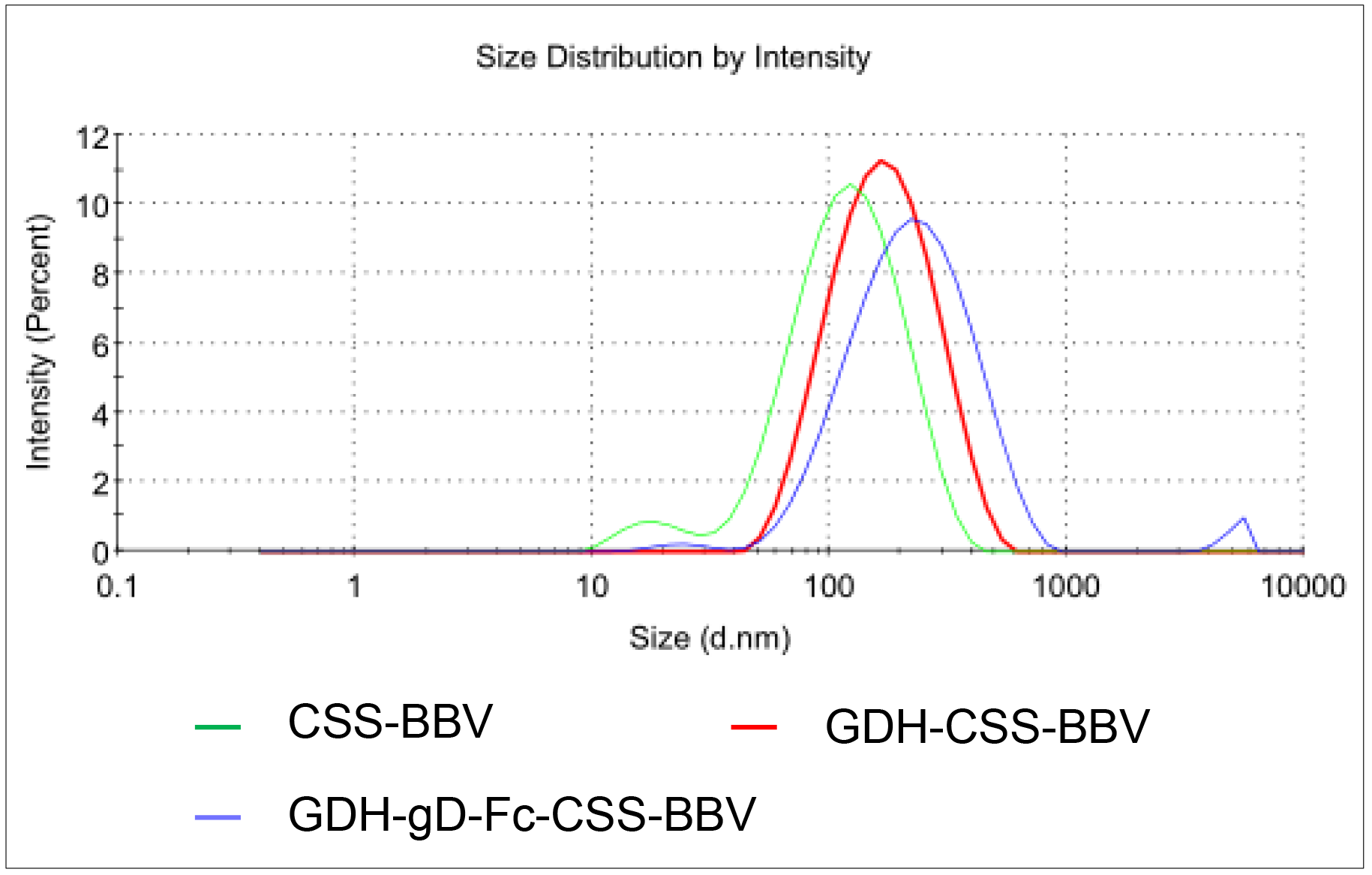

Supplement: Supplementary file 11 — Supplementary Figure 10: Particle size analysis of CSS‐BBVs conjugated with dual antigens. [file JEV2-14-e70207-s017.tif]

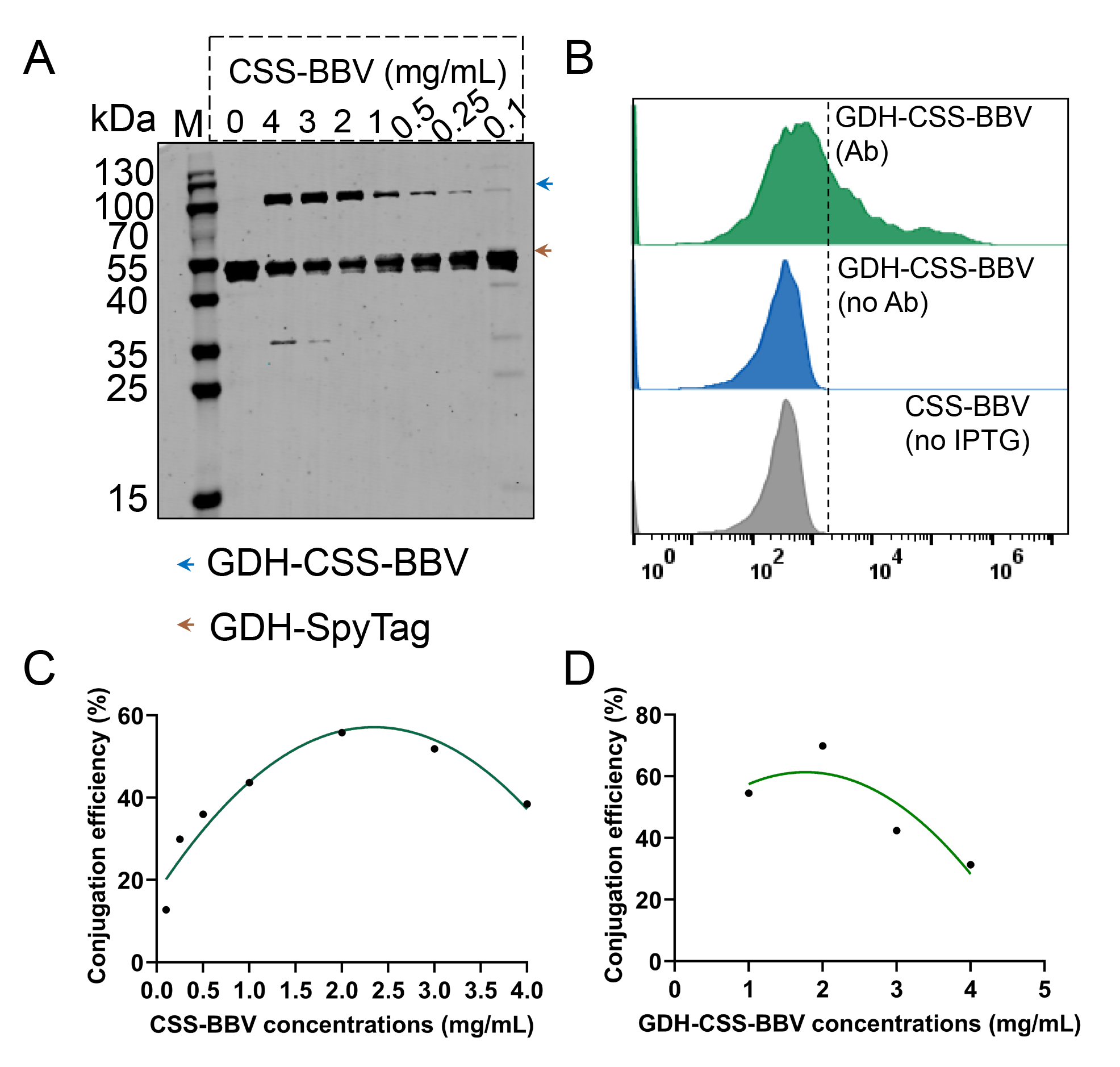

Supplement: Supplementary file 12 — Supplementary Figure 11: Determination of the efficiency of conjugation between CSS‐BBVs and the target protein. [file JEV2-14-e70207-s007.tif]

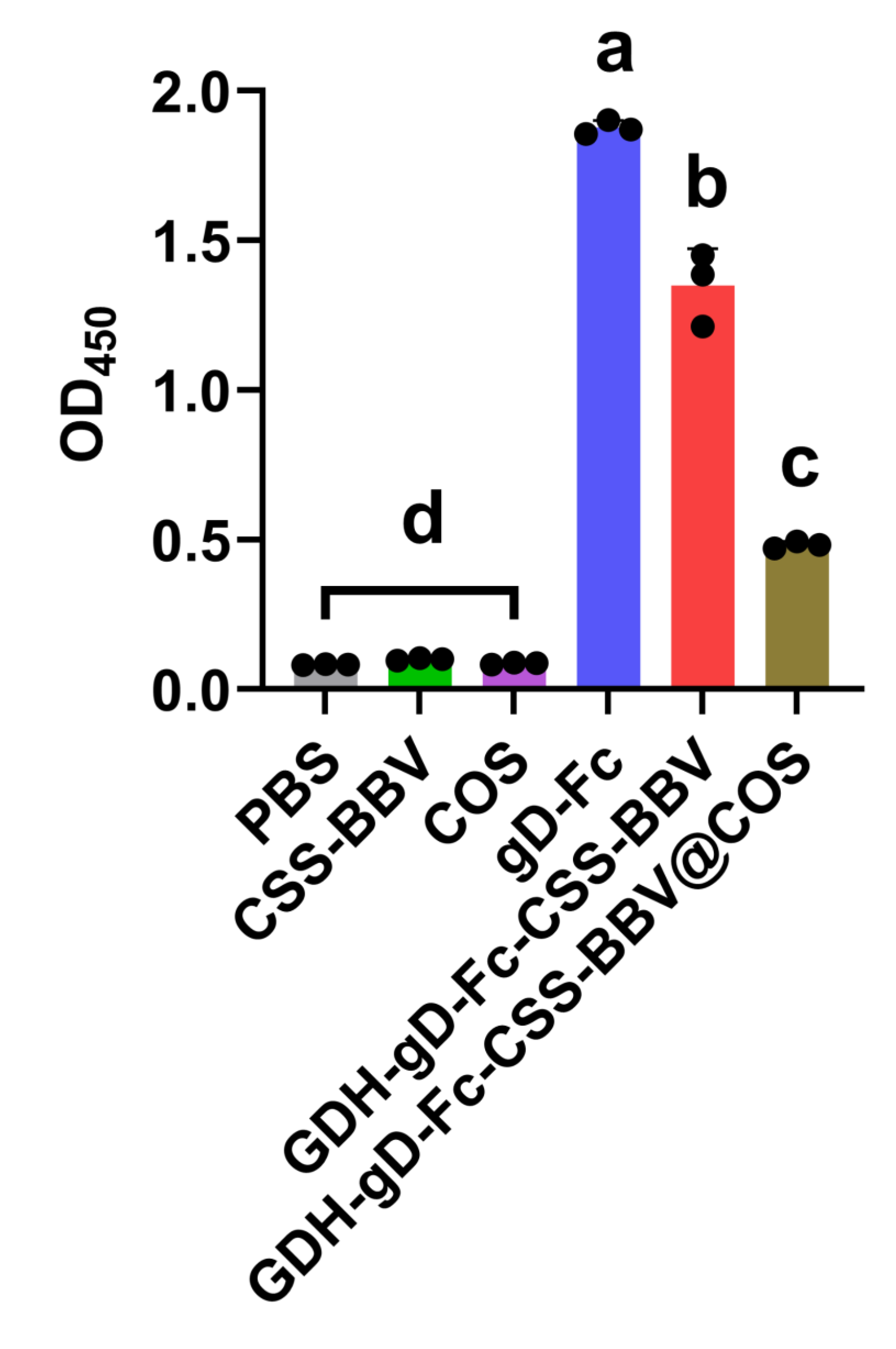

Supplement: Supplementary file 13 — Supplementary Figure 12: ELISA detection of gD‐Fc exposure on the surface of GDH‐gD‐Fc‐CSS‐BBV@COS. [file JEV2-14-e70207-s003.tif]

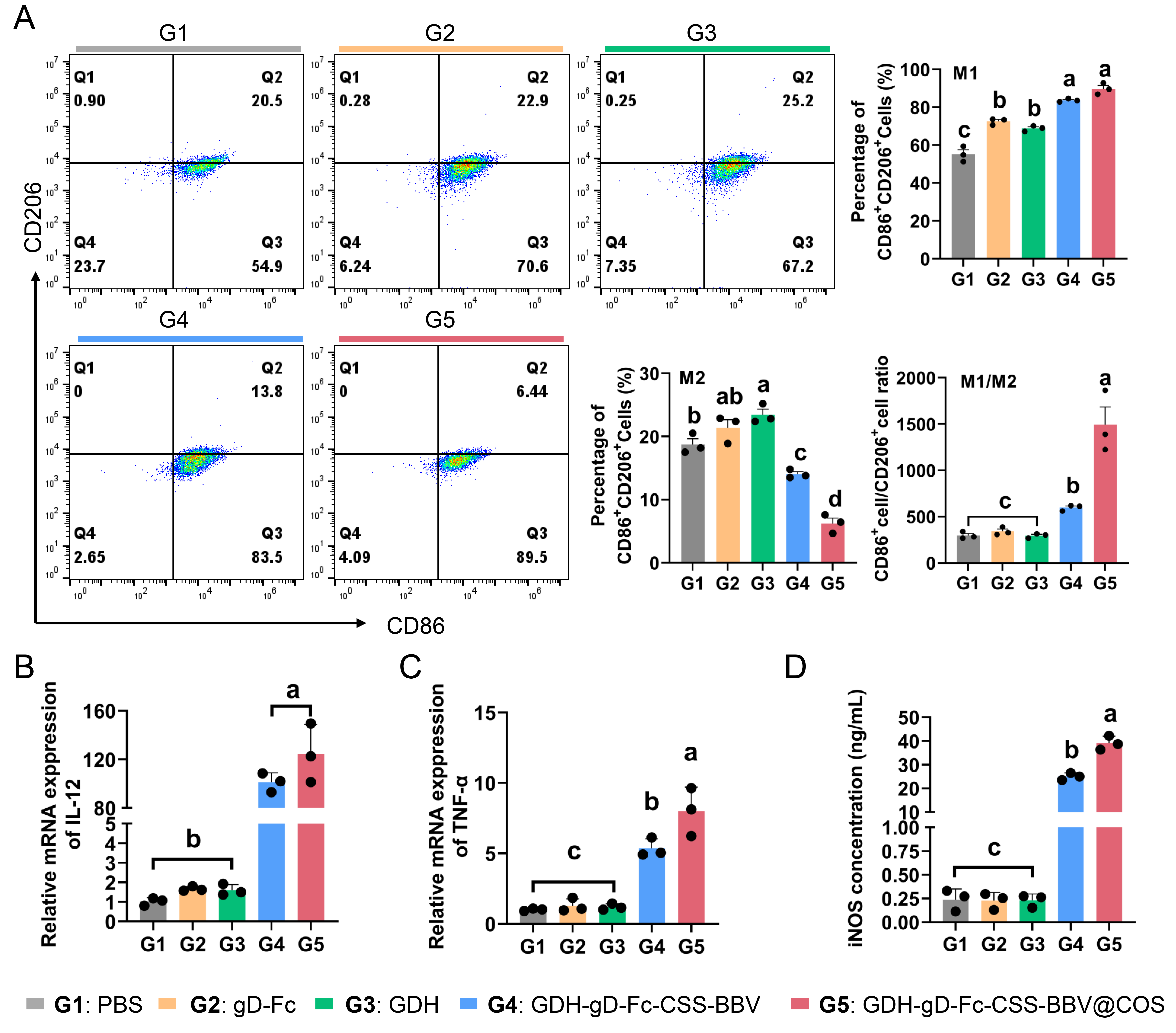

Supplement: Supplementary file 14 — Supplementary Figure 13: Flow cytometry analysis of GDH‐gD‐Fc‐CSS‐BBV@COS promoting M1 polarization in RAW264.7 cells. [file JEV2-14-e70207-s011.tif]

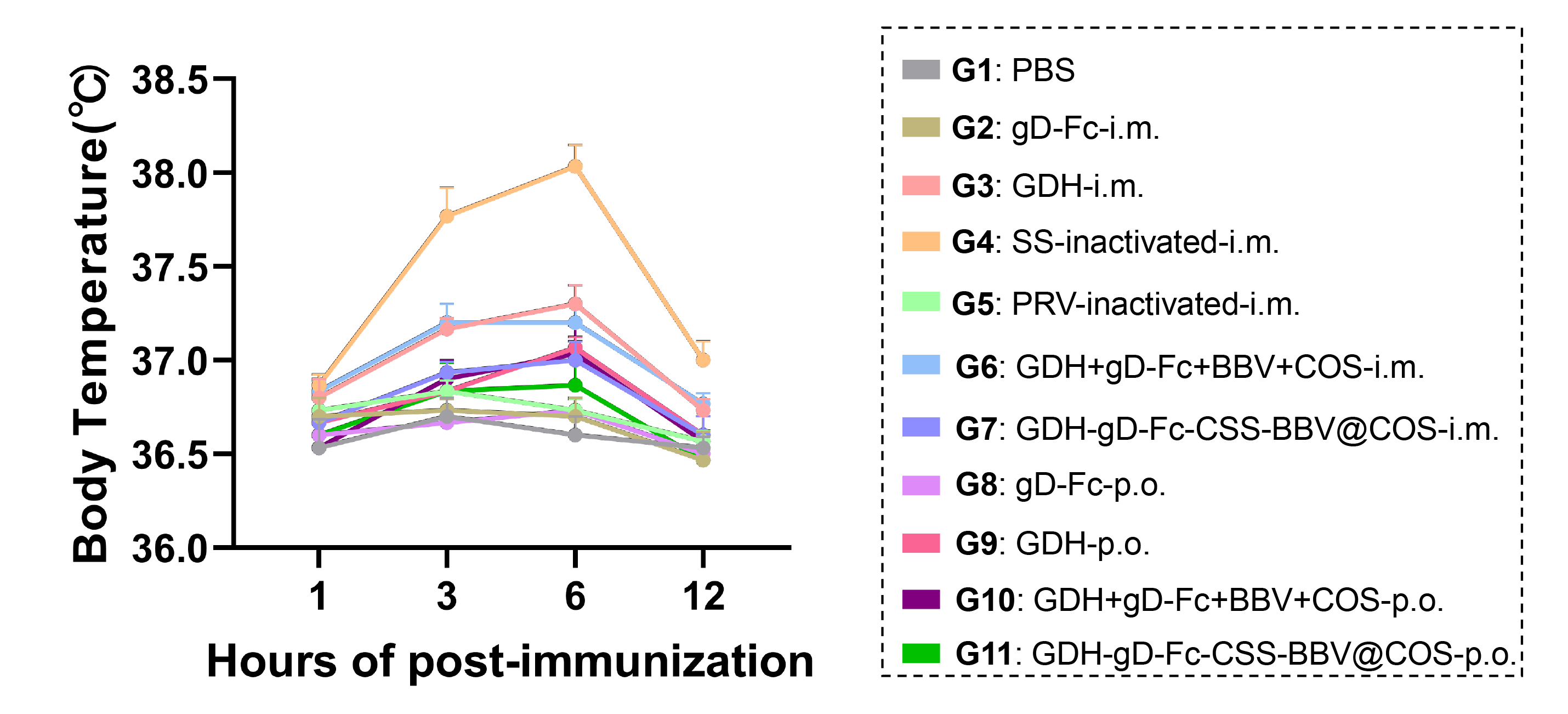

Supplement: Supplementary file 15 — Supplementary Figure 14: Body temperature changes induced by oral immunization with GDH‐gD‐Fc‐CSS‐BBV@COS in mice. [file JEV2-14-e70207-s010.tif]

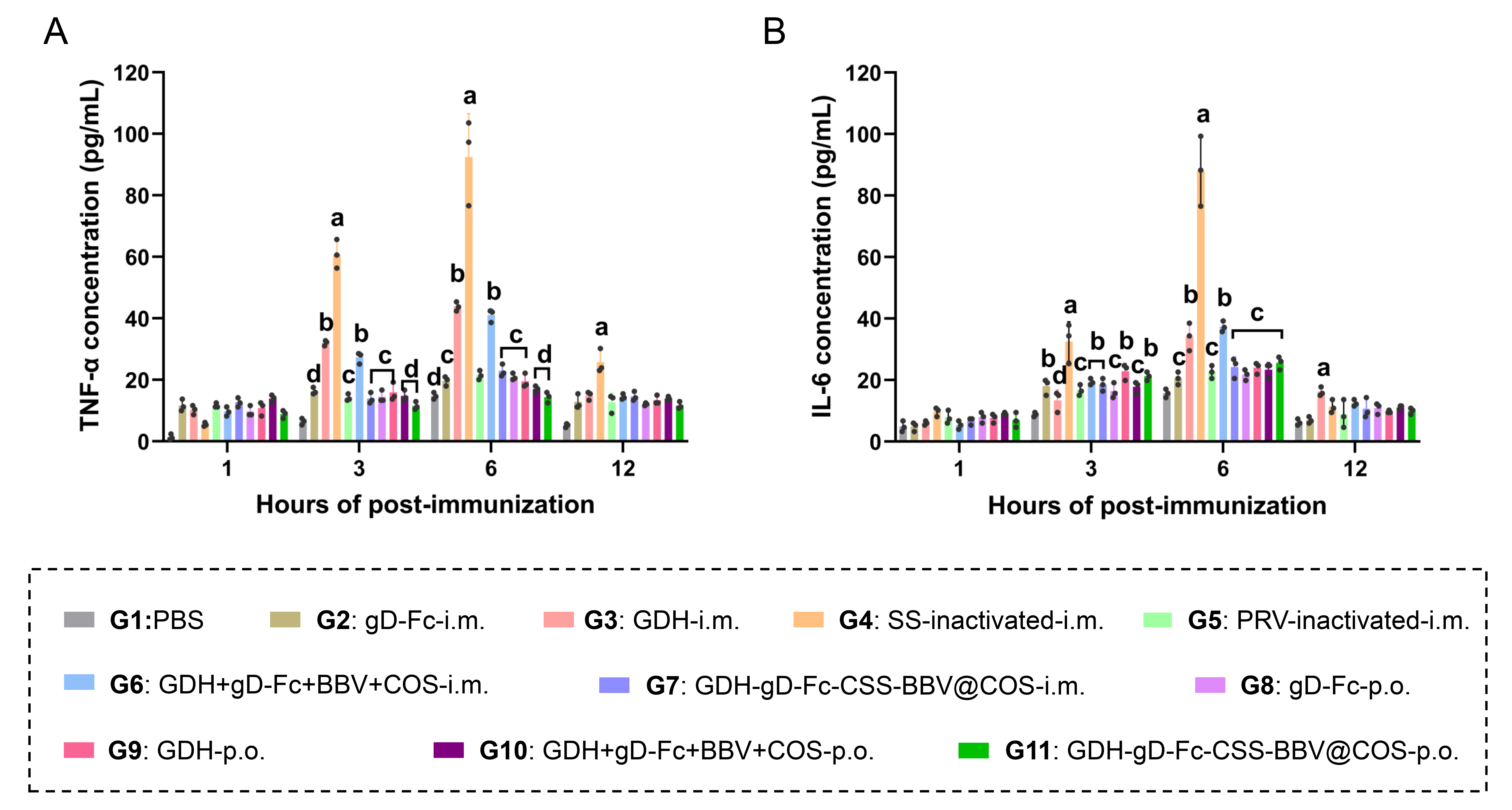

Supplement: Supplementary file 16 — Supplementary Figure 15: Detection of inflammatory factors induced by oral immunization with GDH‐gD‐Fc‐CSS‐BBV@COS in mice. [file JEV2-14-e70207-s014.tif]

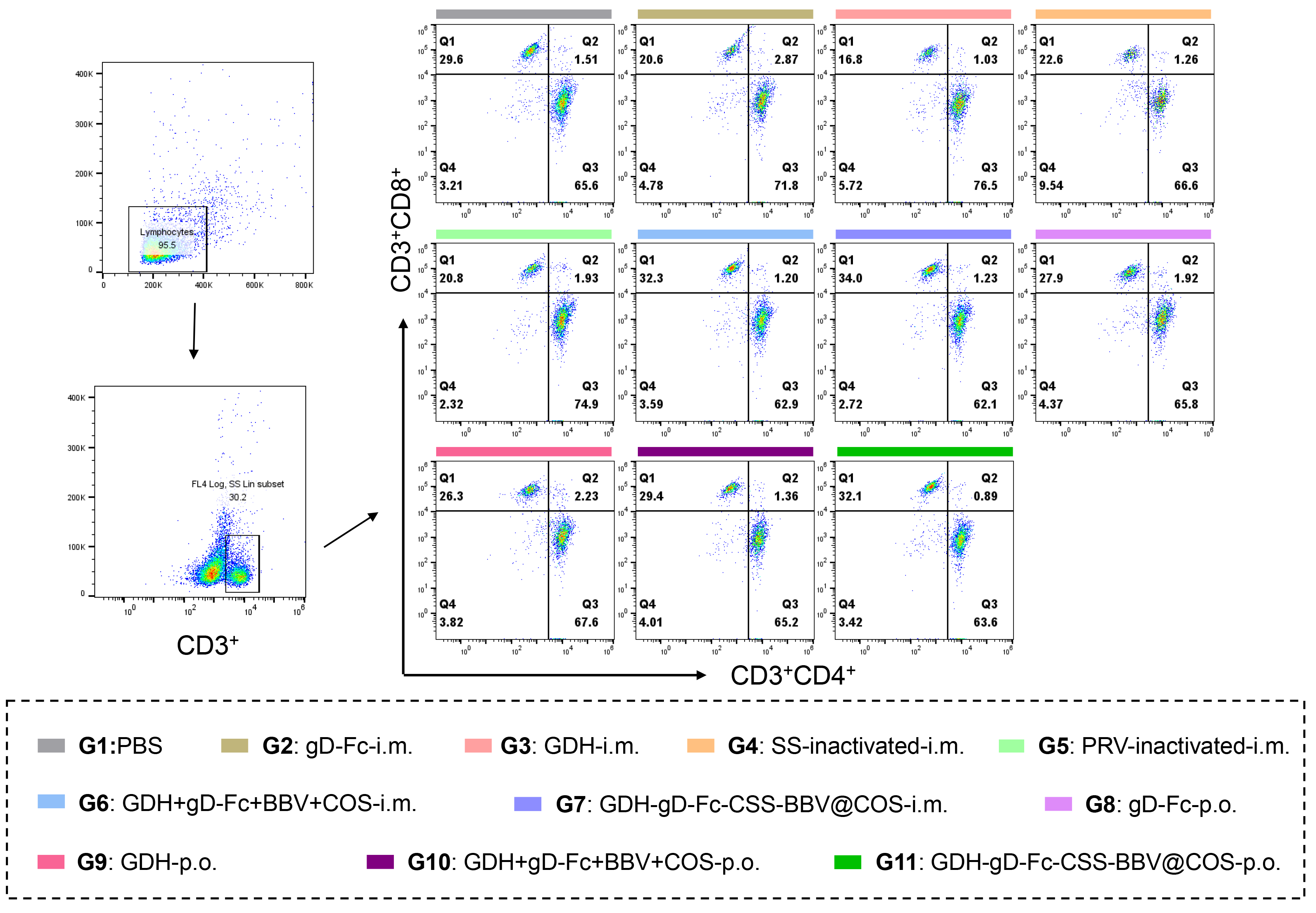

Supplement: Supplementary file 17 — Supplementary Figure 16: Flow cytometry analysis of the proportions of CD3+CD8+ and CD3+CD4+ T cells in the spleen. [file JEV2-14-e70207-s004.tif]

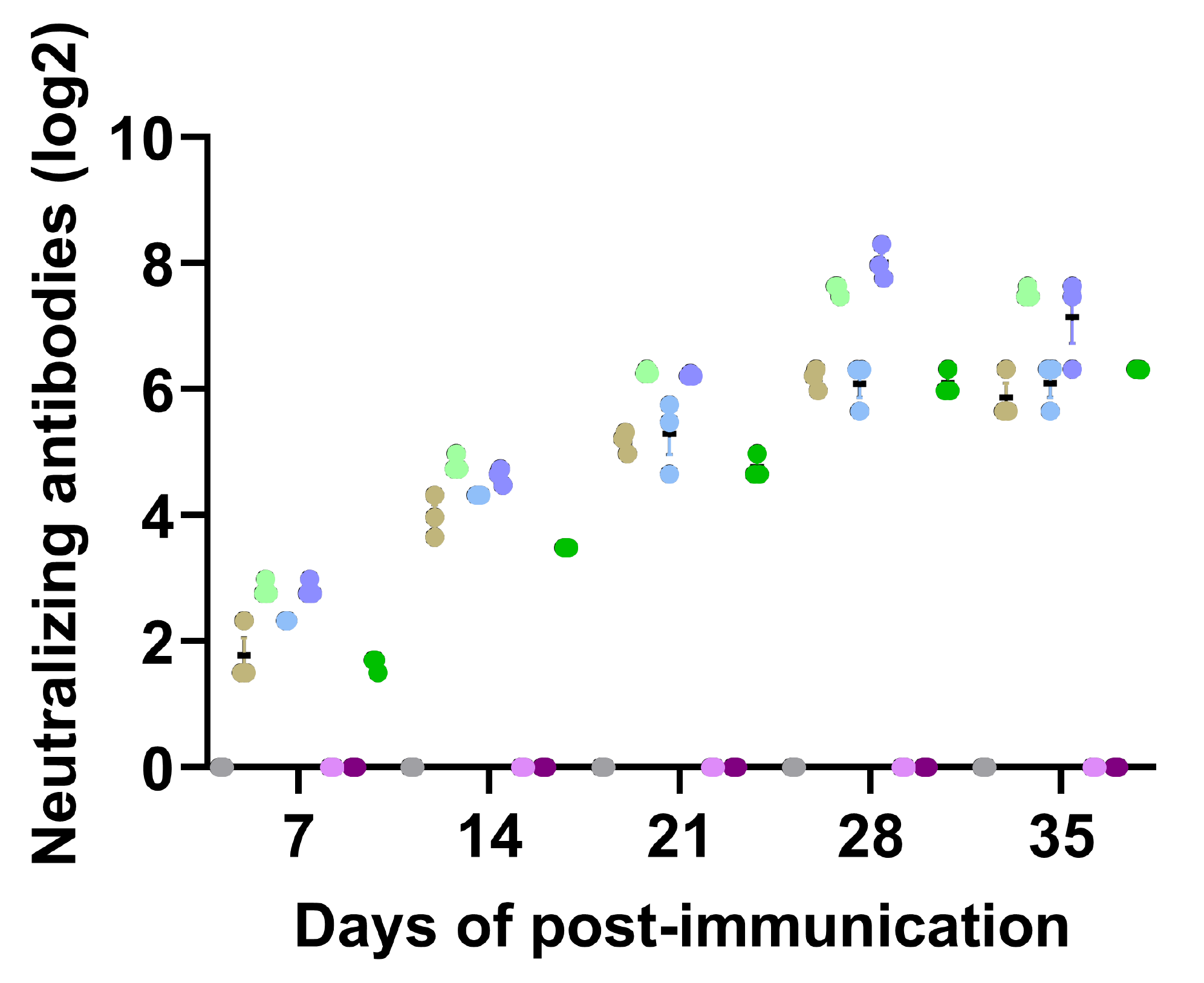

Supplement: Supplementary file 18 — Supplementary Figure 17: Detection of neutralizing antibodies against PRV in the serum of immunized mice; Table S1: Primers used in this study. [file JEV2-14-e70207-s001.tif]
